# Altering patterns of sensorimotor network in patients with different pathological diagnoses and glioma‐related epilepsy under the latest glioma classification of the central nervous system

**DOI:** 10.1111/cns.14109

**Published:** 2023-02-05

**Authors:** Shengyu Fang, Lianwang Li, Shimeng Weng, Yuhao Guo, Xing Fan, Tao Jiang, Yinyan Wang

**Affiliations:** ^1^ Department of Neurosurgery, Beijing Tiantan Hospital Capital Medical University Beijing China; ^2^ Beijing Neurosurgical Institute Beijing China; ^3^ Research Unit of Accurate Diagnosis, Treatment, and Translational Medicine of Brain Tumors, Chinese Academy of Medical Sciences Beijing China

**Keywords:** epilepsy, glioma, magnetic resonance imaging, neural networks

## Abstract

**Aims:**

We aimed to clarify the relationship between alterations in functional networks and glioma‐related epilepsy (GRE) in patients with different molecular diagnoses.

**Methods:**

We enrolled 160 patients with prefrontal gliomas and different histories of GRE. The patients were grouped based on the latest pathological glioma classification and GRE history. Graph theory analysis was applied to reveal alterations in the sensorimotor networks among various subgroups. Binary logistic regression was used to identify risk factors for preoperative GRE onset.

**Results:**

Decreasing shortest path length was found in patients with GRE, regardless of the chromosome 1p/19q status. Nodes located in the premotor and supplementary motor areas showed decreased nodal betweenness centrality and vulnerability in patients with GRE and chromosome 1p/19q intact. Additionally, the node on the primary motor area showed decreased nodal vulnerability but the node on the sensory‐related thalamus increased in patients with GRE and chromosome 1p/19q co‐deletion. Decreased shortest path length, grade 2, and decreased nodal betweenness centrality of the premotor area were risk factors for GRE.

**Conclusion:**

Decreased shortest path length was a characteristic alteration in GRE and prefrontal glioma. Alterations in global properties were similar, but nodal properties were different in patients with GRE and different chromosome 1p/19q statuses.

## Graphical abstract

Glioma with different molecular classification would cause different network alterations that induce preoperative glioma‐related epilepsy. Oligodendroglioma recruited distant cortices but astrocytoma only recruited adjacent cortices to reorganize sensorimotor network when the glioma grew on the prefrontal lobe. In this figure, the green and blue nodes were located on the Brodmann area 6 that altered nodal properties in patients with astrocytoma, whereas the red and yellow nodes were located on the Brodmann area 4 and sensory related thalamus that altered nodal properties in patients with oligodendroglioma. BA, Brodmann area. Nodes No. 1 and No. 5: A6cdl, caudal dorsolateral BA 6; Nodes No. 2 and No. 6: A6m, medial BA 6; Nodes No. 3 and No. 7: A4ul, upper limb region of BA 4; Nodes No. 4 and No. 8: Stha, sensory thalamus.

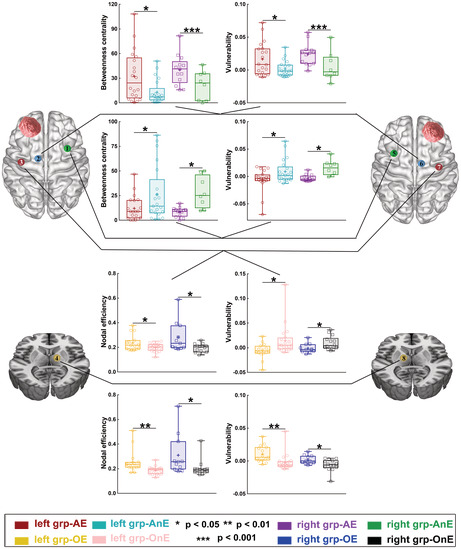

## 1 INTRODUCTION

Glioma‐related epilepsy (GRE) is an abnormal brain network‐like disease.^1^, ^2^, ^3^, ^4^ Glioma with a specific genetic background is susceptible to GRE. However, the relationship between brain network alterations and genetic background in patients with GRE onset is not well known.

Relying on resting‐state functional MRI (rs‐fMRI) and applying topological properties^5^, ^6^ to reveal alterations in the conveying ability of brain‐function networks is valuable for identifying important nodes related to specific symptoms.^7^, ^8^ Previous studies^9^, ^10^, ^11^, ^12^ have demonstrated that different tumor locations cause alterations in different functional networks near the tumor. For instance, increased functional connectivity (FC) of nodes located in the premotor area and decreased shortest path length of the sensorimotor network are specific alterations in patients with prefrontal glioma and GRE onset.^13^

Oligodendroglioma^14^, ^15^ and astrocytoma with isocitrate dehydrogenase (IDH) mutation^16^, ^17^ were considered to be risk factors for GRE onset. One of the potential mechanisms to induce GRE at the microcosmic level was that the d‐2‐hydroxyglutarate products of IDH mutations potentially increase neuronal activity by mimicking the activity of glutamate on the NMDA receptor.^18^ However, at the microcosmic level, the effect of chromosome 1p/19q co‐deletion in causing GRE remains unknown. Simultaneously, the explanation for these phenomena through the sight of alterations of functional network has not been sufficient. At present, adult diffuse glioma was classified based on IDH and chromosome 1p/19q status, and the oligodendroglioma and astrocytoma were both considered IDH mutations according to the latest World Health Organization (WHO) central nervous system (CNS) tumor classification.^19^, ^20^ Hence, investigation the different alterations of functional networks induced by astrocytoma and oligodendroglioma will help reveal the role of chromosome 1p/19q co‐deletion in GRE. Furthermore, due to different molecular diagnoses corresponding to different strategies in glioma patients,^21^ we considered that the therapy for controlling GRE onset might be different.

In this study, a new cohort of patients with prefrontal glioma and different histories of GRE onset compared to those in a previous study was reviewed.^13^ We aimed to (1) validate previous conclusions, (2) determine the relevance of different IDH statuses, functional network alterations, and GRE onset, and (3) investigate specific alterations in patients with a different history of GRE onset and different chromosome 1p/19q status.

## 2 MATERIALS AND METHODS

The study protocol was approved by the local institutional review board.

### 2.1 Participants

Between January 2019 and February 2022, 210 patients from Beijing Tiantan Hospital who had primary prefrontal lobe gliomas were reviewed. The included patients were: (1) adults (older than 18 years), (2) individuals who had undergone >6 years of schooling, and (3) individuals with no history of brain treatment. The exclusion criteria were as follows: (1) head motion >1 mm or rotation >1°, (2) gliomas involving the bilateral prefrontal lobes, (3) lack of information on IDH status or chromosome 1p/19q status, and (4) patients with IDH wild‐type and grade 2/3 glioma.

Finally, 160 patients (men, *n* = 96; left glioma, *n* = 94) were enrolled. Preoperative electroencephalograms were obtained for patients who had an unclear history of seizures (*n* = 62). All patients were first divided into IDH mutation and wild‐type groups. Subsequently, patients in each group were classified into epilepsy and non‐epilepsy groups, based on their history of preoperative GRE (grp‐E, patients with IDH mutation with GRE; grp‐nE, patients with IDH mutation without GRE; grp‐GnE, patients with glioblastoma without GRE; and grp‐GE, patients with glioblastoma with GRE).

To further investigate the differences in topological properties between patients with astrocytoma and oligodendroglioma, patients in each subgroup were divided according to their chromosome 1p/19q status (grp‐AE, patients with astrocytoma (grade 2/3/4) with GRE; grp‐AnE, patients with astrocytoma (grade 2/3/4) without GRE; grp‐OE, patients with oligodendroglioma (grade 2/3) with GRE; and grp‐OnE, patients with oligodendroglioma (grade 2/3) without GRE). Because there were only four patients (one with left glioma and three with right gliomas) in grp‐GE, these patients were excluded from analysis at this time, because the number was too small for statistical analysis.

### 2.2 Molecular pathological diagnosis

To identify the IDH status, genomic DNA was isolated from frozen tissues using a QIAamp DNA Mini Kit (Qiagen), according to the manufacturer's protocol. Pyrosequencing of IDH1/2 mutations was performed using Gene‐Tech (Shanghai, China). For polymerase chain reaction (PCR) amplification, the primers for IDH1 were 5′‐GCTTGTGAGTGGATGGGTAAAAC‐3′ and 5′‐Biotin‐TTGCCAACATGACTTACTTGATC‐3′. The primers for IDH2 were 5′‐ATCCTGGGGGGGACTGTCTT‐3′ and 5′‐Biotin‐CTCTCCACCCTGGCCTACCT‐3′. The primers 5′‐TGGATGGGTAAAACCT‐3′ for IDH1 and 5′‐AGCCCATCACCATTG‐3′ for IDH2 were used for pyrosequencing.

To identify the status of chromosome 1p/19q, the tumor 1p36 and 19q13 statuses were determined using fluorescent in situ hybridization analysis of formalin‐fixed, paraffin‐embedded blocks. At least two neuropathologists from the neuropathology department of the Beijing Neurosurgical Institute confirmed the results.

### 2.3 Clinical information collection

Information regarding preoperative seizures, type of GRE onset, and history of antiepileptic drugs were extracted from inpatient records. Follow‐up information regarding epileptic control was obtained via telephone interviews at 1 year postoperatively.

### 2.4 MRI acquisition

In brief, preoperative (72 h before surgery) and postoperative (within 24 h after surgery) T1‐3D images with gadolinium contrast enhancement and T2 fluid‐attenuated inversion recovery (FLAIR) were used to acquire tumor and tumor resection images. Additionally, echo‐planar imaging was used to acquire rs‐fMRI data. The parameters of these MRI sequences were the same as those previously used^9^, ^13^ and are shown in the Appendix S1.

### 2.5 Functional MRI preprocessing

In brief, rs‐fMRI preprocessing was supported by graph theoretical network analysis (GRETNA) software (https://www.nitrc.org/projects/gretna).^22^, ^23^ The preprocessing pipeline is the same as previously described.^9^ The preprocessing parameters are provided in the Appendix S1.

### 2.6 Regions of tumor invasion and extent of tumor resection

The regions of gliomas with IDH mutation and glioblastoma with IDH wildtype were manually drawn based on T2 FLAIR images and the T1‐contrast enhanced region, respectively. Tumor volume was calculated using the volumetric method, based on the individual tumor masks. Regarding the extent of tumor resection (EOR), the residual tumor was manually drawn by using the same MRI sequence as was used for tumor volume. The EOR was calculated with the following formula^24^:

$$\mathrm{EOR}=1-\frac{\text{Tumor volume}\;\left(\text{post}\right)}{\text{Tumor volume}\;\left(\mathrm{pre}\right)}$$

**FIGURE 1:**
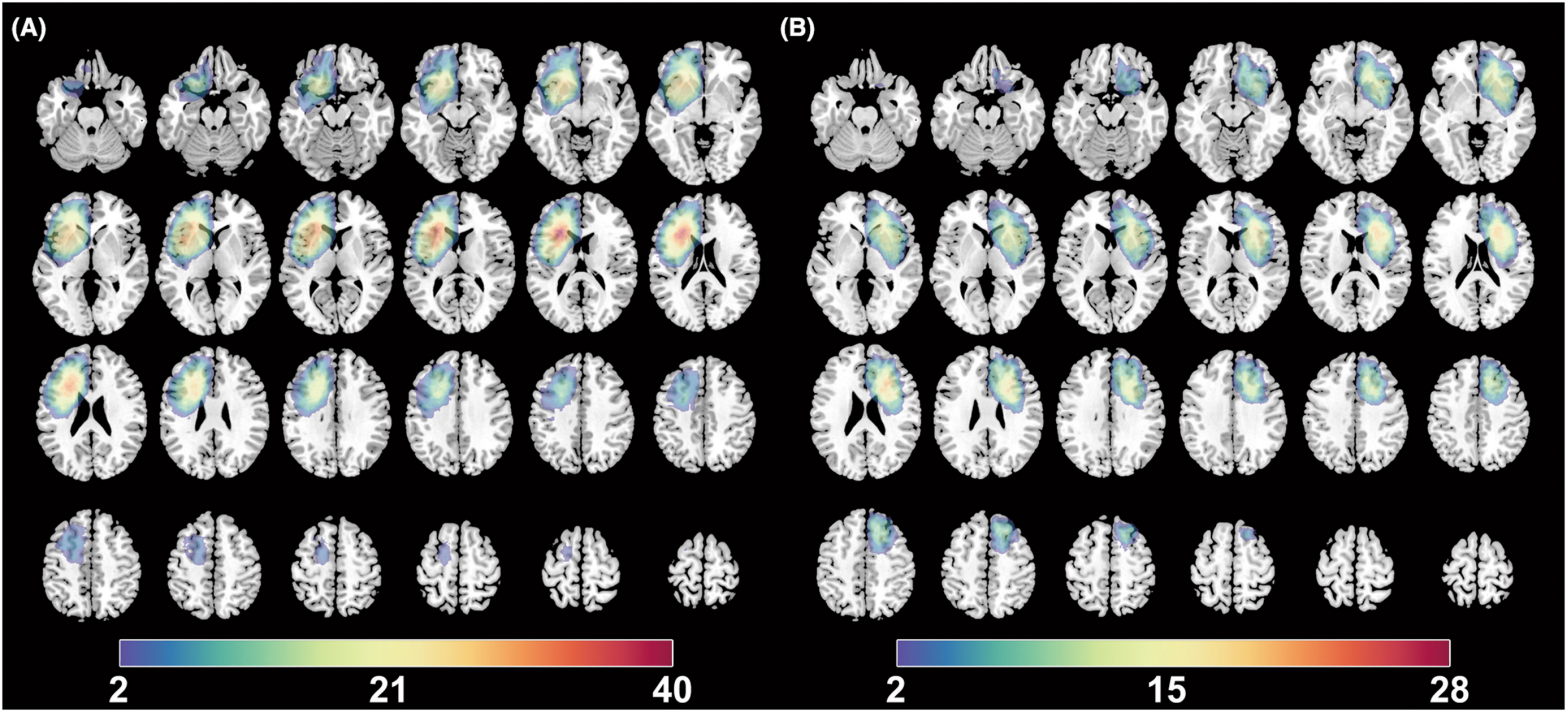
The overlapping results of prefrontal lobe gliomas. The overlapping meant that we overlapped the all tumor masks of left hemisphere into one template to show the quantitative distribution of bilateral hemispheric gliomas. (A) Glioma located in the left hemisphere. (B) Glioma located in the right hemisphere. The value of color bar represents the number of patients with tumor located in a same region.

To show the tumor invasion, the Montreal Neurological Institute standard space was used to normalize all tumor masks using SPM8 (http://www.fil.ion.ucl.ac.uk/spm/software/spm8/) software (Figure 1).

### 2.7 Template for functional connectivity calculation

The sensorimotor template for FC calculation was extracted from the brain atlas “brainnetome 274” (http://www.brainnetome.org/).^25^ To avoid bias during normalization of the individual image to echo‐planar imaging template^26^ caused by tumor invasion, the regions of the extracted sensorimotor template that were invaded by glioma were excluded. Detailed information on the sensorimotor template is provided in the Appendix S1.

### 2.8 Network construction

The FC was calculated using Pearson's correlation analysis, and all FCs were generated into a matrix. To further investigate the topological properties, all negative FC in the matrices were excluded, and a Z‐score transformation was performed for all matrices.

### 2.9 Graph theoretical measures

Global properties were calculated using “brant” software (http://www.brainnetome.org/), including cluster coefficient, fault tolerance, global efficiency, local efficiency, the shortest path length, transformation, and vulnerability. Nodal properties were calculated using GRETNA software, including nodal betweenness, nodal cluster coefficient, nodal degree centrality, nodal efficiency, nodal local efficiency, and nodal vulnerability (using “brant” software).^27^ The number of random networks was 10,000. A well‐acceptable sparsity was applied (from 0.15 to 0.40, interval 0.01).^28^

### 2.10 Statistical analyses

All statistical analyses were performed using SPSS software (25.0 version, IBM®), and statistical figures were generated using GraphPad Prism 8 software (GraphPad Software Inc., San Diego, CA, USA).

According to the type of data, clinical characteristics were compared between grp‐E, grp‐nE, and grp‐GnE using the chi‐squared test, one‐way analysis of variance (ANOVA), and post hoc correction with Sidak correction.

For both global and nodal properties, one‐way ANOVA was first applied to compare grp‐E, grp‐nE, and grp‐GnE. Subsequently, a post hoc analysis with Sidak correction was applied if the results of ANOVA were significantly different (*p* < 0.05). Additionally, based on the significant alterations in topological properties, binary logistic regression with univariate and multivariate (forward: condition) analyses was applied to identify the factors that independently affected preoperative GRE onset. Moreover, to compare differences between grp‐AE and grp‐AnE or between grp‐OE and grp‐OnE, Student's *t*‐test was used. Clinical information (age, sex, and education level) was regressed during the statistical comparison of topological properties.

## 3 RESULTS

### 3.1 Demographic characteristics

**FIGURE 2:**
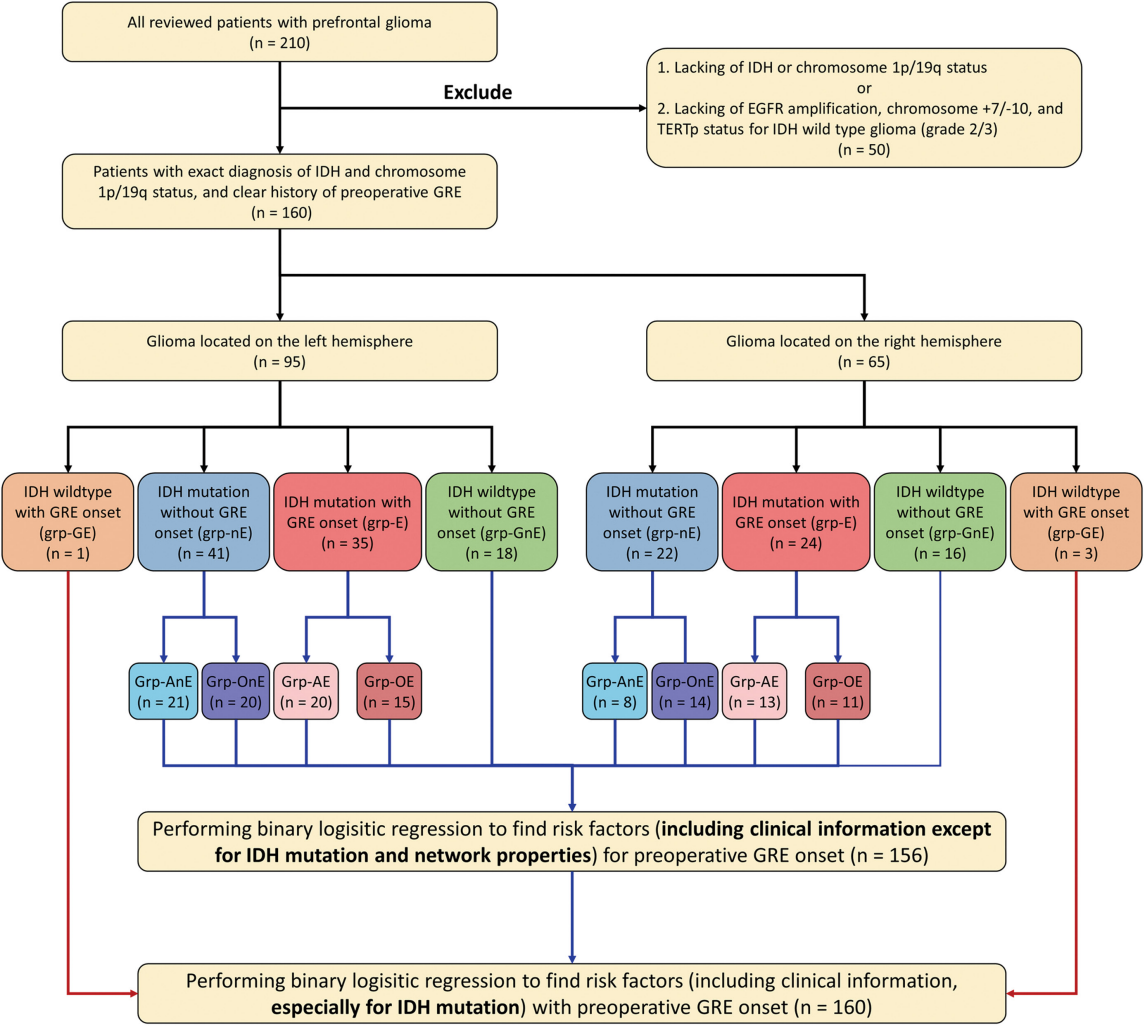
Pipeline of enrolling patients and the number of patients in each subgroup.

The number of patients in each subgroup according to the principle of classification is shown in Figure 2. Except for EOR (left, *p* = 0.0449; right, *p* = 0.0135), no difference in age, sex, education level, and tumor volume was found between the three groups (grp‐E, grp‐nE, and grp‐GnE). Furthermore, our postoperative follow‐up data demonstrated that all patients had Engel Class I in the first year after tumor resection (Table 1).

**TABLE 1:** Demographic and clinical characteristics of patient groups.

Demographic and clinical characteristics	Left glioma (*n* = 94)				Right glioma (*n* = 62)			
	Grp‐E (mean ± SEM)	Grp‐nE (mean ± SEM)	Grp‐GnE (mean ± SEM)	*p* Value	Grp‐E (mean ± SEM)	Grp‐nE (mean ± SEM)	Grp‐GnE (mean ± SEM)	*p* Value
Gender								
Male	24	22	12	0.3662	16	12	10	0.6962
Female	11	19	6		8	10	6	
Age (y)	40.9 ± 1.6	44.5 ± 1.4	44.3 ± 3.0	0.2578	37.9 ± 2.1	44.7 ± 2.3	43.1 ± 2.3	0.0705
Education level (y)	12.1 ± 0.6	11.9 ± 0.6	11.4 ± 0.8	0.8033	12.0 ± 0.7	11.4 ± 0.9	11.8 ± 1.1	0.8940
Tumor grade								
Grade 2	29	34	0	–	18	15	0	–
Grade 3	5	5	0		4	3	0	
Grade 4	1	2	18		2	4	16	
IDH status								
Mutation	35	41	0	–	24	22	0	–
Wildtype	0	0	18		0	0	16	
Chromosome 1p/19q status								
Co‐deletion	15	20	0	–	13	14	0	–
Intact	20	21	18		11	8	16	
Tumor Volume (mm^3^)	26.04 ± 3.19	29.78 ± 2.88	31.46 ± 5.24	0.5637	33.48 ± 4.01	31.47 ± 5.19	37.15 ± 5.48	0.7334
EOR (%)	0.97 ± 0.01	0.96 ± 0.01	0.91 ± 0.01	0.0449	0.97 ± 0.01	0.97 ± 0.01	0.90 ± 0.03	0.0135
Seizure frequency								
Once	26	–	–	–	14	–	–	–
Twice	8	–	–		10	–	–	
More than three times	1	–	–		0	–	–	
Seizure duration before diagnosis (days)	17.6 ± 1.5				16.0 ± 1.7			
Using EEG to diagnose GRE onset or not	3	32	–	–	4	20	3	–
Postoperative GRE control								
Engel I	35	–	–	–	24	–	–	–

*Note*: Age, education level, tumor volume, and EOR were compared by one‐way analysis of variance. Distribution of sex was compared by Chi‐square test.

Abbreviations: EEG, electroencephalograms; EOR, extent of tumor resection; GRE, glioma related epilepsy; Grp‐E, the group of patients with GRE and IDH mutation; Grp‐GnE, the group of patients with non‐GRE and glioblastoma (grade 4); Grp‐nE, the group of patients with non‐GRE and IDH mutation; SEM, standard error mean.

### 3.2 Alterations of global properties in patients with different molecular diagnoses and history of preoperative epilepsy

After post hoc analysis with Sidak correction (Tables S1 and S2), fault tolerance in grp‐E (left glioma, 2.371 ± 0.108; right glioma, 2.047 ± 0.156) was weaker than those in grp‐nE (left glioma, 2.697 ± 0.040, *p* = 0.0071; right glioma, 2.621 ± 0.100, *p* = 0.0049) and grp‐GNE (left glioma, 2.748 ± 0.060, *p* = 0.0153; right glioma, 4.433 ± 0.119, *p* = 0.0014). Similarly, the shortest path length was lower in grp‐E (left glioma, 3.880 ± 0.143; right glioma, 3.477 ± 0.213) than in grp‐nE (left glioma, 4.381 ± 0.066, *p* = 0.0041; right glioma, 4.308 ± 0.121, *p* = 0.0025) and grp‐GnE (left glioma, 4.456 ± 0.139, *p* = 0.0099; right glioma, 4.433 ± 0.119, *p* = 0.0014). Moreover, compared with grp‐E (left glioma, 0.334 ± 0.019; right glioma, 0.396 ± 0.033), global efficiency was weaker in grp‐nE (left glioma, 0.277 ± 0.005, *p* = 0.0057; right glioma, 0.288 ± 0.014, *p* = 0.0064) and grp‐GnE (left glioma, 0.278 ± 0.013, *p* = 0.0452; right glioma, 0.274 ± 0.009, *p* = 0.0047). In addition, compared with grp‐E (left glioma, 0.331 ± 0.019; right glioma, 0.393 ± 0.033), local efficiency was weaker in grp‐nE (left glioma, 0.275 ± 0.005, *p* = 0.0056; right glioma, 0.286 ± 0.014, *p* = 0.0063) and grp‐GnE (left glioma, 0.276 ± 0.013, *p* = 0.0432; right glioma, 0.272 ± 0.009, *p* = 0.0046).

### 3.3 Alterations of nodal properties in patients with different molecular diagnoses and history of preoperative epilepsy

**FIGURE 3:**
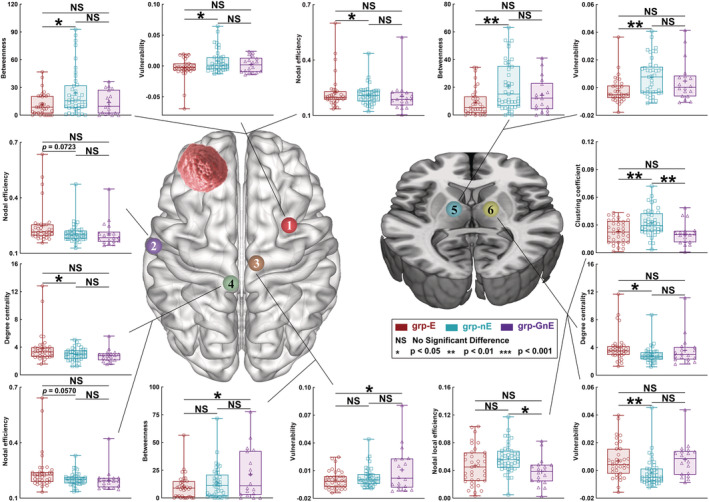
Results of nodal properties in each subgroup when the glioma located in the left prefrontal lobe. The grp‐E (*n* = 35), patients with IDH mutation with GRE; grp‐nE (*n* = 41), patients with IDH mutation without GRE; and grp‐GnE (*n* = 18), patients with glioblastoma without GRE group of patients with glioma‐related epilepsy.

**FIGURE 4:**
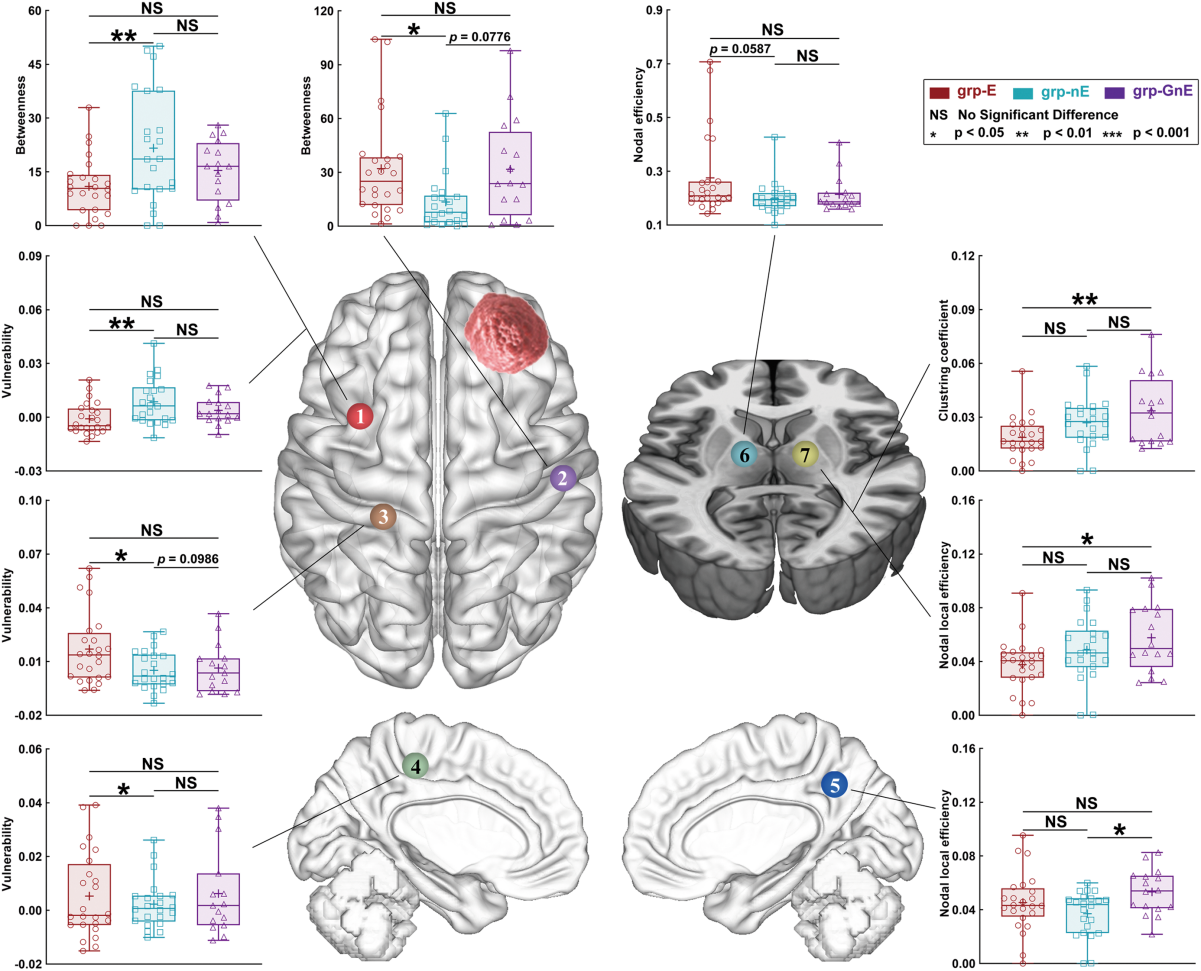
Results of nodal properties in each subgroups when the glioma located in the right prefrontal lobe. The grp‐E (*n* = 24), patients with IDH mutation with GRE; grp‐nE (*n* = 22), patients with IDH mutation without GRE; and grp‐GnE (*n* = 16), patients with glioblastoma without GRE group of patients with glioma‐related epilepsy.

After Sidak correction, several significant alterations of nodal properties were found between the three groups (grp‐E, grp‐nE, and grp‐GnE; Tables S3–S14; Figures 3 and 4). However, only one node (caudal dorsolateral Brodmann area (BA) 6 (A6cdl) on the contralesional hemisphere) had the same tendency of nodal properties altering, regardless of the tumor hemisphere. Regarding this node, the nodal betweenness in the grp‐E (left glioma, 11.693 ± 1.997; right glioma, 10.947 ± 1.656) was lower than that in grp‐nE (left glioma, 23.916 ± 3.813, *p* = 0.0171; right glioma, 21.636 ± 3.296, *p* = 0.0084; Sidak correction). In addition, the nodal vulnerability in grp‐E (left glioma, −0.003 ± 0.003; right glioma, −0.001 ± 0.002) was lower than that in grp‐nE (left glioma, 0.008 ± 0.003, *p* = 0.0168; right glioma, 0.009 ± 0.003, *p* = 0.0072; Sidak correction).

### 3.4 Alterations of global properties in patients with different chromosome 1p/19q co‐deletion status and history of preoperative epilepsy

Regarding patients with IDH mutation and chromosome 1p/19q intact, after Student's *t*‐test comparison (Table S15), global efficiency was higher in grp‐AE (left glioma, 0.330 ± 0.024; right glioma, 0.392 ± 0.039) than in grp‐AnE (left glioma, 0.277 ± 0.008, *p* = 0.0462; right glioma, 0.277 ± 0.012, *p* = 0.0419). Moreover, local efficiency was higher in grp‐AE (left glioma, 0.328 ± 0.024; right glioma, 0.389 ± 0.039) than in grp‐AnE (left glioma, 0.275 ± 0.008, *p* = 0.0461; right glioma, 0.275 ± 0.012, *p* = 0.0421). Additionally, the shortest path length was lower in grp‐AE (left glioma, 3.908 ± 0.189; right glioma, 3.424 ± 0.273) than in grp‐AnE (left glioma, 4.391 ± 0.098, *p* = 0.0310; right glioma, 4.372 ± 0.139, *p* = 0.0229).

Regarding patient with IDH mutation and chromosome 1p/19q co‐deletion, no alterations of global property were found simultaneously significant both in the group of left and right gliomas (Table S16). Global efficiency and local efficiency were higher in grp‐OE (global efficiency: 0.339 ± 0.031; local efficiency: 0.276 ± 0.006) than in grp‐OnE (global efficiency: 0.336 ± 0.030, *p* = 0.0335; local efficiency: 0.274 ± 0.005, *p* = 0.0333) when the glioma was located on the left. Moreover, the shortest path length was lower in grp‐OE (3.842 ± 0.226) than in grp‐OnE (4.370 ± 0.087, *p* = 0.0263). Regarding right hemispheric glioma, the clustering coefficient was lower in grp‐OE (0.063 ± 0.011) than in grp‐OnE (0.092 ± 0.008, *p* = 0.0350).

### 3.5 Alterations of nodal properties in patients with different chromosome 1p/19q co‐deletion status and history of preoperative epilepsy

Regarding grp‐AE and grp‐AnE, after Student's *t*‐test comparison, several significant alterations of nodal properties were found (Tables S17–S22). However, only two nodes (medial BA 6 (A6m) on the lesional hemisphere and A6cdl on the contralesional hemisphere) had the same tendency of nodal properties altering, regardless of the tumor hemisphere.

In the A6m, nodal betweenness in grp‐AE (left glioma, 32.348 ± 6.606; right glioma, 40.145 ± 5.108) was higher than that in grp‐AnE (left glioma, 12.984 ± 3.112, *p* = 0.0123; right glioma, 21.310 ± 6.299, *p* = 0.0328). Similarly, nodal vulnerability in grp‐AE was higher (left glioma, 0.017 ± 0.005; right glioma, 0.024 ± 0.004) than that in grp‐AnE (left glioma, 0.001 ± 0.003, *p* = 0.0166; right glioma, 0.005 ± 0.008, *p* = 0.0429). In addition, in the A6cdl, nodal betweenness in grp‐E (left glioma, 12.052 ± 2.681; right glioma, 8.331 ± 1.340) was lower than that in grp‐nE (left glioma, 26.223 ± 5.896, *p* = 0.0423; right glioma, 28.330 ± 5.655, *p* = 0.0004). Similarly, nodal vulnerability in grp‐AE was lower (left glioma, −0.006 ± 0.004; right glioma, −0.003 ± 0.002) than that in grp‐AnE (left glioma, 0.009 ± 0.005, *p* = 0.0283; right glioma, 0.016 ± 0.005, *p* = 0.0004).

Regarding grp‐OE and grp‐OnE, after Student's *t*‐test comparison, several significant alterations of nodal properties were found (Tables S23–S28). However, two nodes (upper limb of BA 4 (A4ul) on the lesional hemisphere and sensory thalamus (Stha) on the contralesional hemisphere) had the same tendency of nodal properties altering, regardless of the tumor hemisphere.

In the A4ul, nodal efficiency in grp‐OE (left glioma, 0.236 ± 0.016; right glioma, 0.283 ± 0.036) was higher than that in grp‐OnE (left glioma, 0.198 ± 0.007, *p* = 0.0318; right glioma, 0.194 ± 0.009, *p* = 0.0186). Conversely, nodal vulnerability in grp‐OE (left glioma, −0.006 ± 0.004; right glioma, −0.001 ± 0.003) was lower than that in grp‐OnE (left glioma, 0.015 ± 0.007, *p* = 0.0227; right glioma, 0.010 ± 0.004, *p* = 0.0372). Moreover, in the Stha, nodal efficiency in grp‐OE (left glioma, 0.255 ± 0.023; right glioma, 0.310 ± 0.047) was higher than that in grp‐OnE (left glioma, 0.186 ± 0.007, *p* = 0.0041; right glioma, 0.202 ± 0.018, *p* = 0.0362). Similarly, nodal vulnerability in grp‐OE (left glioma, 0.010 ± 0.003; right glioma, 0.002 ± 0.002) was higher than that in grp‐OnE (left glioma, −0.002 ± 0.003, *p* = 0.0053; right glioma, −0.006 ± 0.002, *p* = 0.0246).

### 3.6 Factors of preoperative GRE onset

Regarding all patients, age, IDH mutation, and histopathology grade 2 (reference: not grade 2) were potential risk factors for GRE onset in the univariate analysis. Finally, IDH mutation (*p* < 0.001; odd ratio (OR) = 7.960; 95% confidence interval (CI), 2.633–23.799, Table S29) was an independent risk factor for GRE onset in the multivariate analysis. Moreover, excluding patients in grp‐GE and adding topological properties to the analysis, the results of univariate analysis showed that age, histopathological grade 2 (reference: not grade 2), shortest path length, nodal betweenness of A6cdl, and nodal vulnerability of A6cdl were potential risk factors for GRE onset. Finally, grade 2 tumors (*p* < 0.001; OR = 5.107; 95% CI, 2.081–12.532), decreased shortest path length (*p* < 0.001; OR = 0.244; 95% CI, 0.127–0.467), and decreasing nodal betweenness of A6cdl (*p* = 0.002; OR = 0.954; 95% CI, 0.927–0.982) were risk factors for preoperative GRE onset (Table 2).

**TABLE 2:** Factors for preoperative GRE onset through binary logistic regression analysis without patients in the grp‐GE.

Demographic and clinical characteristics	Univariate analysis			Multivariate analysis		
	*p* Value	Odd ratio	95% CI	*p* Value	Odd ratio	95% CI
Age	0.005	0.953	0.921 to 0.986	–	–	–
Histopathological grade (reference: not grade 2)	0.001	3.326	1.598 to 6.924	<0.001	5.107	2.081 to 12.532
Chromosome 1p/19q status (reference: intact)	0.263	1.460	0.753 to 2.830	–	–	–
Tumor volume	0.352	0.992	0.977 to 1.008	–	–	–
Shortest path length	<0.001	0.274	0.153 to 0.489	<0.001	0.244	0.127 to 0.467
Nodal betweenness of A6cdl	0.003	0.959	0.933 to 0.986	0.002	0.954	0.927 to 0.982
Nodal vulnerability of A6cdl	0.002	1.5 × 10^−21^	4.44 × 10^−36^ to 5.18 × 10^−9^	–	–	–

*Note*: A6cdl = Caudal dorsolateral Brodmann area 6 on the contralesional hemisphere. Multivariate analysis was: forward, condition. grp‐GE, patients with IDH wildtype and glioma‐related epilepsy onset.

## 4 DISCUSSION

In this study, we investigated alterations in functional networks in glioma patients with preoperative GRE onset and further investigated specific alterations in patients with different pathologies, based on the latest version of the molecular pathological classification.^19^, ^20^ Our findings indicated that decreasing the shortest path length was a common alteration in patients with GRE onset, regardless of molecular pathological diagnosis. However, alterations in nodal properties in BA 6 were specific to patients with astrocytoma. Moreover, alterations in nodal properties in BA 4 and the sensory‐related thalamus were specific for patients with oligodendroglioma.

In our study, the ratio of preoperative GRE onset was 39.4%, which is lower than those in previous reports.^14^, ^29^ The tumor was far from the eloquent area, which is the main reason for this phenomenon. Pallud et al.^29^ found that the risk ratio of GRE onset was 2.89 times higher when the glioma was close to the eloquent area^30^ than when it was distant from the eloquent area. In this study, all tumors were located in the prefrontal lobe, which is distant from the sensorimotor cortices. Hence, compared to reports^29^, ^31^ of frontal gliomas close to the eloquent area, our ratio of GRE onset is low.

Preoperative GRE onset was related to IDH mutation but was not related to chromosome 1p/19q co‐deletion. The mechanism by which IDH mutation induced preoperative GRE onset was the product of d‐2‐hydroxyglutarate activity in glioma, with IDH mutation potentially increasing neuronal activity by mimicking the activity of glutamate on the NMDA receptor.^32^ When we analyzed all patients (160 patients in all sub‐groups) using their clinical information alone (including molecular histopathology data) (Table S29), we found that IDH mutation was an independent risk factor for GRE onset, but the chromosome 1p/19q co‐deletion was not independent. Hence, our findings indicate that the role of IDH mutation^16^ in predicting GRE onset was more important than that of chromosome 1p/19q codeletion,^33^ based on the histopathological diagnosis. Additionally, our findings do not contradict those of previous studies^34^, ^35^ that showed that oligodendroglioma was susceptible to the induction of GRE onset. Hence, under the latest glioma classification, oligodendrogliomas are classified as IDH mutation.^19^, ^20^, ^36^

Decreased shortest path length was an important factor that induced GRE onset in prefrontal glioma.^13^ We enrolled a new cohort in which the number of patients was more than twice that in the previous study,^13^ and verified that decreasing shortest path length was an independent risk factor for GRE onset. The shortest path length means the lowest cost during the conveying of information in a network.^9^ The decreased shortest path length was advantageous for conveying information, but it reduced the threshold of epilepsy onset.^37^ Hence, patients with a decreasing shortest path length are susceptible to GRE onset. Due to the small sample size of patients in grp‐GE (patients with glioblastoma and GRE onset), in our analysis of network properties, all patients with GRE had IDH mutation (Table 1). IDH mutation facilitated anaerobic glycolysis, which works against network stability.^38^ In other words, it was easier for IDH mutation to induce network alterations than it was for IDH wildtype. Network alterations increased the probability that the originally stable network shortened the path length. It also explains why patients with IDH mutation were susceptible to GRE onset.

In addition, the contralesional node A6cdl was located on the premotor area that is responsible for integrating and controlling motor function.^39^ Nodal betweenness centrality represents the number of shortest pathway through a node,^40^ and nodal vulnerability represents the altering ratio of network efficiency if the node is removed.^10^ Our results showed that the betweenness and vulnerability of node A6cdl decreased in grp‐E compared to grp‐nE. These findings indicate that the importance of this node was reduced in patients with GRE compared to those with non‐GRE for glioma with IDH mutation, and this also signifies that if the glioma weakened motor control, GRE would occur more easily. Nevertheless, these properties in grp‐E are not different from those in grp‐GnE. Patients in grp‐GnE did not experience GRE onset. Tumor grade is the major reason for this finding. Based on the results of logistic analysis, if the tumor is of grade 2, the risk of GRE onset would be over five times more than it would be for tumors of a higher grade. This result is consistent with those of previous studies that showed that high‐grade glioma is less likely to cause GRE.^14^

Although chromosome 1p/19q co‐deletion was not an independent risk factor for GRE onset, changes in the sensorimotor network differed between patients with GRE onset in grp‐AE (chromosome 1p/19q intact) and grp‐OE (chromosome 1p/19q co‐deletion). Because the thalamus and cingulate cortices are related to sensorimotor functions,^41^ we added these regions to the sensorimotor network to investigate alterations in topological properties in distant regions. Our findings indicated that, regarding patients in grp‐AE, the node Am6 (lesional hemispheric supplementary motor area) and node A6cdl (contralesional hemispheric premotor area) weaken their control in the sensorimotor network because their nodal betweenness centrality and nodal vulnerability decreased. The weakened control in the sensorimotor network would cause GRE onset.^13^ Notably, these two nodes were near the tumor. However, compared to grp‐AE, in grp‐OE, both the node (A4ul) near the tumor and that (Stha) far from the tumor showed alterations in nodal properties. These two nodes were located in the lesional hemispheric primary motor area (A4ul) and the contralateral hemispheric sensory‐related thalamus (Stha). We believe the different alterations between grp‐AE and grp‐OE might be related to the different histopathological characteristics and mechanisms of network reorganization. Oligodendroglioma grows more slowly than astrocytoma.^42^ Slow tumor growth was an advantage for network reorganization.^43^ Based on the classical theory of network reorganization, the surrounding tissue first participates in network reorganization before the distant area.^44^ Hence, our findings support the concept that glioma with different molecular classification would cause different network alterations.

Our findings built a bridge between network alterations, specific symptoms, and molecular pathological diagnosis. Additionally, they provided some evidence pertaining to potential targets of intervention, for instance, A4ul on the lesional hemisphere for patients with oligodendroglioma and A6cdl on the contralesional hemisphere for patients with astrocytoma, to be effectively treated with neuro‐electro‐physiological therapy, such as via transcranial magnetic stimulation^45^ and transcranial electrical stimulation.^46^

However, a major limitation of the study is that we excluded patients with IDH wildtype grade 2/3 because of a lack of information on epidermal growth factor receptor amplification, chromosome +7/−10, and telomerase reverse transcriptase status. In the future, we will obtain this information and investigate network alterations.

## 5 CONCLUSION

IDH mutation was meaningful to induce preoperative GRE onset. Moreover, decreasing shortest path length was a common characteristic alteration of GRE onset for patients with IDH mutation. Importantly, the GRE onset‐related alterations of the nodes in the sensorimotor network were different in patients with different chromosome 1p/19q statuses.

## AUTHOR CONTRIBUTIONS

Study concept and design: Fang SY, Li LW, Weng SM, and Guo YH. Data acquisition and analysis: Fang SY, Li LW, Weng SM, and Guo YH. Statistics/verified analytical method: Fang SY and Wang YY. Writing the first draft: Fang SY and Wang YY. Supervision study: Fan X, Wang YY, and Jiang T. Read and approved final version: All authors.

## FUNDING INFORMATION

This work was supported by the Public Welfare Development and Reform Pilot Project of Beijing Medical Research Institute (PXM2019_026280_000008), Beijing Municipal Natural Science Foundation (No. 7202021), National Natural Science Foundation of China (No. 82001777), and Research Unit of Accurate Diagnosis, Treatment, and Translational Medicine of Brain Tumors Chinese (No. 2019‐I2M‐5‐021).

## CONFLICT OF INTEREST STATEMENT

The authors declare no competing financial interests.

## Supporting information


Appendix S1

## Data Availability

Anonymized data will be made available on request.
