# Supplementary material for: Altering patterns of sensorimotor network in patients with different pathological diagnoses and glioma‐related epilepsy under the latest glioma classification of the central nervous system
Source: CNS Neurosci Ther. 2023 Feb 5;29(5):1368–78. doi: 10.1111/cns.14109 (PMC10068458; doi:10.1111/cns.14109)
Supplement: Supplementary file 1 — Appendix S1 [file CNS-29-1368-s001.docx]

# Supplementary materials

# Parameters of MRIs

All of MRIs were acquired using a MAGNETOM Prisma 3T MRI scanner (Siemens, Erlangen, Germany). We acquired all MRI data within 72 hours before tumor resection.

Parameters of the T1-3D image with gadolinium contrast enhancement were: 1) flip angle (FA) = 8°; 2) repetition time (TR) = 2300 ms; 3) echo time (TE) = 2.3 ms; 4) field of view (FOV) = 240 × 240 mm^2^; 5) voxel size = 1.0 × 1.0 × 1.0 mm^3^; and 6) slice number: 192.

Moreover, parameters of T2 fluid attenuated inversion recovery were: 1) FA = 150°; 2) TR = 5000 ms; 3) TE = 105 ms; 4) FOV = 240 × 240 mm^2^; 5) voxel size = 0.5 × 0.5 × 3 mm^3^; and 6) slice number = 33.

Additionally, parameters of resting state functional MRI (rs-fMRI) were: 1) FA = 75°; 2) TR = 2000 ms; 3) TE = 30 ms; 4) FOV = 220 × 220 mm^2^; 5) voxel size = 3.0 × 3.0 × 5.0 mm^3^; 6) slice number = 30; and 7) acquisition duration = 8 minutes. All patients were asked to stare on a white cross on the monitor without thinking anything during scanning).

# Parameters of Preprocessing

Parameters of preprocessing were as follows: 1) transformation (from the format of Digital Imaging and Communications in Medicine to the format of Neuroimaging Informatics Technology Initiative); 2) removing first five images; 3) Slice timing; 4) head motion correction; 5) spatial normalization: normalizing to echo-planar imaging template[^1^](#_ENREF_1); 6) Smoothing (full width half maximum = 4 mm); 7) temporal detrending (linear detrending); 8) regressing out covariance: white matter signal: with WMMask_3 mm, cerebrospinal fluid signal: with CSFMask_3 mm, and head motion: Friston - 24 parameters); 9) temporal filtering: 0.01–0.1 Hz; and 10) scrubbing: interpolation strategy: linear interpolation, FD threshold = 0.5, previous time point number = 1, and subsequent time point number = 2.

# Sensorimotor template selection

We extracted the sensorimotor network from the brain atlas (brant 274). Due to the cingulated cortices were related to motor control, we added the cingulated cortices in our sensorimotor template.[^2^](#_ENREF_2) Additionally, the tumor occupation would induce bias during normalization and affect the functional connectivity calculation. Hence, we excluded the regions which were invaded by glioma directly. The regions in the sensorimotor template were shown as follows:

**Table. Montreal Neurological Institute (MNI) locations of 40 nodes in the sensorimotor template**

| **Regions of interesting** | **Modified Cyto-architectonic** | **Left**  **hemisphere** | | | **Right hemisphere** | | |
| --- | --- | --- | --- | --- | --- | --- | --- |
|  |  | X | Y | Z | X | Y | Z |
| A6m | medial BA 6 | -6 | -5 | 58 | 7 | 4 | 60 |
| A4hf | BA 4 (head and face region) | -49 | -8 | 39 | 55 | -2 | 33 |
| A6cdl | caudal dorsolateral BA 6 | -32 | -9 | 58 | 33 | -7 | 57 |
| A4ul | BA 4 (upper limb region) | -26 | -25 | 63 | 34 | -19 | 59 |
| A4t | BA 4 (trunk region) | -13 | -20 | 73 | 42 | 31 | -9 |
| A4tl | BA 4 (tongue and larynx region) | -52 | 0 | 8 | 54 | 4 | 9 |
| A6cvl | caudal ventrolateral BA 6 | -49 | 5 | 30 | 51 | 7 | 30 |
| A1/2/3ulhf | BA 1/2/3 (upper limb, head and face region) | -50 | -16 | 43 | 50 | -14 | 44 |
| A1/2/3tonIa | BA 1/2/3 (tongue and larynx region) | -56 | -14 | 16 | 56 | -10 | 15 |
| A2 | BA 2 | -46 | -30 | 50 | 48 | -24 | 48 |
| A1/2/3tru | BA 1/2/3 (trunk region) | -21 | -35 | 68 | 20 | -33 | 69 |
| A23d | dorsal BA 23 | -4 | -39 | 31 | 4 | 37 | 32 |
| A4ll | BA 4 (lower limb region) | -4 | -23 | 61 | 5 | -21 | 61 |
| A1/2/3ll | BA 1/2/3 (lower limb region) | -8 | -38 | 58 | 10 | -34 | 54 |
| A23v | ventral BA 23 | -8 | -47 | 10 | 9 | -44 | 11 |
| A24cd | caudodorsal BA 24 | -5 | 7 | 37 | 4 | 6 | 38 |
| A23c | caudal BA 23 | -7 | -23 | 41 | 6 | -20 | 40 |
| A32sg | subgenual BA 32 | -4 | 39 | -2 | 5 | 41 | 6 |
| mPMtha | pre-motor thalamus | -18 | -13 | 3 | 12 | -14 | 1 |
| Stha | sensory thalamus | -18 | -23 | 4 | 18 | -22 | 3 |

*****BA = Brodmann area.

# Supplementary Tables

| **Table s1. Global properties in patients with different molecular diagnoses and different history of preoperative epilepsy (left hemispheric glioma)** | | | | | | | |
| --- | --- | --- | --- | --- | --- | --- | --- |
| **Node** | **Grp-E**  **(mean ± SEM)** | **Grp-nE**  **(mean ± SEM)** | **Grp-GnE**  **(mean ± SEM)** | **One-way ANOVA**  **(*p* value)** | **Post-hoc analysis with Sidak correction**  **(*p* value)** | | |
|  |  |  |  |  | **Grp-E VS**  **Grp-nE** | **Grp-E VS**  **Grp-GnE** | **Grp-nE VS**  **Grp-GnE** |
| Clustering coefficient | 0.081 ± 0.005 | 0.095 ± 0.002 | 0.093 ± 0.005 | 0.0461 | 0.0547 | 0.2345 | 0.9977 |
| Fault tolerance | 2.371 ± 0.108 | 2.697 ± 0.040 | 2.748 ± 0.060 | 0.0027 | 0.0071 | 0.0153 | 0.9708 |
| Global efficiency | 0.334 ± 0.019 | 0.277 ± 0.005 | 0.278 ± 0.013 | 0.0041 | 0.0057 | 0.0452 | > 0.9999 |
| Local efficiency | 0.331 ± 0.019 | 0.275 ± 0.005 | 0.276 ± 0.013 | 0.0039 | 0.0056 | 0.0432 | > 0.9999 |
| Shortest path length | 3.880 ± 0.143 | 4.381 ± 0.066 | 4.456 ± 0.139 | 0.0014 | 0.0041 | 0.0099 | 0.9693 |
| Transitivity | 0.021 ± 0.001 | 0.023 ± 0.001 | 0.022 ± 0.001 | 0.1646 | - | - | - |
| Vulnerability | 0.060 ± 0.004 | 0.060 ± 0.003 | 0.061 ± 0.005 | 0.9517 | - | - | - |
| * SEM, standard error mean. Grp-E, the group of patients with epilepsy and IDH mutation; Grp-nE, the group of patients with non-epilepsy and IDH mutation; Grp-GnE, the group of patients with non-epilepsy, glioblastoma (grade 4) and, IDH wildtype. | | | | | | | |

| **Table s2. Global properties in patients with different molecular diagnoses and different history of preoperative epilepsy (right hemispheric glioma)** | | | | | | | |
| --- | --- | --- | --- | --- | --- | --- | --- |
| **Node** | **Grp-E**  **(mean ± SEM)** | **Grp-nE**  **(mean ± SEM)** | **Grp-GnE**  **(mean ± SEM)** | **One-way ANOVA**  **(*p* value)** | **Post-hoc analysis with Sidak correction**  **(*p* value)** | | |
|  |  |  |  |  | **Grp-E VS**  **Grp-nE** | **Grp-E VS**  **Grp-GnE** | **Grp-nE VS**  **Grp-GnE** |
| Clustering coefficient | 0.065 ± 0.007 | 0.094 ± 0.005 | 0.102 ± 0.004 | 0.0002 | 0.0027 | 0.0004 | 0.7841 |
| Fault tolerance | 2.047 ± 0.156 | 2.621 ± 0.100 | 2.743 ± 0.061 | 0.0006 | 0.0049 | 0.0016 | 0.8962 |
| Global efficiency | 0.396 ± 0.033 | 0.288 ± 0.014 | 0.274 ± 0.009 | 0.0013 | 0.0064 | 0.0047 | 0.9765 |
| Local efficiency | 0.393 ± 0.033 | 0.286 ± 0.014 | 0.272 ± 0.009 | 0.0013 | 0.0063 | 0.0046 | 0.9761 |
| Shortest path length | 3.477 ± 0.213 | 4.308 ± 0.121 | 4.433 ± 0.119 | 0.0004 | 0.0025 | 0.0014 | 0.9517 |
| Transitivity | 0.017 ± 0.002 | 0.024 ± 0.002 | 0.026 ± 0.001 | 0.0007 | 0.0075 | 0.0014 | 0.8107 |
| Vulnerability | 0.057 ± 0.004 | 0.055 ± 0.004 | 0.062 ± 0.005 | 0.5664 | - | - | - |
| * SEM, standard error mean. Group-E, the group of patients with epilepsy and IDH mutation; Group-nE, the group of patients with non-epilepsy and IDH mutation; Group-GnE, the group of patients with non-epilepsy, glioblastoma (grade 4) and, IDH wildtype. | | | | | | | |

| **Table s3. Nodal Betweenness in patients with different molecular diagnoses and different history of preoperative epilepsy (left glioma)** | | | | | | | |
| --- | --- | --- | --- | --- | --- | --- | --- |
| **Node** | **Grp-E**  **(mean ± SEM)** | **Grp-nE**  **(mean ± SEM)** | **Grp-GnE**  **(mean ± SEM)** | **One-way ANOVA**  **(*p* value)** | **Post-hoc analysis with Sidak correction**  **(*p* value)** | | |
|  |  |  |  |  | **Grp-E VS**  **Grp-nE** | **Grp-E VS**  **Grp-GnE** | **Grp-nE VS**  **Grp-GnE** |
| A6m_L | 24.836 ± 4.315 | 15.997 ± 2.481 | 23.707 ± 4.501 | 0.1539 | - | - | - |
| A6m_R | 14.125 ± 2.709 | 11.963 ± 1.500 | 16.583 ± 2.427 | 0.4240 | - | - | - |
| A4hf_L | 21.377 ± 3.682 | 22.794 ± 2.740 | 25.504 ± 4.244 | 0.7695 | - | - | - |
| A4hf_R | 17.701 ± 2.031 | 20.767 ± 2.412 | 16.528 ± 3.834 | 0.5079 | - | - | - |
| A6cdl_L | 12.413 ± 2.241 | 23.244 ± 3.595 | 18.996 ± 4.608 | 0.0596 | - | - | - |
| A6cdl_R | 11.693 ± 1.997 | 23.916 ± 3.813 | 13.519 ± 2.908 | 0.0140 | 0.0171 | 0.9820 | 0.1513 |
| A4ul_L | 20.418 ± 3.428 | 23.940 ± 3.360 | 17.355 ± 3.043 | 0.4793 | - | - | - |
| A4ul_R | 20.933 ± 3.289 | 17.724 ± 3.118 | 13.818 ± 2.653 | 0.4184 | - | - | - |
| A4t_L | 19.335 ± 3.372 | 21.333 ± 4.015 | 32.017 ± 5.561 | 0.1688 | - | - | - |
| A4t_R | 23.436 ± 3.791 | 18.735 ± 2.289 | 16.784 ± 3.454 | 0.3727 | - | - | - |
| A4tl_L | 17.200 ± 2.744 | 15.123 ± 2.438 | 15.346 ± 4.814 | 0.8592 | - | - | - |
| A4tl_R | 17.776 ± 2.740 | 19.992 ± 2.758 | 18.692 ± 4.167 | 0.8569 | - | - | - |
| A6cvl_L | 17.480 ± 2.977 | 16.398 ± 2.800 | 18.970 ± 5.660 | 0.8942 | - | - | - |
| A6cvl_R | 20.119 ± 3.169 | 18.695 ± 2.491 | 19.989 ± 3.997 | 0.9305 | - | - | - |
| A1/2/3ulhf_L | 20.199 ± 3.256 | 20.359 ± 2.519 | 18.788 ± 2.712 | 0.9438 | - | - | - |
| A1/2/3ulhf_R | 25.240 ± 3.113 | 24.079 ± 2.972 | 19.737 ± 3.508 | 0.5786 | - | - | - |
| A1/2/3tonIa_L | 24.044 ± 3.289 | 15.822 ± 1.978 | 15.419 ± 4.596 | 0.0788 | - | - | - |
| A1/2/3tonIa_R | 20.881 ± 3.369 | 14.169 ± 1.954 | 19.996 ± 4.312 | 0.1963 | - | - | - |
| A2_L | 21.073 ± 3.227 | 24.732 ± 3.174 | 18.927 ± 3.398 | 0.5092 | - | - | - |
| A2_R | 24.329 ± 3.030 | 24.733 ± 3.391 | 23.083 ± 4.076 | 0.9576 | - | - | - |
| A1/2/3tru_L | 21.410 ± 2.949 | 23.730 ± 3.567 | 27.241 ± 5.054 | 0.6332 | - | - | - |
| A1/2/3tru_R | 20.582 ± 3.087 | 23.165 ± 2.748 | 16.688 ± 2.608 | 0.4051 | - | - | - |
| A23d_L | 23.571 ± 3.007 | 20.967 ± 2.562 | 22.517 ± 3.752 | 0.8017 | - | - | - |
| A23d_R | 23.795 ± 2.832 | 25.101 ± 2.479 | 25.494 ± 3.637 | 0.9169 | - | - | - |
| * SEM, standard error mean. Grp-E, the group of patients with epilepsy and IDH mutation; Grp-nE, the group of patients with non-epilepsy and IDH mutation; Grp-GnE, the group of patients with non-epilepsy, glioblastoma (grade 4) and, IDH wildtype. L, left hemispheric node; R, right hemispheric node. | | | | | | | |
| **Continue Table s3** | | | | | | | |
| **Node** | **Grp-E**  **(mean ± SEM)** | **Grp-nE**  **(mean ± SEM)** | **Grp-GnE**  **(mean ± SEM)** | **One-way ANOVA**  **(*p* value)** | **Post-hoc analysis with Sidak correction**  **(*p* value)** | | |
|  |  |  |  |  | **Grp-E VS**  **Grp-nE** | **Grp-E VS**  **Grp-GnE** | **Grp-nE VS**  **Grp-GnE** |
| A4ll_L | 12.033 ± 2.215 | 13.187 ± 2.188 | 18.177 ± 4.695 | 0.3696 | - | - | - |
| A4ll_R | 9.990 ± 1.851 | 13.474 ± 2.188 | 21.408 ± 5.218 | 0.0393 | 0.6889 | 0.0334 | 0.1914 |
| A1/2/3ll_L | 19.110 ± 2.425 | 15.167 ± 1.994 | 10.618 ± 1.941 | 0.0753 | - | - | - |
| A1/2/3ll_R | 24.655 ± 3.544 | 22.808 ± 2.813 | 29.218 ± 7.238 | 0.6007 | - | - | - |
| A23v_L | 22.857 ± 3.413 | 26.472 ± 3.050 | 25.688 ± 5.593 | 0.7466 | - | - | - |
| A23v_R | 21.135 ± 3.318 | 14.689 ± 2.121 | 17.462 ± 4.507 | 0.2795 | - | - | - |
| A24cd_L | 24.500 ± 3.284 | 25.944 ± 3.391 | 20.679 ± 3.837 | 0.6561 | - | - | - |
| A24cd_R | 23.855 ± 3.206 | 27.783 ± 3.327 | 30.628 ± 5.407 | 0.5098 | - | - | - |
| A23c_L | 20.033 ± 2.675 | 17.784 ± 2.466 | 17.265 ± 4.221 | 0.7870 | - | - | - |
| A23c_R | 20.392 ± 2.505 | 20.114 ± 2.777 | 25.094 ± 7.434 | 0.6674 | - | - | - |
| A32sg_L | 18.375 ± 2.427 | 20.639 ± 2.837 | 16.402 ± 2.344 | 0.6110 | - | - | - |
| A32sg_R | 20.678 ± 2.585 | 23.492 ± 2.274 | 19.378 ± 2.378 | 0.5274 | - | - | - |
| mPMtha_L | 14.447 ± 3.418 | 12.518 ± 2.115 | 13.451 ± 3.480 | 0.8833 | - | - | - |
| mPMtha_R | 22.805 ± 4.725 | 20.267 ± 3.506 | 22.071 ± 4.806 | 0.9008 | - | - | - |
| Stha_L | 9.219 ± 1.691 | 20.827 ± 2.684 | 14.235 ± 2.805 | 0.0024 | 0.0017 | 0.5326 | 0.2765 |
| Stha_R | 19.156 ± 2.785 | 12.778 ± 2.149 | 18.575 ± 3.203 | 0.1466 | - | - | - |
| * SEM, standard error mean. Grp-E, the group of patients with epilepsy and IDH mutation; Grp-nE, the group of patients with non-epilepsy and IDH mutation; Grp-GnE, the group of patients with non-epilepsy, glioblastoma (grade 4) and, IDH wildtype. L, left hemispheric node; R, right hemispheric node. | | | | | | | |

| **Table s4. Nodal Betweenness in patients with different molecular diagnoses and different history of preoperative epilepsy (right glioma)** | | | | | | | |
| --- | --- | --- | --- | --- | --- | --- | --- |
| **Node** | **Grp-E**  **(mean ± SEM)** | **Grp-nE**  **(mean ± SEM)** | **Grp-GnE**  **(mean ± SEM)** | **One-way ANOVA**  **(*p* value)** | **Post-hoc analysis with Sidak correction**  **(*p* value)** | | |
|  |  |  |  |  | **Grp-E VS**  **Grp-nE** | **Grp-E VS**  **Grp-GnE** | **Grp-nE VS**  **Grp-GnE** |
| A6m_L | 13.825 ± 2.329 | 15.490 ± 2.980 | 17.981 ± 4.159 | 0.6638 | - | - | - |
| A6m_R | 28.503 ± 4.003 | 20.316 ± 2.830 | 16.409 ± 3.178 | 0.0607 | - | - | - |
| A4hf_L | 21.614 ± 2.433 | 23.421 ± 3.457 | 24.611 ± 7.529 | 0.8926 | - | - | - |
| A4hf_R | 14.641 ± 2.880 | 13.960 ± 3.390 | 14.582 ± 3.401 | 0.9864 | - | - | - |
| A6cdl_L | 10.947 ± 1.656 | 21.636 ± 3.296 | 15.375 ± 2.084 | 0.0109 | 0.0084 | 0.5644 | 0.2854 |
| A6cdl_R | 14.787 ± 3.645 | 18.713 ± 3.669 | 24.310 ± 5.822 | 0.3317 | - | - | - |
| A4ul_L | 21.724 ± 3.899 | 21.369 ± 4.703 | 14.252 ± 3.155 | 0.4325 | - | - | - |
| A4ul_R | 22.460 ± 4.622 | 20.771 ± 3.884 | 25.697 ± 4.910 | 0.7721 | - | - | - |
| A4t_L | 24.502 ± 4.200 | 19.544 ± 3.495 | 17.995 ± 4.230 | 0.5041 | - | - | - |
| A4t_R | 26.944 ± 5.112 | 19.285 ± 3.086 | 18.642 ± 3.000 | 0.2947 | - | - | - |
| A4tl_L | 18.893 ± 2.913 | 21.823 ± 4.870 | 10.272 ± 1.865 | 0.1190 | - | - | - |
| A4tl_R | 19.675 ± 3.943 | 16.902 ± 3.284 | 15.139 ± 3.099 | 0.6901 | - | - | - |
| A6cvl_L | 14.870 ± 2.601 | 15.869 ± 4.435 | 15.276 ± 3.345 | 0.9794 | - | - | - |
| A6cvl_R | 18.369 ± 3.298 | 15.316 ± 3.005 | 19.397 ± 3.816 | 0.6920 | - | - | - |
| A1/2/3ulhf_L | 23.619 ± 5.058 | 23.357 ± 3.035 | 33.411 ± 4.840 | 0.2625 | - | - | - |
| A1/2/3ulhf_R | 32.088 ± 5.596 | 13.486 ± 3.329 | 31.750 ± 6.795 | 0.0224 | 0.0368 | > 0.9999 | 0.0776 |
| A1/2/3tonIa_L | 21.457 ± 4.003 | 19.114 ± 4.661 | 16.440 ± 4.700 | 0.7549 | - | - | - |
| A1/2/3tonIa_R | 13.337 ± 3.251 | 27.617 ± 5.259 | 25.608 ± 7.688 | 0.1097 | - | - | - |
| A2_L | 18.816 ± 3.248 | 22.385 ± 3.908 | 16.851 ± 3.459 | 0.5819 | - | - | - |
| A2_R | 24.468 ± 4.170 | 16.026 ± 2.710 | 20.175 ± 4.930 | 0.3024 | - | - | - |
| A1/2/3tru_L | 31.966 ± 5.055 | 23.698 ± 3.233 | 20.457 ± 4.585 | 0.1888 | - | - | - |
| A1/2/3tru_R | 18.194 ± 4.186 | 19.470 ± 3.607 | 30.147 ± 7.083 | 0.2187 | - | - | - |
| A23d_L | 21.054 ± 3.511 | 21.201 ± 2.791 | 25.974 ± 6.135 | 0.6709 | - | - | - |
| A23d_R | 18.684 ± 3.178 | 24.993 ± 3.290 | 22.779 ± 3.087 | 0.3639 | - | - | - |
| * SEM, standard error mean. Grp-E, the group of patients with epilepsy and IDH mutation; Grp-nE, the group of patients with non-epilepsy and IDH mutation; Grp-GnE, the group of patients with non-epilepsy, glioblastoma (grade 4) and, IDH wildtype. L, left hemispheric node; R, right hemispheric node. | | | | | | | |
| **Continue Table s4** | | | | | | | |
| **Node** | **Grp-E**  **(mean ± SEM)** | **Grp-nE**  **(mean ± SEM)** | **Grp-GnE**  **(mean ± SEM)** | **One-way ANOVA**  **(*p* value)** | **Post-hoc analysis with Sidak correction**  **(*p* value)** | | |
|  |  |  |  |  | **Grp-E VS**  **Grp-nE** | **Grp-E VS**  **Grp-GnE** | **Grp-nE VS**  **Grp-GnE** |
| A4ll_L | 16.308 ± 2.953 | 8.594 ± 2.020 | 11.019 ± 2.867 | 0.1080 | - | - | - |
| A4ll_R | 12.564 ± 2.633 | 10.301 ± 2.561 | 12.447 ± 3.185 | 0.8103 | - | - | - |
| A1/2/3ll_L | 14.644 ± 2.602 | 19.701 ± 3.819 | 27.627 ± 4.482 | 0.0593 | - | - | - |
| A1/2/3ll_R | 21.462 ± 2.692 | 31.699 ± 5.730 | 18.700 ± 4.271 | 0.1128 | - | - | - |
| A23v_L | 16.284 ± 2.924 | 19.281 ± 3.023 | 22.127 ± 4.599 | 0.5168 | - | - | - |
| A23v_R | 16.228 ± 3.145 | 20.698 ± 3.777 | 23.983 ± 4.596 | 0.3779 | - | - | - |
| A24cd_L | 22.931 ± 3.563 | 30.927 ± 5.391 | 20.976 ± 6.140 | 0.3480 | - | - | - |
| A24cd_R | 18.542 ± 2.991 | 26.100 ± 4.524 | 27.743 ± 5.648 | 0.2793 | - | - | - |
| A23c_L | 17.777 ± 2.965 | 13.594 ± 2.785 | 25.550 ± 5.779 | 0.1125 | - | - | - |
| A23c_R | 27.090 ± 4.421 | 17.180 ± 2.815 | 16.704 ± 3.170 | 0.0896 | - | - | - |
| A32sg_L | 18.825 ± 3.248 | 17.351 ± 3.374 | 17.950 ± 3.280 | 0.9494 | - | - | - |
| A32sg_R | 23.373 ± 3.461 | 20.189 ± 2.735 | 25.380 ± 8.166 | 0.7548 | - | - | - |
| mPMtha_L | 9.216 ± 1.725 | 11.757 ± 1.521 | 7.026 ± 2.421 | 0.2449 | - | - | - |
| mPMtha_R | 14.490 ± 2.218 | 12.302 ± 2.780 | 12.423 ± 2.876 | 0.7964 | - | - | - |
| Stha_L | 14.186 ± 3.013 | 15.853 ± 3.093 | 13.481 ± 2.771 | 0.8641 | - | - | - |
| Stha_R | 18.675 ± 3.582 | 21.028 ± 3.223 | 13.046 ± 3.504 | 0.3246 | - | - | - |
| * SEM, standard error mean. Grp-E, the group of patients with epilepsy and IDH mutation; Grp-nE, the group of patients with non-epilepsy and IDH mutation; Grp-GnE, the group of patients with non-epilepsy, glioblastoma (grade 4) and, IDH wildtype. L, left hemispheric node; R, right hemispheric node. | | | | | | | |

| **Table s5. Nodal Degree Centrality in patients with different molecular diagnoses and different history of preoperative epilepsy (left glioma)** | | | | | | | |
| --- | --- | --- | --- | --- | --- | --- | --- |
| **Node** | **Grp-E**  **(mean ± SEM)** | **Grp-nE**  **(mean ± SEM)** | **Grp-GnE**  **(mean ± SEM)** | **One-way ANOVA**  **(*p* value)** | **Post-hoc analysis with Sidak correction**  **(*p* value)** | | |
|  |  |  |  |  | **Grp-E VS**  **Grp-nE** | **Grp-E VS**  **Grp-GnE** | **Grp-nE VS**  **Grp-GnE** |
| A6m_L | 3.737 ± 0.312 | 2.923 ± 0.155 | 3.479 ± 0.365 | 0.0590 | - | - | - |
| A6m_R | 3.462 ± 0.383 | 2.905 ± 0.139 | 3.229 ± 0.249 | 0.3169 | - | - | - |
| A4hf_L | 3.631 ± 0.205 | 3.369 ± 0.140 | 3.467 ± 0.256 | 0.5715 | - | - | - |
| A4hf_R | 3.629 ± 0.329 | 3.185 ± 0.210 | 2.873 ± 0.207 | 0.2135 | - | - | - |
| A6cdl_L | 3.102 ± 0.217 | 3.127 ± 0.169 | 3.251 ± 0.315 | 0.9120 | - | - | - |
| A6cdl_R | 3.250 ± 0.275 | 3.359 ± 0.207 | 2.954 ± 0.412 | 0.6546 | - | - | - |
| A4ul_L | 3.345 ± 0.290 | 3.310 ± 0.155 | 2.978 ± 0.258 | 0.6121 | - | - | - |
| A4ul_R | 3.606 ± 0.274 | 3.017 ± 0.145 | 3.163 ± 0.398 | 0.1830 | - | - | - |
| A4t_L | 3.367 ± 0.242 | 3.175 ± 0.190 | 3.949 ± 0.482 | 0.1964 | - | - | - |
| A4t_R | 3.755 ± 0.344 | 3.177 ± 0.224 | 3.299 ± 0.436 | 0.3588 | - | - | - |
| A4tl_L | 3.517 ± 0.322 | 2.893 ± 0.153 | 2.984 ± 0.284 | 0.1597 | - | - | - |
| A4tl_R | 3.645 ± 0.288 | 3.194 ± 0.142 | 3.488 ± 0.580 | 0.4817 | - | - | - |
| A6cvl_L | 3.320 ± 0.238 | 2.861 ± 0.134 | 3.289 ± 0.565 | 0.3558 | - | - | - |
| A6cvl_R | 3.554 ± 0.309 | 3.124 ± 0.202 | 3.531 ± 0.452 | 0.4782 | - | - | - |
| A1/2/3ulhf_L | 3.428 ± 0.178 | 3.285 ± 0.136 | 3.391 ± 0.255 | 0.8157 | - | - | - |
| A1/2/3ulhf_R | 3.812 ± 0.264 | 3.439 ± 0.148 | 3.134 ± 0.153 | 0.1309 | - | - | - |
| A1/2/3tonIa_L | 4.038 ± 0.391 | 3.163 ± 0.191 | 3.024 ± 0.302 | 0.0517 | - | - | - |
| A1/2/3tonIa_R | 3.540 ± 0.207 | 2.904 ± 0.115 | 3.410 ± 0.489 | 0.0885 | - | - | - |
| A2_L | 3.977 ± 0.410 | 3.386 ± 0.174 | 3.098 ± 0.241 | 0.1642 | - | - | - |
| A2_R | 3.781 ± 0.274 | 3.420 ± 0.177 | 3.198 ± 0.208 | 0.2678 | - | - | - |
| A1/2/3tru_L | 3.808 ± 0.398 | 3.185 ± 0.167 | 3.799 ± 0.468 | 0.2798 | - | - | - |
| A1/2/3tru_R | 3.932 ± 0.425 | 3.237 ± 0.193 | 3.443 ± 0.500 | 0.3182 | - | - | - |
| A23d_L | 3.857 ± 0.300 | 3.071 ± 0.148 | 3.581 ± 0.357 | 0.0602 | - | - | - |
| A23d_R | 3.828 ± 0.237 | 3.420 ± 0.163 | 3.802 ± 0.361 | 0.3454 | - | - | - |
| * SEM, standard error mean. Grp-E, the group of patients with epilepsy and IDH mutation; Grp-nE, the group of patients with non-epilepsy and IDH mutation; Grp-GnE, the group of patients with non-epilepsy, glioblastoma (grade 4) and, IDH wildtype. L, left hemispheric node; R, right hemispheric node. | | | | | | | |
| **Continue Table s5** | | | | | | | |
| **Node** | **Grp-E**  **(mean ± SEM)** | **Grp-nE**  **(mean ± SEM)** | **Grp-GnE**  **(mean ± SEM)** | **One-way ANOVA**  **(*p* value)** | **Post-hoc analysis with Sidak correction**  **(*p* value)** | | |
|  |  |  |  |  | **Grp-E VS**  **Grp-nE** | **Grp-E VS**  **Grp-GnE** | **Grp-nE VS**  **Grp-GnE** |
| A4ll_L | 3.138 ± 0.251 | 2.917 ± 0.187 | 3.253 ± 0.454 | 0.6801 | - | - | - |
| A4ll_R | 3.341 ± 0.388 | 3.000 ± 0.149 | 3.228 ± 0.598 | 0.7365 | - | - | - |
| A1/2/3ll_L | 3.791 ± 0.365 | 2.947 ± 0.132 | 2.767 ± 0.208 | 0.0215 | 0.0495 | 0.0623 | 0.9654 |
| A1/2/3ll_R | 3.719 ± 0.334 | 3.249 ± 0.118 | 3.741 ± 0.608 | 0.4274 | - | - | - |
| A23v_L | 3.830 ± 0.373 | 3.505 ± 0.152 | 3.735 ± 0.310 | 0.6744 | - | - | - |
| A23v_R | 3.620 ± 0.358 | 2.829 ± 0.137 | 2.933 ± 0.196 | 0.0602 | - | - | - |
| A24cd_L | 3.779 ± 0.247 | 3.401 ± 0.171 | 3.319 ± 0.254 | 0.3219 | - | - | - |
| A24cd_R | 3.979 ± 0.350 | 3.562 ± 0.241 | 3.524 ± 0.195 | 0.4939 | - | - | - |
| A23c_L | 3.798 ± 0.412 | 3.080 ± 0.150 | 3.041 ± 0.204 | 0.1339 | - | - | - |
| A23c_R | 3.799 ± 0.314 | 3.013 ± 0.150 | 3.333 ± 0.234 | 0.0543 | - | - | - |
| A32sg_L | 3.390 ± 0.257 | 3.083 ± 0.156 | 2.607 ± 0.146 | 0.0806 | - | - | - |
| A32sg_R | 3.725 ± 0.340 | 3.348 ± 0.163 | 3.085 ± 0.244 | 0.3043 | - | - | - |
| mPMtha_L | 3.080 ± 0.265 | 2.700 ± 0.134 | 3.358 ± 0.606 | 0.3113 | - | - | - |
| mPMtha_R | 3.834 ± 0.386 | 3.148 ± 0.169 | 3.823 ± 0.602 | 0.2499 | - | - | - |
| Stha_L | 2.838 ± 0.232 | 3.243 ± 0.149 | 2.917 ± 0.203 | 0.2719 | - | - | - |
| Stha_R | 3.987 ± 0.349 | 2.871 ± 0.183 | 3.576 ± 0.505 | 0.0262 | 0.0230 | 0.8132 | 0.4172 |
| * SEM, standard error mean. Grp-E, the group of patients with epilepsy and IDH mutation; Grp-nE, the group of patients with non-epilepsy and IDH mutation; Grp-GnE, the group of patients with non-epilepsy, glioblastoma (grade 4) and, IDH wildtype. L, left hemispheric node; R, right hemispheric node. | | | | | | | |

| **Table s6. Nodal Degree Centrality in patients with different molecular diagnoses and different history of preoperative epilepsy (right glioma)** | | | | | | | |
| --- | --- | --- | --- | --- | --- | --- | --- |
| **Node** | **Grp-E**  **(mean ± SEM)** | **Grp-nE**  **(mean ± SEM)** | **Grp-GnE**  **(mean ± SEM)** | **One-way ANOVA**  **(*p* value)** | **Post-hoc analysis with Sidak correction**  **(*p* value)** | | |
|  |  |  |  |  | **Grp-E VS**  **Grp-nE** | **Grp-E VS**  **Grp-GnE** | **Grp-nE VS**  **Grp-GnE** |
| A6m_L | 4.562 ± 0.726 | 3.355 ± 0.462 | 3.331 ± 0.342 | 0.5636 | - | - | - |
| A6m_R | 4.859 ± 0.715 | 3.453 ± 0.387 | 3.356 ± 0.377 | 0.6363 | - | - | - |
| A4hf_L | 4.844 ± 0.768 | 4.386 ± 0.677 | 3.843 ± 0.497 | 0.6199 | - | - | - |
| A4hf_R | 4.04 ± 0.668 | 3.450 ± 0.433 | 3.241 ± 0.370 | 0.9173 | - | - | - |
| A6cdl_L | 3.418 ± 0.354 | 4.389 ± 0.838 | 3.260 ± 0.158 | 0.3049 | - | - | - |
| A6cdl_R | 3.294 ± 0.335 | 3.589 ± 0.399 | 3.568 ± 0.422 | 0.5134 | - | - | - |
| A4ul_L | 3.804 ± 0.523 | 3.814 ± 0.655 | 3.551 ± 0.381 | 0.2775 | - | - | - |
| A4ul_R | 3.962 ± 0.500 | 4.074 ± 0.743 | 3.871 ± 0.354 | 0.1534 | - | - | - |
| A4t_L | 4.204 ± 0.404 | 4.475 ± 0.765 | 3.130 ± 0.314 | 0.4494 | - | - | - |
| A4t_R | 4.960 ± 0.824 | 4.367 ± 0.746 | 3.325 ± 0.210 | 0.5786 | - | - | - |
| A4tl_L | 4.434 ± 0.585 | 4.360 ± 0.794 | 2.743 ± 0.226 | 0.4849 | - | - | - |
| A4tl_R | 4.386 ± 0.749 | 3.726 ± 0.582 | 2.894 ± 0.337 | 0.3581 | - | - | - |
| A6cvl_L | 4.281 ± 0.553 | 3.281 ± 0.383 | 3.196 ± 0.388 | 0.9493 | - | - | - |
| A6cvl_R | 3.996 ± 0.482 | 3.098 ± 0.310 | 3.766 ± 0.462 | 0.6898 | - | - | - |
| A1/2/3ulhf_L | 4.268 ± 0.543 | 4.289 ± 0.767 | 4.322 ± 0.496 | 0.8183 | - | - | - |
| A1/2/3ulhf_R | 4.919 ± 0.681 | 3.808 ± 0.619 | 4.128 ± 0.492 | 0.5203 | - | - | - |
| A1/2/3tonIa_L | 4.347 ± 0.658 | 3.888 ± 0.581 | 3.112 ± 0.294 | 0.4242 | - | - | - |
| A1/2/3tonIa_R | 3.598 ± 0.408 | 4.285 ± 0.693 | 3.451 ± 0.314 | 0.4444 | - | - | - |
| A2_L | 3.450 ± 0.333 | 3.734 ± 0.449 | 3.360 ± 0.402 | 0.1642 | - | - | - |
| A2_R | 4.360 ± 0.717 | 3.953 ± 0.796 | 3.467 ± 0.491 | 0.9808 | - | - | - |
| A1/2/3tru_L | 5.252 ± 0.689 | 4.372 ± 0.710 | 3.349 ± 0.267 | 0.7818 | - | - | - |
| A1/2/3tru_R | 3.870 ± 0.471 | 3.874 ± 0.595 | 3.306 ± 0.228 | 0.6435 | - | - | - |
| A23d_L | 3.735 ± 0.308 | 4.191 ± 0.841 | 3.543 ± 0.353 | 0.5955 | - | - | - |
| A23d_R | 4.177 ± 0.588 | 4.132 ± 0.606 | 3.313 ± 0.223 | 0.7624 | - | - | - |
| * SEM, standard error mean. Grp-E, the group of patients with epilepsy and IDH mutation; Grp-nE, the group of patients with non-epilepsy and IDH mutation; Grp-GnE, the group of patients with non-epilepsy, glioblastoma (grade 4) and, IDH wildtype. L, left hemispheric node; R, right hemispheric node. | | | | | | | |
| **Continue Table s6** | | | | | | | |
| **Node** | **Grp-E**  **(mean ± SEM)** | **Grp-nE**  **(mean ± SEM)** | **Grp-GnE**  **(mean ± SEM)** | **One-way ANOVA**  **(*p* value)** | **Post-hoc analysis with Sidak correction**  **(*p* value)** | | |
|  |  |  |  |  | **Grp-E VS**  **Grp-nE** | **Grp-E VS**  **Grp-GnE** | **Grp-nE VS**  **Grp-GnE** |
| A4ll_L | 3.789 ± 0.459 | 2.710 ± 0.448 | 2.944 ± 0.229 | 0.4950 | - | - | - |
| A4ll_R | 4.168 ± 0.577 | 3.075 ± 0.484 | 2.869 ± 0.247 | 0.9473 | - | - | - |
| A1/2/3ll_L | 4.315 ± 0.700 | 2.701 ± 0.188 | 3.346 ± 0.206 | 0.7296 | - | - | - |
| A1/2/3ll_R | 4.830 ± 0.813 | 4.044 ± 0.725 | 2.928 ± 0.257 | 0.6739 | - | - | - |
| A23v_L | 4.585 ± 0.771 | 4.005 ± 0.716 | 3.676 ± 0.389 | 0.2983 | - | - | - |
| A23v_R | 3.598 ± 0.390 | 3.015 ± 0.258 | 3.359 ± 0.301 | 0.5085 | - | - | - |
| A24cd_L | 4.039 ± 0.422 | 4.669 ± 0.820 | 3.072 ± 0.286 | 0.4677 | - | - | - |
| A24cd_R | 3.909 ± 0.330 | 4.492 ± 0.801 | 3.871 ± 0.518 | 0.4233 | - | - | - |
| A23c_L | 3.853 ± 0.515 | 3.651 ± 0.626 | 3.556 ± 0.540 | 0.4473 | - | - | - |
| A23c_R | 4.895 ± 0.662 | 3.574 ± 0.437 | 3.160 ± 0.405 | 0.8072 | - | - | - |
| A32sg_L | 4.014 ± 0.668 | 3.393 ± 0.344 | 3.207 ± 0.225 | 0.1362 | - | - | - |
| A32sg_R | 4.413 ± 0.497 | 3.702 ± 0.724 | 3.627 ± 0.402 | 0.7908 | - | - | - |
| mPMtha_L | 3.727 ± 0.475 | 3.926 ± 0.764 | 2.614 ± 0.278 | 0.2708 | - | - | - |
| mPMtha_R | 3.825 ± 0.378 | 3.019 ± 0.466 | 2.997 ± 0.402 | 0.8463 | - | - | - |
| Stha_L | 4.148 ± 0.764 | 2.937 ± 0.298 | 3.197 ± 0.375 | 0.4202 | - | - | - |
| Stha_R | 3.904 ± 0.489 | 3.129 ± 0.281 | 2.895 ± 0.232 | 0.4951 | - | - | - |
| * SEM, standard error mean. Grp-E, the group of patients with epilepsy and IDH mutation; Grp-nE, the group of patients with non-epilepsy and IDH mutation; Grp-GnE, the group of patients with non-epilepsy, glioblastoma (grade 4) and, IDH wildtype. L, left hemispheric node; R, right hemispheric node. | | | | | | | |

| **Table s7. Nodal Clustering Coefficient in patients with different molecular diagnoses and different history of preoperative epilepsy (left glioma)** | | | | | | | |
| --- | --- | --- | --- | --- | --- | --- | --- |
| **Node** | **Grp-E**  **(mean ± SEM)** | **Grp-nE**  **(mean ± SEM)** | **Grp-GnE**  **(mean ± SEM)** | **One-way ANOVA**  **(*p* value)** | **Post-hoc analysis with Sidak correction**  **(*p* value)** | | |
|  |  |  |  |  | **Grp-E VS**  **Grp-nE** | **Grp-E VS**  **Grp-GnE** | **Grp-nE VS**  **Grp-GnE** |
| A6m_L | 0.024 ± 0.002 | 0.026 ± 0.002 | 0.026 ± 0.003 | 0.8082 | - | - | - |
| A6m_R | 0.027 ± 0.002 | 0.028 ± 0.002 | 0.026 ± 0.002 | 0.7033 | - | - | - |
| A4hf_L | 0.025 ± 0.002 | 0.027 ± 0.002 | 0.032 ± 0.004 | 0.1871 | - | - | - |
| A4hf_R | 0.027 ± 0.002 | 0.028 ± 0.002 | 0.029 ± 0.004 | 0.9005 | - | - | - |
| A6cdl_L | 0.026 ± 0.002 | 0.028 ± 0.002 | 0.024 ± 0.003 | 0.5842 | - | - | - |
| A6cdl_R | 0.025 ± 0.002 | 0.026 ± 0.002 | 0.031 ± 0.005 | 0.4266 | - | - | - |
| A4ul_L | 0.025 ± 0.002 | 0.025 ± 0.002 | 0.027 ± 0.003 | 0.7866 | - | - | - |
| A4ul_R | 0.025 ± 0.002 | 0.029 ± 0.002 | 0.028 ± 0.004 | 0.6451 | - | - | - |
| A4t_L | 0.025 ± 0.002 | 0.026 ± 0.002 | 0.025 ± 0.003 | 0.9497 | - | - | - |
| A4t_R | 0.026 ± 0.003 | 0.025 ± 0.002 | 0.025 ± 0.004 | 0.9834 | - | - | - |
| A4tl_L | 0.023 ± 0.002 | 0.029 ± 0.002 | 0.022 ± 0.002 | 0.0577 | - | - | - |
| A4tl_R | 0.026 ± 0.002 | 0.027 ± 0.002 | 0.025 ± 0.003 | 0.8910 | - | - | - |
| A6cvl_L | 0.026 ± 0.002 | 0.024 ± 0.002 | 0.023 ± 0.004 | 0.8251 | - | - | - |
| A6cvl_R | 0.026 ± 0.002 | 0.030 ± 0.002 | 0.023 ± 0.004 | 0.2562 | - | - | - |
| A1/2/3ulhf_L | 0.028 ± 0.003 | 0.027 ± 0.002 | 0.033 ± 0.004 | 0.3472 | - | - | - |
| A1/2/3ulhf_R | 0.027 ± 0.003 | 0.027 ± 0.002 | 0.027 ± 0.003 | 0.9743 | - | - | - |
| A1/2/3tonIa_L | 0.027 ± 0.002 | 0.025 ± 0.002 | 0.026 ± 0.004 | 0.8838 | - | - | - |
| A1/2/3tonIa_R | 0.026 ± 0.002 | 0.026 ± 0.002 | 0.025 ± 0.003 | 0.9360 | - | - | - |
| A2_L | 0.026 ± 0.002 | 0.026 ± 0.002 | 0.024 ± 0.003 | 0.8720 | - | - | - |
| A2_R | 0.026 ± 0.002 | 0.027 ± 0.002 | 0.026 ± 0.003 | 0.8850 | - | - | - |
| A1/2/3tru_L | 0.026 ± 0.002 | 0.027 ± 0.002 | 0.028 ± 0.004 | 0.9298 | - | - | - |
| A1/2/3tru_R | 0.026 ± 0.002 | 0.027 ± 0.002 | 0.028 ± 0.003 | 0.8847 | - | - | - |
| A23d_L | 0.024 ± 0.003 | 0.030 ± 0.005 | 0.022 ± 0.004 | 0.3768 | - | - | - |
| A23d_R | 0.023 ± 0.002 | 0.026 ± 0.002 | 0.022 ± 0.003 | 0.4244 | - | - | - |
| * SEM, standard error mean. Grp-E, the group of patients with epilepsy and IDH mutation; Grp-nE, the group of patients with non-epilepsy and IDH mutation; Grp-GnE, the group of patients with non-epilepsy, glioblastoma (grade 4) and, IDH wildtype. L, left hemispheric node; R, right hemispheric node. | | | | | | | |
| **Continue Table s7** | | | | | | | |
| **Node** | **Grp-E**  **(mean ± SEM)** | **Grp-nE**  **(mean ± SEM)** | **Grp-GnE**  **(mean ± SEM)** | **One-way ANOVA**  **(*p* value)** | **Post-hoc analysis with Sidak correction**  **(*p* value)** | | |
|  |  |  |  |  | **Grp-E VS**  **Grp-nE** | **Grp-E VS**  **Grp-GnE** | **Grp-nE VS**  **Grp-GnE** |
| A4ll_L | 0.021 ± 0.002 | 0.028 ± 0.003 | 0.024 ± 0.004 | 0.1353 | - | - | - |
| A4ll_R | 0.023 ± 0.002 | 0.026 ± 0.002 | 0.030 ± 0.004 | 0.1813 | - | - | - |
| A1/2/3ll_L | 0.024 ± 0.002 | 0.027 ± 0.002 | 0.024 ± 0.002 | 0.4377 | - | - | - |
| A1/2/3ll_R | 0.028 ± 0.002 | 0.029 ± 0.002 | 0.028 ± 0.005 | 0.9104 | - | - | - |
| A23v_L | 0.026 ± 0.002 | 0.025 ± 0.002 | 0.025 ± 0.003 | 0.9099 | - | - | - |
| A23v_R | 0.025 ± 0.002 | 0.030 ± 0.002 | 0.024 ± 0.004 | 0.3396 | - | - | - |
| A24cd_L | 0.024 ± 0.003 | 0.029 ± 0.002 | 0.025 ± 0.003 | 0.4110 | - | - | - |
| A24cd_R | 0.026 ± 0.003 | 0.029 ± 0.002 | 0.026 ± 0.003 | 0.5570 | - | - | - |
| A23c_L | 0.027 ± 0.002 | 0.028 ± 0.002 | 0.027 ± 0.003 | 0.9079 | - | - | - |
| A23c_R | 0.024 ± 0.002 | 0.029 ± 0.002 | 0.027 ± 0.003 | 0.2501 | - | - | - |
| A32sg_L | 0.023 ± 0.002 | 0.028 ± 0.002 | 0.026 ± 0.004 | 0.3284 | - | - | - |
| A32sg_R | 0.024 ± 0.002 | 0.029 ± 0.002 | 0.027 ± 0.003 | 0.3218 | - | - | - |
| mPMtha_L | 0.020 ± 0.002 | 0.026 ± 0.002 | 0.024 ± 0.003 | 0.0561 | - | - | - |
| mPMtha_R | 0.024 ± 0.002 | 0.029 ± 0.002 | 0.026 ± 0.004 | 0.3556 | - | - | - |
| Stha_L | 0.023 ± 0.002 | 0.024 ± 0.001 | 0.023 ± 0.003 | 0.7641 | - | - | - |
| Stha_R | 0.023 ± 0.002 | 0.033 ± 0.002 | 0.020 ± 0.003 | 0.0011 | 0.0071 | 0.8698 | 0.0048 |
| * SEM, standard error mean. Grp-E, the group of patients with epilepsy and IDH mutation; Grp-nE, the group of patients with non-epilepsy and IDH mutation; Grp-GnE, the group of patients with non-epilepsy, glioblastoma (grade 4) and, IDH wildtype. L, left hemispheric node; R, right hemispheric node. | | | | | | | |

| **Table s8. Nodal Clustering Coefficient in patients with different molecular diagnoses and different history of preoperative epilepsy (right glioma)** | | | | | | | |
| --- | --- | --- | --- | --- | --- | --- | --- |
| **Node** | **Grp-E**  **(mean ± SEM)** | **Grp-nE**  **(mean ± SEM)** | **Grp-GnE**  **(mean ± SEM)** | **One-way ANOVA**  **(*p* value)** | **Post-hoc analysis with Sidak correction**  **(*p* value)** | | |
|  |  |  |  |  | **Grp-E VS**  **Grp-nE** | **Grp-E VS**  **Grp-GnE** | **Grp-nE VS**  **Grp-GnE** |
| A6m_L | 0.024 ± 0.003 | 0.028 ± 0.003 | 0.030 ± 0.004 | 0.4918 | - | - | - |
| A6m_R | 0.025 ± 0.004 | 0.027 ± 0.003 | 0.034 ± 0.008 | 0.4987 | - | - | - |
| A4hf_L | 0.022 ± 0.003 | 0.025 ± 0.003 | 0.029 ± 0.005 | 0.3586 | - | - | - |
| A4hf_R | 0.023 ± 0.003 | 0.029 ± 0.003 | 0.030 ± 0.004 | 0.3165 | - | - | - |
| A6cdl_L | 0.026 ± 0.003 | 0.028 ± 0.003 | 0.025 ± 0.003 | 0.7463 | - | - | - |
| A6cdl_R | 0.024 ± 0.003 | 0.029 ± 0.003 | 0.024 ± 0.003 | 0.4476 | - | - | - |
| A4ul_L | 0.022 ± 0.002 | 0.029 ± 0.004 | 0.028 ± 0.004 | 0.2317 | - | - | - |
| A4ul_R | 0.020 ± 0.002 | 0.026 ± 0.003 | 0.025 ± 0.003 | 0.2586 | - | - | - |
| A4t_L | 0.021 ± 0.003 | 0.027 ± 0.003 | 0.029 ± 0.004 | 0.2442 | - | - | - |
| A4t_R | 0.024 ± 0.003 | 0.025 ± 0.003 | 0.025 ± 0.003 | 0.9082 | - | - | - |
| A4tl_L | 0.021 ± 0.002 | 0.025 ± 0.003 | 0.030 ± 0.004 | 0.1145 | - | - | - |
| A4tl_R | 0.022 ± 0.003 | 0.026 ± 0.003 | 0.024 ± 0.002 | 0.6105 | - | - | - |
| A6cvl_L | 0.021 ± 0.002 | 0.036 ± 0.007 | 0.030 ± 0.005 | 0.0747 | - | - | - |
| A6cvl_R | 0.021 ± 0.002 | 0.027 ± 0.003 | 0.024 ± 0.003 | 0.2918 | - | - | - |
| A1/2/3ulhf_L | 0.025 ± 0.003 | 0.032 ± 0.003 | 0.028 ± 0.003 | 0.3010 | - | - | - |
| A1/2/3ulhf_R | 0.022 ± 0.003 | 0.028 ± 0.003 | 0.029 ± 0.004 | 0.2521 | - | - | - |
| A1/2/3tonIa_L | 0.023 ± 0.003 | 0.028 ± 0.004 | 0.030 ± 0.003 | 0.3414 | - | - | - |
| A1/2/3tonIa_R | 0.023 ± 0.003 | 0.023 ± 0.003 | 0.028 ± 0.003 | 0.4625 | - | - | - |
| A2_L | 0.023 ± 0.003 | 0.028 ± 0.003 | 0.024 ± 0.003 | 0.3087 | - | - | - |
| A2_R | 0.023 ± 0.003 | 0.026 ± 0.003 | 0.025 ± 0.004 | 0.7996 | - | - | - |
| A1/2/3tru_L | 0.022 ± 0.003 | 0.029 ± 0.004 | 0.030 ± 0.004 | 0.3125 | - | - | - |
| A1/2/3tru_R | 0.023 ± 0.003 | 0.027 ± 0.003 | 0.023 ± 0.003 | 0.6925 | - | - | - |
| A23d_L | 0.019 ± 0.003 | 0.023 ± 0.003 | 0.026 ± 0.003 | 0.1908 | - | - | - |
| A23d_R | 0.021 ± 0.002 | 0.023 ± 0.003 | 0.027 ± 0.003 | 0.3015 | - | - | - |
| * SEM, standard error mean. Grp-E, the group of patients with epilepsy and IDH mutation; Grp-nE, the group of patients with non-epilepsy and IDH mutation; Grp-GnE, the group of patients with non-epilepsy, glioblastoma (grade 4) and, IDH wildtype. L, left hemispheric node; R, right hemispheric node. | | | | | | | |
| **Continue Table s8** | | | | | | | |
| **Node** | **Grp-E**  **(mean ± SEM)** | **Grp-nE**  **(mean ± SEM)** | **Grp-GnE**  **(mean ± SEM)** | **One-way ANOVA**  **(*p* value)** | **Post-hoc analysis with Sidak correction**  **(*p* value)** | | |
|  |  |  |  |  | **Grp-E VS**  **Grp-nE** | **Grp-E VS**  **Grp-GnE** | **Grp-nE VS**  **Grp-GnE** |
| A4ll_L | 0.022 ± 0.003 | 0.029 ± 0.003 | 0.025 ± 0.003 | 0.1917 | - | - | - |
| A4ll_R | 0.020 ± 0.003 | 0.026 ± 0.003 | 0.023 ± 0.004 | 0.2769 | - | - | - |
| A1/2/3ll_L | 0.023 ± 0.003 | 0.024 ± 0.003 | 0.023 ± 0.003 | 0.9893 | - | - | - |
| A1/2/3ll_R | 0.023 ± 0.003 | 0.023 ± 0.003 | 0.024 ± 0.003 | 0.9740 | - | - | - |
| A23v_L | 0.021 ± 0.003 | 0.022 ± 0.003 | 0.025 ± 0.003 | 0.6994 | - | - | - |
| A23v_R | 0.020 ± 0.003 | 0.023 ± 0.003 | 0.025 ± 0.003 | 0.5679 | - | - | - |
| A24cd_L | 0.022 ± 0.002 | 0.023 ± 0.002 | 0.027 ± 0.003 | 0.3691 | - | - | - |
| A24cd_R | 0.022 ± 0.003 | 0.027 ± 0.004 | 0.027 ± 0.004 | 0.4249 | - | - | - |
| A23c_L | 0.019 ± 0.002 | 0.026 ± 0.003 | 0.024 ± 0.003 | 0.1293 | - | - | - |
| A23c_R | 0.021 ± 0.003 | 0.027 ± 0.004 | 0.026 ± 0.002 | 0.3494 | - | - | - |
| A32sg_L | 0.019 ± 0.002 | 0.023 ± 0.003 | 0.027 ± 0.004 | 0.2103 | - | - | - |
| A32sg_R | 0.020 ± 0.002 | 0.026 ± 0.003 | 0.027 ± 0.003 | 0.1555 | - | - | - |
| mPMtha_L | 0.021 ± 0.002 | 0.021 ± 0.003 | 0.022 ± 0.002 | 0.9665 | - | - | - |
| mPMtha_R | 0.023 ± 0.003 | 0.030 ± 0.003 | 0.024 ± 0.003 | 0.2430 | - | - | - |
| Stha_L | 0.025 ± 0.004 | 0.027 ± 0.003 | 0.024 ± 0.003 | 0.7748 | - | - | - |
| Stha_R | 0.019 ± 0.002 | 0.027 ± 0.003 | 0.034 ± 0.005 | 0.0098 | 0.1828 | 0.0085 | 0.4405 |
| * SEM, standard error mean. Grp-E, the group of patients with epilepsy and IDH mutation; Grp-nE, the group of patients with non-epilepsy and IDH mutation; Grp-GnE, the group of patients with non-epilepsy, glioblastoma (grade 4) and, IDH wildtype. L, left hemispheric node; R, right hemispheric node. | | | | | | | |

| **Table s9. Nodal Efficiency in patients with different molecular diagnoses and different history of preoperative epilepsy (left glioma)** | | | | | | | |
| --- | --- | --- | --- | --- | --- | --- | --- |
| **Node** | **Grp-E**  **(mean ± SEM)** | **Grp-nE**  **(mean ± SEM)** | **Grp-GnE**  **(mean ± SEM)** | **One-way ANOVA**  **(*p* value)** | **Post-hoc analysis with Sidak correction**  **(*p* value)** | | |
|  |  |  |  |  | **Grp-E VS**  **Grp-nE** | **Grp-E VS**  **Grp-GnE** | **Grp-nE VS**  **Grp-GnE** |
| A6m_L | 0.237 ± 0.015 | 0.202 ± 0.006 | 0.216 ± 0.018 | 0.0946 | - | - | - |
| A6m_R | 0.229 ± 0.017 | 0.199 ± 0.006 | 0.214 ± 0.015 | 0.2236 | - | - | - |
| A4hf_L | 0.236 ± 0.012 | 0.217 ± 0.006 | 0.220 ± 0.015 | 0.3393 | - | - | - |
| A4hf_R | 0.239 ± 0.016 | 0.213 ± 0.008 | 0.209 ± 0.013 | 0.1979 | - | - | - |
| A6cdl_L | 0.217 ± 0.012 | 0.209 ± 0.006 | 0.208 ± 0.016 | 0.7973 | - | - | - |
| A6cdl_R | 0.225 ± 0.015 | 0.211 ± 0.008 | 0.203 ± 0.020 | 0.5747 | - | - | - |
| A4ul_L | 0.240 ± 0.014 | 0.200 ± 0.006 | 0.210 ± 0.019 | 0.0420 | 0.0405 | 0.3447 | 0.9489 |
| A4ul_R | 0.228 ± 0.015 | 0.213 ± 0.006 | 0.206 ± 0.016 | 0.4723 | - | - | - |
| A4t_L | 0.229 ± 0.013 | 0.206 ± 0.008 | 0.234 ± 0.021 | 0.2590 | - | - | - |
| A4t_R | 0.237 ± 0.016 | 0.208 ± 0.009 | 0.219 ± 0.021 | 0.2924 | - | - | - |
| A4tl_L | 0.228 ± 0.016 | 0.199 ± 0.007 | 0.206 ± 0.014 | 0.1971 | - | - | - |
| A4tl_R | 0.239 ± 0.015 | 0.209 ± 0.007 | 0.218 ± 0.024 | 0.2255 | - | - | - |
| A6cvl_L | 0.228 ± 0.013 | 0.198 ± 0.006 | 0.215 ± 0.025 | 0.2050 | - | - | - |
| A6cvl_R | 0.235 ± 0.015 | 0.205 ± 0.008 | 0.224 ± 0.022 | 0.2438 | - | - | - |
| A1/2/3ulhf_L | 0.229 ± 0.010 | 0.215 ± 0.006 | 0.215 ± 0.015 | 0.4673 | - | - | - |
| A1/2/3ulhf_R | 0.236 ± 0.013 | 0.216 ± 0.007 | 0.207 ± 0.010 | 0.1559 | - | - | - |
| A1/2/3tonIa_L | 0.248 ± 0.017 | 0.207 ± 0.008 | 0.205 ± 0.016 | 0.0483 | 0.0723 | 0.1695 | 0.9996 |
| A1/2/3tonIa_R | 0.231 ± 0.011 | 0.202 ± 0.005 | 0.218 ± 0.022 | 0.1184 | - | - | - |
| A2_L | 0.244 ± 0.018 | 0.217 ± 0.008 | 0.207 ± 0.014 | 0.1746 | - | - | - |
| A2_R | 0.242 ± 0.015 | 0.214 ± 0.007 | 0.210 ± 0.009 | 0.1093 | - | - | - |
| A1/2/3tru_L | 0.242 ± 0.017 | 0.209 ± 0.006 | 0.228 ± 0.021 | 0.1941 | - | - | - |
| A1/2/3tru_R | 0.244 ± 0.019 | 0.213 ± 0.009 | 0.217 ± 0.023 | 0.3082 | - | - | - |
| A23d_L | 0.245 ± 0.014 | 0.209 ± 0.007 | 0.231 ± 0.018 | 0.0783 | - | - | - |
| A23d_R | 0.244 ± 0.014 | 0.217 ± 0.008 | 0.235 ± 0.019 | 0.2467 | - | - | - |
| * SEM, standard error mean. Grp-E, the group of patients with epilepsy and IDH mutation; Grp-nE, the group of patients with non-epilepsy and IDH mutation; Grp-GnE, the group of patients with non-epilepsy, glioblastoma (grade 4) and, IDH wildtype. L, left hemispheric node; R, right hemispheric node. | | | | | | | |
| **Continue Table s9** | | | | | | | |
| **Node** | **Grp-E**  **(mean ± SEM)** | **Grp-nE**  **(mean ± SEM)** | **Grp-GnE**  **(mean ± SEM)** | **One-way ANOVA**  **(*p* value)** | **Post-hoc analysis with Sidak correction**  **(*p* value)** | | |
|  |  |  |  |  | **Grp-E VS**  **Grp-nE** | **Grp-E VS**  **Grp-GnE** | **Grp-nE VS**  **Grp-GnE** |
| A4ll_L | 0.217 ± 0.012 | 0.200 ± 0.008 | 0.214 ± 0.021 | 0.5460 | - | - | - |
| A4ll_R | 0.227 ± 0.019 | 0.207 ± 0.008 | 0.216 ± 0.026 | 0.6309 | - | - | - |
| A1/2/3ll_L | 0.241 ± 0.017 | 0.202 ± 0.006 | 0.198 ± 0.014 | 0.0333 | 0.0570 | 0.1140 | 0.9958 |
| A1/2/3ll_R | 0.237 ± 0.016 | 0.212 ± 0.006 | 0.229 ± 0.027 | 0.3943 | - | - | - |
| A23v_L | 0.244 ± 0.019 | 0.219 ± 0.007 | 0.229 ± 0.015 | 0.4131 | - | - | - |
| A23v_R | 0.231 ± 0.016 | 0.203 ± 0.006 | 0.207 ± 0.010 | 0.1583 | - | - | - |
| A24cd_L | 0.241 ± 0.014 | 0.217 ± 0.008 | 0.220 ± 0.016 | 0.2821 | - | - | - |
| A24cd_R | 0.245 ± 0.017 | 0.223 ± 0.009 | 0.221 ± 0.008 | 0.3895 | - | - | - |
| A23c_L | 0.240 ± 0.017 | 0.205 ± 0.006 | 0.207 ± 0.012 | 0.0888 | - | - | - |
| A23c_R | 0.240 ± 0.016 | 0.203 ± 0.007 | 0.211 ± 0.010 | 0.0556 | - | - | - |
| A32sg_L | 0.230 ± 0.012 | 0.209 ± 0.007 | 0.198 ± 0.012 | 0.1175 | - | - | - |
| A32sg_R | 0.242 ± 0.016 | 0.216 ± 0.007 | 0.213 ± 0.015 | 0.2321 | - | - | - |
| mPMtha_L | 0.220 ± 0.015 | 0.194 ± 0.007 | 0.212 ± 0.027 | 0.3589 | - | - | - |
| mPMtha_R | 0.239 ± 0.017 | 0.205 ± 0.007 | 0.231 ± 0.027 | 0.2195 | - | - | - |
| Stha_L | 0.205 ± 0.012 | 0.207 ± 0.006 | 0.204 ± 0.014 | 0.9874 | - | - | - |
| Stha_R | 0.244 ± 0.017 | 0.197 ± 0.009 | 0.223 ± 0.023 | 0.0591 | - | - | - |
| * SEM, standard error mean. Grp-E, the group of patients with epilepsy and IDH mutation; Grp-nE, the group of patients with non-epilepsy and IDH mutation; Grp-GnE, the group of patients with non-epilepsy, glioblastoma (grade 4) and, IDH wildtype. L, left hemispheric node; R, right hemispheric node. | | | | | | | |

| **Table s10. Nodal Efficiency in patients with different molecular diagnoses and different history of preoperative epilepsy (right glioma)** | | | | | | | |
| --- | --- | --- | --- | --- | --- | --- | --- |
| **Node** | **Grp-E**  **(mean ± SEM)** | **Grp-nE**  **(mean ± SEM)** | **Grp-GnE**  **(mean ± SEM)** | **One-way ANOVA**  **(*p* value)** | **Post-hoc analysis with Sidak correction**  **(*p* value)** | | |
|  |  |  |  |  | **Grp-E VS**  **Grp-nE** | **Grp-E VS**  **Grp-GnE** | **Grp-nE VS**  **Grp-GnE** |
| A6m_L | 0.277 ± 0.032 | 0.220 ± 0.017 | 0.217 ± 0.016 | 0.1708 | - | - | - |
| A6m_R | 0.279 ± 0.030 | 0.217 ± 0.014 | 0.218 ± 0.017 | 0.1041 | - | - | - |
| A4hf_L | 0.284 ± 0.031 | 0.256 ± 0.027 | 0.232 ± 0.022 | 0.4788 | - | - | - |
| A4hf_R | 0.262 ± 0.030 | 0.221 ± 0.017 | 0.215 ± 0.018 | 0.3436 | - | - | - |
| A6cdl_L | 0.231 ± 0.019 | 0.251 ± 0.031 | 0.217 ± 0.012 | 0.6178 | - | - | - |
| A6cdl_R | 0.235 ± 0.018 | 0.221 ± 0.015 | 0.226 ± 0.021 | 0.8493 | - | - | - |
| A4ul_L | 0.253 ± 0.024 | 0.226 ± 0.026 | 0.223 ± 0.018 | 0.6408 | - | - | - |
| A4ul_R | 0.267 ± 0.023 | 0.226 ± 0.029 | 0.236 ± 0.017 | 0.4586 | - | - | - |
| A4t_L | 0.263 ± 0.022 | 0.257 ± 0.030 | 0.211 ± 0.015 | 0.3358 | - | - | - |
| A4t_R | 0.287 ± 0.033 | 0.251 ± 0.030 | 0.218 ± 0.012 | 0.2922 | - | - | - |
| A4tl_L | 0.274 ± 0.029 | 0.250 ± 0.030 | 0.199 ± 0.013 | 0.1930 | - | - | - |
| A4tl_R | 0.273 ± 0.031 | 0.235 ± 0.023 | 0.203 ± 0.016 | 0.2063 | - | - | - |
| A6cvl_L | 0.269 ± 0.027 | 0.219 ± 0.019 | 0.209 ± 0.017 | 0.1634 | - | - | - |
| A6cvl_R | 0.261 ± 0.025 | 0.203 ± 0.013 | 0.228 ± 0.021 | 0.1387 | - | - | - |
| A1/2/3ulhf_L | 0.265 ± 0.024 | 0.250 ± 0.029 | 0.250 ± 0.022 | 0.8856 | - | - | - |
| A1/2/3ulhf_R | 0.283 ± 0.028 | 0.232 ± 0.024 | 0.246 ± 0.023 | 0.3429 | - | - | - |
| A1/2/3tonIa_L | 0.267 ± 0.030 | 0.237 ± 0.024 | 0.206 ± 0.016 | 0.3068 | - | - | - |
| A1/2/3tonIa_R | 0.248 ± 0.023 | 0.247 ± 0.027 | 0.220 ± 0.016 | 0.7075 | - | - | - |
| A2_L | 0.242 ± 0.018 | 0.230 ± 0.022 | 0.218 ± 0.019 | 0.7289 | - | - | - |
| A2_R | 0.264 ± 0.027 | 0.239 ± 0.030 | 0.224 ± 0.020 | 0.6253 | - | - | - |
| A1/2/3tru_L | 0.297 ± 0.031 | 0.251 ± 0.028 | 0.222 ± 0.014 | 0.1842 | - | - | - |
| A1/2/3tru_R | 0.256 ± 0.023 | 0.236 ± 0.024 | 0.219 ± 0.010 | 0.5248 | - | - | - |
| A23d_L | 0.252 ± 0.018 | 0.247 ± 0.031 | 0.226 ± 0.015 | 0.7499 | - | - | - |
| A23d_R | 0.271 ± 0.028 | 0.253 ± 0.024 | 0.223 ± 0.013 | 0.4390 | - | - | - |
| * SEM, standard error mean. Grp-E, the group of patients with epilepsy and IDH mutation; Grp-nE, the group of patients with non-epilepsy and IDH mutation; Grp-GnE, the group of patients with non-epilepsy, glioblastoma (grade 4) and, IDH wildtype. L, left hemispheric node; R, right hemispheric node. | | | | | | | |
| **Continue Table s10** | | | | | | | |
| **Node** | **Grp-E**  **(mean ± SEM)** | **Grp-nE**  **(mean ± SEM)** | **Grp-GnE**  **(mean ± SEM)** | **One-way ANOVA**  **(*p* value)** | **Post-hoc analysis with Sidak correction**  **(*p* value)** | | |
|  |  |  |  |  | **Grp-E VS**  **Grp-nE** | **Grp-E VS**  **Grp-GnE** | **Grp-nE VS**  **Grp-GnE** |
| A4ll_L | 0.255 ± 0.023 | 0.191 ± 0.017 | 0.205 ± 0.013 | 0.0531 | - | - | - |
| A4ll_R | 0.268 ± 0.029 | 0.215 ± 0.021 | 0.210 ± 0.012 | 0.1775 | - | - | - |
| A1/2/3ll_L | 0.272 ± 0.032 | 0.193 ± 0.007 | 0.215 ± 0.009 | 0.0350 | 0.0358 | 0.2588 | 0.8839 |
| A1/2/3ll_R | 0.279 ± 0.034 | 0.235 ± 0.029 | 0.212 ± 0.014 | 0.2934 | - | - | - |
| A23v_L | 0.273 ± 0.035 | 0.249 ± 0.028 | 0.233 ± 0.017 | 0.6692 | - | - | - |
| A23v_R | 0.246 ± 0.020 | 0.194 ± 0.013 | 0.216 ± 0.016 | 0.0943 | - | - | - |
| A24cd_L | 0.251 ± 0.019 | 0.260 ± 0.030 | 0.207 ± 0.013 | 0.3101 | - | - | - |
| A24cd_R | 0.261 ± 0.020 | 0.256 ± 0.031 | 0.234 ± 0.021 | 0.7651 | - | - | - |
| A23c_L | 0.248 ± 0.026 | 0.231 ± 0.024 | 0.222 ± 0.021 | 0.7605 | - | - | - |
| A23c_R | 0.286 ± 0.031 | 0.223 ± 0.016 | 0.214 ± 0.017 | 0.0802 | - | - | - |
| A32sg_L | 0.255 ± 0.030 | 0.228 ± 0.017 | 0.218 ± 0.012 | 0.5310 | - | - | - |
| A32sg_R | 0.272 ± 0.025 | 0.226 ± 0.028 | 0.223 ± 0.017 | 0.3134 | - | - | - |
| mPMtha_L | 0.247 ± 0.025 | 0.236 ± 0.030 | 0.195 ± 0.014 | 0.4012 | - | - | - |
| mPMtha_R | 0.255 ± 0.022 | 0.204 ± 0.018 | 0.207 ± 0.019 | 0.1314 | - | - | - |
| Stha_L | 0.275 ± 0.031 | 0.198 ± 0.013 | 0.214 ± 0.016 | 0.0494 | 0.0587 | 0.2353 | 0.9633 |
| Stha_R | 0.245 ± 0.020 | 0.210 ± 0.013 | 0.208 ± 0.013 | 0.2242 | - | - | - |
| * SEM, standard error mean. Grp-E, the group of patients with epilepsy and IDH mutation; Grp-nE, the group of patients with non-epilepsy and IDH mutation; Grp-GnE, the group of patients with non-epilepsy, glioblastoma (grade 4) and, IDH wildtype. L, left hemispheric node; R, right hemispheric node. | | | | | | | |

| **Table s11. Nodal Local Efficiency in patients with different molecular diagnoses and different history of preoperative epilepsy (left glioma)** | | | | | | | |
| --- | --- | --- | --- | --- | --- | --- | --- |
| **Node** | **Grp-E**  **(mean ± SEM)** | **Grp-nE**  **(mean ± SEM)** | **Grp-GnE**  **(mean ± SEM)** | **One-way ANOVA**  **(*p* value)** | **Post-hoc analysis with Sidak correction**  **(*p* value)** | | |
|  |  |  |  |  | **Grp-E VS**  **Grp-nE** | **Grp-E VS**  **Grp-GnE** | **Grp-nE VS**  **Grp-GnE** |
| A6m_L | 0.046 ± 0.004 | 0.045 ± 0.003 | 0.047 ± 0.005 | 0.9285 | - | - | - |
| A6m_R | 0.049 ± 0.004 | 0.051 ± 0.003 | 0.048 ± 0.005 | 0.8572 | - | - | - |
| A4hf_L | 0.048 ± 0.003 | 0.049 ± 0.003 | 0.053 ± 0.006 | 0.6424 | - | - | - |
| A4hf_R | 0.051 ± 0.004 | 0.047 ± 0.003 | 0.047 ± 0.005 | 0.6516 | - | - | - |
| A6cdl_L | 0.048 ± 0.004 | 0.050 ± 0.003 | 0.046 ± 0.005 | 0.7289 | - | - | - |
| A6cdl_R | 0.050 ± 0.004 | 0.047 ± 0.004 | 0.058 ± 0.009 | 0.3902 | - | - | - |
| A4ul_L | 0.047 ± 0.003 | 0.047 ± 0.003 | 0.053 ± 0.004 | 0.4930 | - | - | - |
| A4ul_R | 0.046 ± 0.004 | 0.051 ± 0.003 | 0.052 ± 0.007 | 0.6730 | - | - | - |
| A4t_L | 0.047 ± 0.004 | 0.047 ± 0.003 | 0.044 ± 0.005 | 0.8923 | - | - | - |
| A4t_R | 0.048 ± 0.004 | 0.047 ± 0.003 | 0.051 ± 0.007 | 0.8713 | - | - | - |
| A4tl_L | 0.046 ± 0.003 | 0.056 ± 0.004 | 0.043 ± 0.004 | 0.0697 | - | - | - |
| A4tl_R | 0.050 ± 0.003 | 0.049 ± 0.003 | 0.047 ± 0.005 | 0.8420 | - | - | - |
| A6cvl_L | 0.049 ± 0.004 | 0.045 ± 0.003 | 0.040 ± 0.006 | 0.4104 | - | - | - |
| A6cvl_R | 0.047 ± 0.004 | 0.055 ± 0.004 | 0.042 ± 0.006 | 0.1276 | - | - | - |
| A1/2/3ulhf_L | 0.055 ± 0.005 | 0.049 ± 0.003 | 0.059 ± 0.007 | 0.3057 | - | - | - |
| A1/2/3ulhf_R | 0.049 ± 0.004 | 0.052 ± 0.003 | 0.051 ± 0.005 | 0.8899 | - | - | - |
| A1/2/3tonIa_L | 0.050 ± 0.004 | 0.049 ± 0.003 | 0.050 ± 0.006 | 0.9611 | - | - | - |
| A1/2/3tonIa_R | 0.048 ± 0.004 | 0.047 ± 0.004 | 0.048 ± 0.006 | 0.9775 | - | - | - |
| A2_L | 0.046 ± 0.003 | 0.046 ± 0.003 | 0.046 ± 0.005 | 0.9988 | - | - | - |
| A2_R | 0.046 ± 0.004 | 0.053 ± 0.004 | 0.050 ± 0.004 | 0.4355 | - | - | - |
| A1/2/3tru_L | 0.049 ± 0.004 | 0.048 ± 0.004 | 0.052 ± 0.007 | 0.9083 | - | - | - |
| A1/2/3tru_R | 0.049 ± 0.004 | 0.049 ± 0.004 | 0.053 ± 0.006 | 0.8535 | - | - | - |
| A23d_L | 0.044 ± 0.005 | 0.049 ± 0.005 | 0.038 ± 0.005 | 0.4588 | - | - | - |
| A23d_R | 0.043 ± 0.003 | 0.045 ± 0.004 | 0.035 ± 0.004 | 0.3061 | - | - | - |
| * SEM, standard error mean. Grp-E, the group of patients with epilepsy and IDH mutation; Grp-nE, the group of patients with non-epilepsy and IDH mutation; Grp-GnE, the group of patients with non-epilepsy, glioblastoma (grade 4) and, IDH wildtype. L, left hemispheric node; R, right hemispheric node. | | | | | | | |
| **Continue Table s11** | | | | | | | |
| **Node** | **Grp-E**  **(mean ± SEM)** | **Grp-nE**  **(mean ± SEM)** | **Grp-GnE**  **(mean ± SEM)** | **One-way ANOVA**  **(*p* value)** | **Post-hoc analysis with Sidak correction**  **(*p* value)** | | |
|  |  |  |  |  | **Grp-E VS**  **Grp-nE** | **Grp-E VS**  **Grp-GnE** | **Grp-nE VS**  **Grp-GnE** |
| A4ll_L | 0.041 ± 0.004 | 0.051 ± 0.004 | 0.042 ± 0.006 | 0.1626 | - | - | - |
| A4ll_R | 0.046 ± 0.003 | 0.048 ± 0.004 | 0.054 ± 0.006 | 0.4153 | - | - | - |
| A1/2/3ll_L | 0.047 ± 0.004 | 0.051 ± 0.004 | 0.049 ± 0.005 | 0.7777 | - | - | - |
| A1/2/3ll_R | 0.054 ± 0.004 | 0.056 ± 0.003 | 0.052 ± 0.006 | 0.8520 | - | - | - |
| A23v_L | 0.049 ± 0.004 | 0.044 ± 0.003 | 0.046 ± 0.005 | 0.5866 | - | - | - |
| A23v_R | 0.048 ± 0.004 | 0.053 ± 0.004 | 0.040 ± 0.006 | 0.1519 | - | - | - |
| A24cd_L | 0.049 ± 0.005 | 0.052 ± 0.003 | 0.044 ± 0.004 | 0.5030 | - | - | - |
| A24cd_R | 0.046 ± 0.004 | 0.051 ± 0.003 | 0.048 ± 0.006 | 0.6925 | - | - | - |
| A23c_L | 0.049 ± 0.003 | 0.054 ± 0.004 | 0.048 ± 0.006 | 0.5244 | - | - | - |
| A23c_R | 0.050 ± 0.004 | 0.055 ± 0.004 | 0.049 ± 0.006 | 0.5773 | - | - | - |
| A32sg_L | 0.043 ± 0.003 | 0.048 ± 0.003 | 0.048 ± 0.005 | 0.4582 | - | - | - |
| A32sg_R | 0.045 ± 0.004 | 0.051 ± 0.003 | 0.049 ± 0.005 | 0.4839 | - | - | - |
| mPMtha_L | 0.038 ± 0.003 | 0.047 ± 0.003 | 0.044 ± 0.006 | 0.1463 | - | - | - |
| mPMtha_R | 0.045 ± 0.003 | 0.054 ± 0.004 | 0.046 ± 0.006 | 0.2399 | - | - | - |
| Stha_L | 0.052 ± 0.004 | 0.043 ± 0.003 | 0.042 ± 0.005 | 0.0928 | - | - | - |
| Stha_R | 0.047 ± 0.005 | 0.058 ± 0.003 | 0.038 ± 0.005 | 0.0106 | 0.1320 | 0.5170 | 0.0130 |
| * SEM, standard error mean. Grp-E, the group of patients with epilepsy and IDH mutation; Grp-nE, the group of patients with non-epilepsy and IDH mutation; Grp-GnE, the group of patients with non-epilepsy, glioblastoma (grade 4) and, IDH wildtype. L, left hemispheric node; R, right hemispheric node. | | | | | | | |

| **Table s12. Nodal Local Efficiency in patients with different molecular diagnoses and different history of preoperative epilepsy (right glioma)** | | | | | | | |
| --- | --- | --- | --- | --- | --- | --- | --- |
| **Node** | **Grp-E**  **(mean ± SEM)** | **Grp-nE**  **(mean ± SEM)** | **Grp-GnE**  **(mean ± SEM)** | **One-way ANOVA**  **(*p* value)** | **Post-hoc analysis with Sidak correction**  **(*p* value)** | | |
|  |  |  |  |  | **Grp-E VS**  **Grp-nE** | **Grp-E VS**  **Grp-GnE** | **Grp-nE VS**  **Grp-GnE** |
| A6m_L | 0.045 ± 0.005 | 0.053 ± 0.006 | 0.052 ± 0.007 | 0.5944 | - | - | - |
| A6m_R | 0.046 ± 0.006 | 0.047 ± 0.005 | 0.062 ± 0.014 | 0.3435 | - | - | - |
| A4hf_L | 0.041 ± 0.004 | 0.044 ± 0.005 | 0.053 ± 0.007 | 0.3254 | - | - | - |
| A4hf_R | 0.044 ± 0.005 | 0.050 ± 0.005 | 0.057 ± 0.006 | 0.2891 | - | - | - |
| A6cdl_L | 0.053 ± 0.005 | 0.049 ± 0.005 | 0.048 ± 0.005 | 0.8313 | - | - | - |
| A6cdl_R | 0.047 ± 0.005 | 0.054 ± 0.005 | 0.043 ± 0.005 | 0.3932 | - | - | - |
| A4ul_L | 0.043 ± 0.006 | 0.054 ± 0.007 | 0.052 ± 0.006 | 0.3760 | - | - | - |
| A4ul_R | 0.039 ± 0.005 | 0.047 ± 0.006 | 0.049 ± 0.006 | 0.4946 | - | - | - |
| A4t_L | 0.043 ± 0.005 | 0.049 ± 0.006 | 0.058 ± 0.008 | 0.2417 | - | - | - |
| A4t_R | 0.046 ± 0.005 | 0.048 ± 0.005 | 0.050 ± 0.005 | 0.8819 | - | - | - |
| A4tl_L | 0.041 ± 0.004 | 0.048 ± 0.006 | 0.052 ± 0.005 | 0.3309 | - | - | - |
| A4tl_R | 0.044 ± 0.005 | 0.042 ± 0.004 | 0.046 ± 0.005 | 0.8717 | - | - | - |
| A6cvl_L | 0.044 ± 0.005 | 0.059 ± 0.008 | 0.054 ± 0.007 | 0.2325 | - | - | - |
| A6cvl_R | 0.042 ± 0.005 | 0.048 ± 0.006 | 0.045 ± 0.005 | 0.6749 | - | - | - |
| A1/2/3ulhf_L | 0.051 ± 0.007 | 0.056 ± 0.006 | 0.052 ± 0.005 | 0.8786 | - | - | - |
| A1/2/3ulhf_R | 0.042 ± 0.005 | 0.053 ± 0.006 | 0.054 ± 0.007 | 0.2715 | - | - | - |
| A1/2/3tonIa_L | 0.046 ± 0.006 | 0.050 ± 0.006 | 0.061 ± 0.006 | 0.2238 | - | - | - |
| A1/2/3tonIa_R | 0.047 ± 0.005 | 0.041 ± 0.005 | 0.052 ± 0.008 | 0.4045 | - | - | - |
| A2_L | 0.047 ± 0.005 | 0.051 ± 0.005 | 0.044 ± 0.004 | 0.7051 | - | - | - |
| A2_R | 0.047 ± 0.005 | 0.046 ± 0.005 | 0.047 ± 0.006 | 0.9877 | - | - | - |
| A1/2/3tru_L | 0.042 ± 0.005 | 0.053 ± 0.006 | 0.053 ± 0.006 | 0.3230 | - | - | - |
| A1/2/3tru_R | 0.048 ± 0.006 | 0.050 ± 0.006 | 0.045 ± 0.005 | 0.8581 | - | - | - |
| A23d_L | 0.037 ± 0.004 | 0.041 ± 0.005 | 0.047 ± 0.005 | 0.3520 | - | - | - |
| A23d_R | 0.045 ± 0.004 | 0.037 ± 0.004 | 0.053 ± 0.004 | 0.0394 | 0.3731 | 0.4912 | 0.0350 |
| * SEM, standard error mean. Grp-E, the group of patients with epilepsy and IDH mutation; Grp-nE, the group of patients with non-epilepsy and IDH mutation; Grp-GnE, the group of patients with non-epilepsy, glioblastoma (grade 4) and, IDH wildtype. L, left hemispheric node; R, right hemispheric node. | | | | | | | |
| **Continue Table s12** | | | | | | | |
| **Node** | **Grp-E**  **(mean ± SEM)** | **Grp-nE**  **(mean ± SEM)** | **Grp-GnE**  **(mean ± SEM)** | **One-way ANOVA**  **(*p* value)** | **Post-hoc analysis with Sidak correction**  **(*p* value)** | | |
|  |  |  |  |  | **Grp-E VS**  **Grp-nE** | **Grp-E VS**  **Grp-GnE** | **Grp-nE VS**  **Grp-GnE** |
| A4ll_L | 0.041 ± 0.005 | 0.056 ± 0.006 | 0.050 ± 0.006 | 0.1725 | - | - | - |
| A4ll_R | 0.039 ± 0.005 | 0.047 ± 0.005 | 0.042 ± 0.006 | 0.5969 | - | - | - |
| A1/2/3ll_L | 0.047 ± 0.006 | 0.046 ± 0.005 | 0.043 ± 0.006 | 0.9005 | - | - | - |
| A1/2/3ll_R | 0.047 ± 0.005 | 0.041 ± 0.005 | 0.046 ± 0.005 | 0.6511 | - | - | - |
| A23v_L | 0.044 ± 0.005 | 0.038 ± 0.005 | 0.048 ± 0.004 | 0.4235 | - | - | - |
| A23v_R | 0.038 ± 0.004 | 0.039 ± 0.004 | 0.049 ± 0.007 | 0.3623 | - | - | - |
| A24cd_L | 0.046 ± 0.004 | 0.045 ± 0.005 | 0.050 ± 0.006 | 0.8060 | - | - | - |
| A24cd_R | 0.045 ± 0.005 | 0.048 ± 0.006 | 0.050 ± 0.007 | 0.8737 | - | - | - |
| A23c_L | 0.040 ± 0.005 | 0.049 ± 0.006 | 0.047 ± 0.007 | 0.4418 | - | - | - |
| A23c_R | 0.042 ± 0.005 | 0.047 ± 0.006 | 0.052 ± 0.004 | 0.4081 | - | - | - |
| A32sg_L | 0.036 ± 0.004 | 0.039 ± 0.005 | 0.048 ± 0.006 | 0.2559 | - | - | - |
| A32sg_R | 0.038 ± 0.004 | 0.045 ± 0.005 | 0.054 ± 0.005 | 0.0928 | - | - | - |
| mPMtha_L | 0.041 ± 0.005 | 0.037 ± 0.005 | 0.042 ± 0.004 | 0.7371 | - | - | - |
| mPMtha_R | 0.045 ± 0.005 | 0.054 ± 0.005 | 0.047 ± 0.006 | 0.4035 | - | - | - |
| Stha_L | 0.047 ± 0.006 | 0.049 ± 0.006 | 0.045 ± 0.006 | 0.9163 | - | - | - |
| Stha_R | 0.038 ± 0.004 | 0.049 ± 0.005 | 0.058 ± 0.006 | 0.0242 | 0.2814 | 0.0220 | 0.5234 |
| * SEM, standard error mean. Grp-E, the group of patients with epilepsy and IDH mutation; Grp-nE, the group of patients with non-epilepsy and IDH mutation; Grp-GnE, the group of patients with non-epilepsy, glioblastoma (grade 4) and, IDH wildtype. L, left hemispheric node; R, right hemispheric node. | | | | | | | |

| **Table s13. Nodal Vulnerability in patients with different molecular diagnoses and different history of preoperative epilepsy (left glioma)** | | | | | | | |
| --- | --- | --- | --- | --- | --- | --- | --- |
| **Node** | **Grp-E**  **(mean ± SEM)** | **Grp-nE**  **(mean ± SEM)** | **Grp-GnE**  **(mean ± SEM)** | **One-way ANOVA**  **(*p* value)** | **Post-hoc analysis with Sidak correction**  **(*p* value)** | | |
|  |  |  |  |  | **Grp-E VS**  **Grp-nE** | **Grp-E VS**  **Grp-GnE** | **Grp-nE VS**  **Grp-GnE** |
| A6m_L | 0.012 ± 0.003 | 0.005 ± 0.002 | 0.006 ± 0.003 | 0.1924 | - | - | - |
| A6m_R | 0.001 ± 0.001 | 0.001 ± 0.001 | 0.006 ± 0.003 | 0.0872 | - | - | - |
| A4hf_L | 0.008 ± 0.003 | 0.010 ± 0.003 | 0.013 ± 0.005 | 0.6657 | - | - | - |
| A4hf_R | 0.006 ± 0.002 | 0.010 ± 0.002 | 0.007 ± 0.005 | 0.5112 | - | - | - |
| A6cdl_L | 0.001 ± 0.002 | 0.008 ± 0.002 | 0.009 ± 0.005 | 0.0515 | - | - | - |
| A6cdl_R | -0.003 ± 0.003 | 0.008 ± 0.003 | 0.002 ± 0.003 | 0.0211 | 0.0168 | 0.5999 | 0.5813 |
| A4ul_L | 0.003 ± 0.004 | 0.011 ± 0.004 | 0.005 ± 0.002 | 0.3039 | - | - | - |
| A4ul_R | 0.009 ± 0.003 | 0.006 ± 0.002 | 0.002 ± 0.002 | 0.3138 | - | - | - |
| A4t_L | 0.005 ± 0.002 | 0.005 ± 0.003 | 0.017 ± 0.005 | 0.0413 | > 0.9999 | 0.0659 | 0.0568 |
| A4t_R | 0.007 ± 0.003 | 0.006 ± 0.002 | 0.006 ± 0.002 | 0.9734 | - | - | - |
| A4tl_L | 0.004 ± 0.002 | 0.003 ± 0.002 | 0.005 ± 0.004 | 0.8102 | - | - | - |
| A4tl_R | 0.008 ± 0.003 | 0.005 ± 0.002 | 0.007 ± 0.005 | 0.6911 | - | - | - |
| A6cvl_L | 0.006 ± 0.002 | 0.004 ± 0.002 | 0.008 ± 0.006 | 0.7708 | - | - | - |
| A6cvl_R | 0.006 ± 0.002 | 0.004 ± 0.002 | 0.006 ± 0.003 | 0.8478 | - | - | - |
| A1/2/3ulhf_L | 0.010 ± 0.003 | 0.008 ± 0.002 | 0.007 ± 0.003 | 0.7574 | - | - | - |
| A1/2/3ulhf_R | 0.010 ± 0.003 | 0.009 ± 0.003 | 0.003 ± 0.002 | 0.2598 | - | - | - |
| A1/2/3tonIa_L | 0.012 ± 0.003 | 0.005 ± 0.002 | 0.002 ± 0.004 | 0.0500 | - | - | - |
| A1/2/3tonIa_R | 0.007 ± 0.002 | 0.003 ± 0.002 | 0.010 ± 0.004 | 0.2194 | - | - | - |
| A2_L | 0.011 ± 0.003 | 0.013 ± 0.003 | 0.009 ± 0.005 | 0.7868 | - | - | - |
| A2_R | 0.012 ± 0.003 | 0.010 ± 0.003 | 0.007 ± 0.002 | 0.6561 | - | - | - |
| A1/2/3tru_L | 0.012 ± 0.004 | 0.011 ± 0.004 | 0.015 ± 0.005 | 0.8832 | - | - | - |
| A1/2/3tru_R | 0.008 ± 0.003 | 0.010 ± 0.002 | 0.004 ± 0.002 | 0.4140 | - | - | - |
| A23d_L | 0.010 ± 0.004 | 0.006 ± 0.002 | 0.005 ± 0.003 | 0.6082 | - | - | - |
| A23d_R | 0.008 ± 0.003 | 0.009 ± 0.002 | 0.007 ± 0.002 | 0.8799 | - | - | - |
| * SEM, standard error mean. Grp-E, the group of patients with epilepsy and IDH mutation; Grp-nE, the group of patients with non-epilepsy and IDH mutation; Grp-GnE, the group of patients with non-epilepsy, glioblastoma (grade 4) and, IDH wildtype. L, left hemispheric node; R, right hemispheric node. | | | | | | | |
| **Continue Table s13** | | | | | | | |
| **Node** | **Grp-E**  **(mean ± SEM)** | **Grp-nE**  **(mean ± SEM)** | **Grp-GnE**  **(mean ± SEM)** | **One-way ANOVA**  **(*p* value)** | **Post-hoc analysis with Sidak correction**  **(*p* value)** | | |
|  |  |  |  |  | **Grp-E VS**  **Grp-nE** | **Grp-E VS**  **Grp-GnE** | **Grp-nE VS**  **Grp-GnE** |
| A4ll_L | 0.001 ± 0.002 | 0.004 ± 0.002 | 0.006 ± 0.002 | 0.3314 | - | - | - |
| A4ll_R | 0.001 ± 0.002 | 0.003 ± 0.002 | 0.011 ± 0.006 | 0.0339 | 0.7491 | 0.0291 | 0.1468 |
| A1/2/3ll_L | 0.005 ± 0.002 | 0.003 ± 0.002 | 0.001 ± 0.002 | 0.3696 | - | - | - |
| A1/2/3ll_R | 0.008 ± 0.003 | 0.008 ± 0.002 | 0.012 ± 0.004 | 0.5452 | - | - | - |
| A23v_L | 0.008 ± 0.003 | 0.013 ± 0.002 | 0.012 ± 0.004 | 0.3652 | - | - | - |
| A23v_R | 0.008 ± 0.003 | 0.003 ± 0.002 | 0.007 ± 0.005 | 0.3205 | - | - | - |
| A24cd_L | 0.008 ± 0.003 | 0.012 ± 0.003 | 0.008 ± 0.003 | 0.5520 | - | - | - |
| A24cd_R | 0.006 ± 0.002 | 0.013 ± 0.003 | 0.017 ± 0.006 | 0.1198 | - | - | - |
| A23c_L | 0.009 ± 0.003 | 0.006 ± 0.002 | 0.004 ± 0.004 | 0.5421 | - | - | - |
| A23c_R | 0.008 ± 0.002 | 0.008 ± 0.003 | 0.011 ± 0.004 | 0.7647 | - | - | - |
| A32sg_L | 0.004 ± 0.002 | 0.006 ± 0.003 | 0.004 ± 0.003 | 0.6436 | - | - | - |
| A32sg_R | 0.005 ± 0.002 | 0.010 ± 0.002 | 0.007 ± 0.003 | 0.2106 | - | - | - |
| mPMtha_L | 0.004 ± 0.004 | 0.002 ± 0.002 | 0.003 ± 0.003 | 0.8327 | - | - | - |
| mPMtha_R | 0.009 ± 0.003 | 0.008 ± 0.003 | 0.012 ± 0.006 | 0.8124 | - | - | - |
| Stha_L | -0.002 ± 0.002 | 0.008 ± 0.002 | 0.005 ± 0.003 | 0.0054 | 0.0042 | 0.1973 | 0.8088 |
| Stha_R | 0.007 ± 0.002 | -0.002 ± 0.002 | 0.008 ± 0.003 | 0.0017 | 0.0052 | 0.9905 | 0.0139 |
| * SEM, standard error mean. Grp-E, the group of patients with epilepsy and IDH mutation; Grp-nE, the group of patients with non-epilepsy and IDH mutation; Grp-GnE, the grp of patients with non-epilepsy, glioblastoma (grade 4) and, IDH wildtype. L, left hemispheric node; R, right hemispheric node. | | | | | | | |

| **Table s14. Nodal Vulnerability in patients with different molecular diagnoses and different history of preoperative epilepsy (right glioma)** | | | | | | | |
| --- | --- | --- | --- | --- | --- | --- | --- |
| **Node** | **Grp-E**  **(mean ± SEM)** | **Grp-nE**  **(mean ± SEM)** | **Grp-GnE**  **(mean ± SEM)** | **One-way ANOVA**  **(*p* value)** | **Post-hoc analysis with Sidak correction**  **(*p* value)** | | |
|  |  |  |  |  | **Grp-E VS**  **Grp-nE** | **Grp-E VS**  **Grp-GnE** | **Grp-nE VS**  **Grp-GnE** |
| A6m_L | 0.005 ± 0.002 | 0.005 ± 0.003 | 0.010 ± 0.005 | 0.5213 | - | - | - |
| A6m_R | 0.014 ± 0.004 | 0.006 ± 0.003 | 0.007 ± 0.004 | 0.2100 | - | - | - |
| A4hf_L | 0.009 ± 0.003 | 0.010 ± 0.002 | 0.013 ± 0.004 | 0.7443 | - | - | - |
| A4hf_R | 0.001 ± 0.002 | 0.004 ± 0.003 | 0.005 ± 0.005 | 0.6991 | - | - | - |
| A6cdl_L | -0.001 ± 0.002 | 0.009 ± 0.003 | 0.004 ± 0.002 | 0.0095 | 0.0072 | 0.3884 | 0.4076 |
| A6cdl_R | 0.004 ± 0.003 | 0.006 ± 0.004 | 0.009 ± 0.004 | 0.5471 | - | - | - |
| A4ul_L | 0.009 ± 0.004 | 0.010 ± 0.003 | 0.002 ± 0.003 | 0.3102 | - | - | - |
| A4ul_R | 0.006 ± 0.003 | 0.009 ± 0.003 | 0.011 ± 0.004 | 0.6982 | - | - | - |
| A4t_L | 0.009 ± 0.003 | 0.009 ± 0.003 | 0.007 ± 0.007 | 0.9578 | - | - | - |
| A4t_R | 0.010 ± 0.004 | 0.009 ± 0.003 | 0.006 ± 0.003 | 0.7275 | - | - | - |
| A4tl_L | 0.006 ± 0.002 | 0.008 ± 0.003 | 0.002 ± 0.002 | 0.3731 | - | - | - |
| A4tl_R | 0.006 ± 0.002 | 0.005 ± 0.003 | 0.001 ± 0.003 | 0.4017 | - | - | - |
| A6cvl_L | 0.005 ± 0.003 | 0.005 ± 0.003 | 0.001 ± 0.003 | 0.4886 | - | - | - |
| A6cvl_R | 0.004 ± 0.003 | 0.004 ± 0.003 | 0.005 ± 0.003 | 0.9703 | - | - | - |
| A1/2/3ulhf_L | 0.015 ± 0.005 | 0.011 ± 0.002 | 0.018 ± 0.005 | 0.4889 | - | - | - |
| A1/2/3ulhf_R | 0.020 ± 0.006 | 0.005 ± 0.003 | 0.013 ± 0.005 | 0.0878 | - | - | - |
| A1/2/3tonIa_L | 0.006 ± 0.003 | 0.011 ± 0.006 | 0.002 ± 0.003 | 0.4685 | - | - | - |
| A1/2/3tonIa_R | 0.001 ± 0.002 | 0.012 ± 0.005 | 0.010 ± 0.006 | 0.0924 | - | - | - |
| A2_L | 0.006 ± 0.003 | 0.014 ± 0.005 | 0.005 ± 0.002 | 0.1543 | - | - | - |
| A2_R | 0.006 ± 0.003 | 0.004 ± 0.002 | 0.005 ± 0.003 | 0.8184 | - | - | - |
| A1/2/3tru_L | 0.017 ± 0.004 | 0.005 ± 0.002 | 0.006 ± 0.003 | 0.0243 | 0.0386 | 0.0986 | 0.9976 |
| A1/2/3tru_R | 0.004 ± 0.003 | 0.008 ± 0.003 | 0.011 ± 0.004 | 0.4889 | - | - | - |
| A23d_L | 0.004 ± 0.002 | 0.006 ± 0.003 | 0.011 ± 0.006 | 0.4142 | - | - | - |
| A23d_R | 0.004 ± 0.002 | 0.013 ± 0.003 | 0.013 ± 0.004 | 0.0776 | - | - | - |
| * SEM, standard error mean. Grp-E, the group of patients with epilepsy and IDH mutation; Grp-nE, the group of patients with non-epilepsy and IDH mutation; Grp-GnE, the group of patients with non-epilepsy, glioblastoma (grade 4) and, IDH wildtype. L, left hemispheric node; R, right hemispheric node. | | | | | | | |
| **Continue Table s14** | | | | | | | |
| **Node** | **Grp-E**  **(mean ± SEM)** | **Grp-nE**  **(mean ± SEM)** | **Grp-GnE**  **(mean ± SEM)** | **One-way ANOVA**  **(*p* value)** | **Post-hoc analysis with Sidak correction**  **(*p* value)** | | |
|  |  |  |  |  | **Grp-E VS**  **Grp-nE** | **Grp-E VS**  **Grp-GnE** | **Grp-nE VS**  **Grp-GnE** |
| A4ll_L | 0.006 ± 0.003 | -0.002 ± 0.002 | 0.000 ± 0.002 | 0.0514 | - | - | - |
| A4ll_R | 0.003 ± 0.002 | 0.000 ± 0.002 | 0.003 ± 0.003 | 0.5536 | - | - | - |
| A1/2/3ll_L | 0.003 ± 0.002 | 0.007 ± 0.003 | 0.013 ± 0.004 | 0.1062 | - | - | - |
| A1/2/3ll_R | 0.011 ± 0.003 | 0.015 ± 0.005 | 0.007 ± 0.003 | 0.4405 | - | - | - |
| A23v_L | 0.004 ± 0.003 | 0.006 ± 0.002 | 0.011 ± 0.004 | 0.3298 | - | - | - |
| A23v_R | 0.004 ± 0.003 | 0.006 ± 0.003 | 0.008 ± 0.004 | 0.6992 | - | - | - |
| A24cd_L | 0.007 ± 0.003 | 0.011 ± 0.003 | 0.007 ± 0.005 | 0.6943 | - | - | - |
| A24cd_R | 0.009 ± 0.003 | 0.012 ± 0.004 | 0.010 ± 0.003 | 0.8667 | - | - | - |
| A23c_L | 0.004 ± 0.003 | 0.001 ± 0.002 | 0.013 ± 0.004 | 0.0174 | 0.7823 | 0.0999 | 0.0160 |
| A23c_R | 0.013 ± 0.004 | 0.008 ± 0.003 | 0.005 ± 0.004 | 0.3458 | - | - | - |
| A32sg_L | 0.005 ± 0.003 | 0.004 ± 0.003 | 0.010 ± 0.005 | 0.5679 | - | - | - |
| A32sg_R | 0.013 ± 0.003 | 0.006 ± 0.002 | 0.009 ± 0.006 | 0.4739 | - | - | - |
| mPMtha_L | 0.000 ± 0.002 | 0.003 ± 0.002 | -0.003 ± 0.002 | 0.1217 | - | - | - |
| mPMtha_R | 0.004 ± 0.002 | 0.000 ± 0.003 | 0.003 ± 0.003 | 0.5463 | - | - | - |
| Stha_L | 0.002 ± 0.002 | -0.001 ± 0.003 | 0.004 ± 0.003 | 0.3912 | - | - | - |
| Stha_R | 0.005 ± 0.003 | 0.002 ± 0.002 | 0.006 ± 0.004 | 0.6780 | - | - | - |
| * SEM, standard error mean. Grp-E, the group of patients with epilepsy and IDH mutation; Grp-nE, the group of patients with non-epilepsy and IDH mutation; Grp-GnE, the group of patients with non-epilepsy, glioblastoma (grade 4) and, IDH wildtype. L, left hemispheric node; R, right hemispheric node. | | | | | | | |

| **Table s15. Global properties in patients with IDH mutation, intact chromosome 1p/19q, and different history of preoperative epilepsy** | | | | | | |
| --- | --- | --- | --- | --- | --- | --- |
| Properties | Left hemispheric glioma  (mean ± SEM) | | | Right hemispheric glioma  (mean ± SEM) | | |
|  | Epilepsy | Non-epilepsy | *p* value | Epilepsy | Non-epilepsy | *p* value |
| Clustering coefficient | 0.083 ± 0.007 | 0.095 ± 0.003 | 0.1369 | 0.066 ± 0.010 | 0.097 ± 0.006 | 0.0407 |
| Fault tolerance | 2.394 ± 0.150 | 2.700 ± 0.043 | 0.0578 | 2.027 ± 0.200 | 2.677 ± 0.065 | 0.0284 |
| Global efficiency | 0.330 ± 0.024 | 0.277 ± 0.008 | 0.0462 | 0.392 ± 0.039 | 0.277 ± 0.012 | 0.0419 |
| Local efficiency | 0.328 ± 0.024 | 0.275 ± 0.008 | 0.0461 | 0.389 ± 0.039 | 0.275 ± 0.012 | 0.0421 |
| Shortest path length | 3.908 ± 0.189 | 4.391 ± 0.098 | 0.0310 | 3.424 ± 0.273 | 4.372 ± 0.139 | 0.0229 |
| Transitivity | 0.021 ± 0.002 | 0.023 ± 0.001 | 0.2949 | 0.018 ± 0.002 | 0.024 ± 0.001 | 0.0754 |
| Vulnerability | 0.060 ± 0.005 | 0.058 ± 0.004 | 0.8042 | 0.056 ± 0.007 | 0.053 ± 0.004 | 0.7337 |
| * SEM, standard error mean. | | | | | | |

| **Table s16. Global properties in patients with IDH mutation co-deletion chromosome 1p/19q, and different history of preoperative epilepsy** | | | | | | |
| --- | --- | --- | --- | --- | --- | --- |
| Properties | Left hemispheric glioma  (mean ± SEM) | | | Right hemispheric glioma  (mean ± SEM) | | |
|  | Epilepsy | Non-epilepsy | *p* value | Epilepsy | Non-epilepsy | *p* value |
| Clustering coefficient | 0.079 ± 0.008 | 0.094 ± 0.004 | 0.0892 | 0.063 ± 0.011 | 0.092 ± 0.008 | 0.0350 |
| Fault tolerance | 2.340 ± 0.160 | 2.694 ± 0.069 | 0.0394 | 2.071 ± 0.255 | 2.589 ± 0.172 | 0.0935 |
| Global efficiency | 0.339 ± 0.031 | 0.276 ± 0.006 | 0.0335 | 0.401 ± 0.057 | 0.294 ± 0.023 | 0.0806 |
| Local efficiency | 0.336 ± 0.030 | 0.274 ± 0.005 | 0.0333 | 0.398 ± 0.057 | 0.292 ± 0.023 | 0.0800 |
| Shortest path length | 3.842 ± 0.226 | 4.370 ± 0.087 | 0.0263 | 3.539 ± 0.346 | 4.271 ± 0.194 | 0.0655 |
| Transitivity | 0.020 ± 0.002 | 0.023 ± 0.001 | 0.1210 | 0.015 ± 0.003 | 0.023 ± 0.003 | 0.0349 |
| Vulnerability | 0.060 ± 0.007 | 0.061 ± 0.005 | 0.8428 | 0.057 ± 0.004 | 0.056 ± 0.006 | 0.9116 |
| * SEM, standard error mean. | | | | | | |

| **Table s17. Nodal betweenness in patients with IDH mutation, intact chromosome 1p/19q, and different history of preoperative epilepsy** | | | | | | |
| --- | --- | --- | --- | --- | --- | --- |
| **Node** | Left hemispheric glioma  (mean ± SEM) | | *p* value | Right hemispheric glioma  (mean ± SEM) | | *p* value |
|  | Epilepsy | Non-epilepsy |  | Epilepsy | Non-epilepsy |  |
| A6m_L | 32.348 ± 6.606 | 12.984 ± 3.112 | 0.0123 | 15.683 ± 3.208 | 17.981 ± 4.159 | 0.6872 |
| A6m_R | 15.440 ± 4.318 | 12.015 ± 2.327 | 0.4942 | 40.145 ± 5.108 | 21.310 ± 6.299 | 0.0328 |
| A4hf_L | 23.971 ± 5.797 | 23.516 ± 3.718 | 0.9485 | 23.077 ± 3.217 | 24.611 ± 7.529 | 0.8683 |
| A4hf_R | 17.685 ± 2.862 | 18.266 ± 3.215 | 0.8963 | 13.192 ± 4.503 | 14.582 ± 3.401 | 0.8107 |
| A6cdl_L | 11.298 ± 3.224 | 22.522 ± 5.075 | 0.0794 | 8.331 ± 1.340 | 28.330 ± 5.655 | 0.0004 |
| A6cdl_R | 12.052 ± 2.681 | 26.223 ± 5.896 | 0.0423 | 18.373 ± 5.610 | 24.310 ± 5.822 | 0.4909 |
| A4ul_L | 22.438 ± 4.466 | 23.947 ± 3.841 | 0.8035 | 22.737 ± 4.860 | 14.252 ± 3.155 | 0.1558 |
| A4ul_R | 23.737 ± 5.220 | 16.755 ± 2.763 | 0.2497 | 30.432 ± 6.956 | 25.697 ± 4.910 | 0.5868 |
| A4t_L | 14.442 ± 3.111 | 22.696 ± 4.986 | 0.1834 | 21.136 ± 5.365 | 17.995 ± 4.230 | 0.6565 |
| A4t_R | 26.150 ± 5.316 | 24.286 ± 3.633 | 0.7773 | 19.305 ± 6.386 | 18.642 ± 3.000 | 0.9241 |
| A4tl_L | 20.387 ± 4.107 | 15.570 ± 3.189 | 0.3693 | 17.068 ± 3.758 | 10.272 ± 1.865 | 0.1101 |
| A4tl_R | 15.546 ± 2.803 | 16.377 ± 3.336 | 0.8541 | 15.562 ± 4.321 | 15.139 ± 3.099 | 0.9380 |
| A6cvl_L | 18.962 ± 4.314 | 15.308 ± 4.508 | 0.5719 | 18.340 ± 4.256 | 15.276 ± 3.345 | 0.5842 |
| A6cvl_R | 16.652 ± 4.024 | 20.018 ± 3.930 | 0.5628 | 17.524 ± 5.483 | 19.397 ± 3.816 | 0.7834 |
| A1/2/3ulhf_L | 13.377 ± 2.052 | 21.980 ± 3.565 | 0.0509 | 17.405 ± 5.420 | 33.411 ± 4.840 | 0.0427 |
| A1/2/3ulhf_R | 25.008 ± 4.340 | 23.093 ± 4.002 | 0.7532 | 28.163 ± 6.681 | 31.750 ± 6.795 | 0.7226 |
| A1/2/3tonIa_L | 28.167 ± 4.749 | 18.053 ± 2.556 | 0.0714 | 26.834 ± 5.376 | 16.440 ± 4.700 | 0.1703 |
| A1/2/3tonIa_R | 23.063 ± 4.560 | 10.769 ± 1.891 | 0.0180 | 14.503 ± 5.269 | 25.608 ± 7.688 | 0.2820 |
| A2_L | 22.817 ± 4.496 | 23.399 ± 3.956 | 0.9248 | 20.926 ± 4.161 | 16.851 ± 3.459 | 0.4700 |
| A2_R | 23.802 ± 4.027 | 24.304 ± 5.081 | 0.9405 | 24.914 ± 6.223 | 20.175 ± 4.930 | 0.5643 |
| A1/2/3tru_L | 19.181 ± 3.711 | 26.487 ± 5.680 | 0.3050 | 27.592 ± 6.235 | 20.457 ± 4.585 | 0.3718 |
| A1/2/3tru_R | 22.021 ± 4.247 | 24.203 ± 4.231 | 0.7246 | 17.124 ± 2.989 | 30.147 ± 7.083 | 0.1420 |
| A23d_L | 22.319 ± 3.744 | 15.720 ± 2.707 | 0.1685 | 18.456 ± 3.814 | 25.974 ± 6.135 | 0.3499 |
| A23d_R | 25.862 ± 3.618 | 22.828 ± 2.778 | 0.5179 | 19.817 ± 3.745 | 22.779 ± 3.087 | 0.5572 |
| A4ll_L | 13.390 ± 3.419 | 16.601 ± 3.202 | 0.5073 | 18.044 ± 4.707 | 11.019 ± 2.867 | 0.2120 |
| A4ll_R | 8.494 ± 1.672 | 14.636 ± 3.603 | 0.1459 | 7.580 ± 1.409 | 12.447 ± 3.185 | 0.2217 |
| A1/2/3ll_L | 23.038 ± 3.476 | 13.868 ± 2.781 | 0.0504 | 12.636 ± 3.155 | 27.627 ± 4.482 | 0.0177 |
| A1/2/3ll_R | 22.844 ± 4.013 | 19.678 ± 3.700 | 0.5743 | 19.302 ± 3.208 | 18.700 ± 4.271 | 0.9174 |
| A23v_L | 20.194 ± 4.785 | 19.178 ± 1.944 | 0.8462 | 22.408 ± 4.221 | 22.127 ± 4.599 | 0.9663 |
| A23v_R | 20.269 ± 5.002 | 14.200 ± 2.578 | 0.2928 | 14.464 ± 3.454 | 23.983 ± 4.596 | 0.1358 |
| A24cd_L | 22.798 ± 3.804 | 29.718 ± 4.972 | 0.2909 | 20.172 ± 4.773 | 20.976 ± 6.140 | 0.9240 |
| A24cd_R | 22.783 ± 4.484 | 32.247 ± 4.844 | 0.1709 | 16.716 ± 3.995 | 27.743 ± 5.648 | 0.1525 |
| A23c_L | 22.131 ± 4.049 | 15.998 ± 3.089 | 0.2446 | 19.885 ± 4.604 | 25.550 ± 5.779 | 0.4802 |
| A23c_R | 20.065 ± 3.34 | 19.877 ± 3.901 | 0.9718 | 26.222 ± 5.349 | 16.704 ± 3.170 | 0.1355 |
| A32sg_L | 13.750 ± 2.876 | 22.033 ± 3.887 | 0.1053 | 17.737 ± 3.342 | 17.950 ± 3.280 | 0.9656 |
| A32sg_R | 17.038 ± 3.456 | 25.011 ± 3.537 | 0.1244 | 25.275 ± 4.169 | 25.380 ± 8.166 | 0.9919 |
| mPMtha_L | 12.933 ± 5.090 | 14.126 ± 3.022 | 0.8434 | 11.920 ± 2.664 | 7.026 ± 2.421 | 0.2011 |
| mPMtha_R | 19.213 ± 3.412 | 24.452 ± 4.937 | 0.4040 | 10.784 ± 2.291 | 12.423 ± 2.876 | 0.6806 |
| Stha_L | 9.769 ± 2.132 | 18.110 ± 3.764 | 0.0710 | 16.636 ± 4.017 | 13.481 ± 2.771 | 0.5270 |
| Stha_R | 17.927 ± 3.918 | 14.333 ± 2.668 | 0.4604 | 24.488 ± 5.752 | 13.046 ± 3.504 | 0.0999 |
| * SEM, standard error mean. L, left hemispheric node; R, right hemispheric node. | | | | | | |

| **Table s18. Nodal degree centrality in patients with IDH mutation, intact chromosome 1p/19q, and different history of preoperative epilepsy** | | | | | | |
| --- | --- | --- | --- | --- | --- | --- |
| **Node** | Left hemispheric glioma  (mean ± SEM) | | *p* value | Right hemispheric glioma  (mean ± SEM) | | *p* value |
|  | Epilepsy | Non-epilepsy |  | Epilepsy | Non-epilepsy |  |
| A6m_L | 4.115 ± 0.511 | 2.814 ± 0.189 | 0.0227 | 4.073 ± 0.884 | 3.331 ± 0.342 | 0.4243 |
| A6m_R | 3.643 ± 0.626 | 2.940 ± 0.248 | 0.3065 | 4.512 ± 0.678 | 3.356 ± 0.377 | 0.1436 |
| A4hf_L | 3.488 ± 0.262 | 3.361 ± 0.191 | 0.7021 | 3.934 ± 0.638 | 3.843 ± 0.497 | 0.9127 |
| A4hf_R | 3.628 ± 0.454 | 3.182 ± 0.366 | 0.4587 | 3.372 ± 0.660 | 3.241 ± 0.370 | 0.8620 |
| A6cdl_L | 2.993 ± 0.292 | 3.249 ± 0.234 | 0.5058 | 3.198 ± 0.372 | 3.260 ± 0.158 | 0.8755 |
| A6cdl_R | 3.392 ± 0.387 | 3.471 ± 0.316 | 0.8786 | 2.811 ± 0.238 | 3.568 ± 0.422 | 0.1673 |
| A4ul_L | 3.641 ± 0.421 | 3.481 ± 0.216 | 0.7392 | 3.468 ± 0.228 | 3.551 ± 0.381 | 0.8660 |
| A4ul_R | 3.856 ± 0.407 | 2.904 ± 0.198 | 0.0439 | 4.113 ± 0.602 | 3.871 ± 0.354 | 0.7307 |
| A4t_L | 3.258 ± 0.273 | 3.328 ± 0.286 | 0.8628 | 3.787 ± 0.544 | 3.130 ± 0.314 | 0.3015 |
| A4t_R | 3.781 ± 0.367 | 3.498 ± 0.403 | 0.6157 | 3.678 ± 0.457 | 3.325 ± 0.210 | 0.4778 |
| A4tl_L | 3.672 ± 0.510 | 2.889 ± 0.210 | 0.1670 | 3.502 ± 0.565 | 2.743 ± 0.226 | 0.2082 |
| A4tl_R | 3.798 ± 0.453 | 3.062 ± 0.193 | 0.1457 | 2.883 ± 0.285 | 2.894 ± 0.337 | 0.9821 |
| A6cvl_L | 3.553 ± 0.357 | 2.937 ± 0.209 | 0.1493 | 3.668 ± 0.444 | 3.196 ± 0.388 | 0.4450 |
| A6cvl_R | 3.279 ± 0.345 | 3.207 ± 0.347 | 0.8862 | 3.312 ± 0.259 | 3.766 ± 0.462 | 0.4430 |
| A1/2/3ulhf_L | 3.074 ± 0.219 | 3.491 ± 0.181 | 0.1598 | 3.446 ± 0.330 | 4.322 ± 0.496 | 0.1886 |
| A1/2/3ulhf_R | 3.727 ± 0.411 | 3.533 ± 0.224 | 0.6834 | 4.358 ± 0.647 | 4.128 ± 0.492 | 0.7828 |
| A1/2/3tonIa_L | 4.457 ± 0.576 | 3.421 ± 0.314 | 0.1272 | 4.437 ± 0.791 | 3.112 ± 0.294 | 0.1145 |
| A1/2/3tonIa_R | 3.606 ± 0.210 | 2.864 ± 0.159 | 0.0086 | 3.176 ± 0.499 | 3.451 ± 0.314 | 0.6456 |
| A2_L | 4.240 ± 0.648 | 3.366 ± 0.272 | 0.2246 | 3.251 ± 0.214 | 3.360 ± 0.402 | 0.8310 |
| A2_R | 3.905 ± 0.391 | 3.308 ± 0.280 | 0.2302 | 3.669 ± 0.659 | 3.467 ± 0.491 | 0.8105 |
| A1/2/3tru_L | 4.105 ± 0.656 | 3.336 ± 0.236 | 0.2796 | 4.587 ± 0.818 | 3.349 ± 0.267 | 0.1444 |
| A1/2/3tru_R | 4.075 ± 0.662 | 3.210 ± 0.347 | 0.2603 | 3.698 ± 0.548 | 3.306 ± 0.228 | 0.5023 |
| A23d_L | 3.867 ± 0.465 | 2.992 ± 0.182 | 0.0899 | 3.179 ± 0.273 | 3.543 ± 0.353 | 0.4534 |
| A23d_R | 4.108 ± 0.325 | 3.451 ± 0.234 | 0.1158 | 3.689 ± 0.547 | 3.313 ± 0.223 | 0.5159 |
| A4ll_L | 3.067 ± 0.297 | 3.093 ± 0.294 | 0.9518 | 3.612 ± 0.486 | 2.944 ± 0.229 | 0.2144 |
| A4ll_R | 3.257 ± 0.567 | 3.163 ± 0.242 | 0.8804 | 3.536 ± 0.759 | 2.869 ± 0.247 | 0.3907 |
| A1/2/3ll_L | 4.124 ± 0.602 | 2.856 ± 0.209 | 0.0551 | 3.291 ± 0.345 | 3.346 ± 0.206 | 0.8915 |
| A1/2/3ll_R | 3.795 ± 0.562 | 3.084 ± 0.161 | 0.2332 | 3.902 ± 0.647 | 2.928 ± 0.257 | 0.1590 |
| A23v_L | 3.776 ± 0.548 | 3.468 ± 0.193 | 0.6018 | 4.350 ± 0.909 | 3.676 ± 0.389 | 0.4890 |
| A23v_R | 3.599 ± 0.536 | 2.933 ± 0.169 | 0.2458 | 3.268 ± 0.346 | 3.359 ± 0.301 | 0.8490 |
| A24cd_L | 3.825 ± 0.340 | 3.629 ± 0.263 | 0.6558 | 3.759 ± 0.523 | 3.072 ± 0.286 | 0.2538 |
| A24cd_R | 3.735 ± 0.437 | 3.835 ± 0.413 | 0.8722 | 3.507 ± 0.329 | 3.871 ± 0.518 | 0.5908 |
| A23c_L | 4.179 ± 0.697 | 3.053 ± 0.217 | 0.1327 | 3.898 ± 0.719 | 3.556 ± 0.540 | 0.7117 |
| A23c_R | 3.770 ± 0.477 | 3.087 ± 0.226 | 0.2078 | 4.407 ± 0.751 | 3.160 ± 0.405 | 0.1500 |
| A32sg_L | 2.953 ± 0.186 | 3.172 ± 0.248 | 0.4984 | 4.001 ± 0.873 | 3.207 ± 0.225 | 0.3616 |
| A32sg_R | 3.733 ± 0.521 | 3.428 ± 0.292 | 0.6164 | 3.922 ± 0.600 | 3.627 ± 0.402 | 0.6883 |
| mPMtha_L | 3.084 ± 0.399 | 2.744 ± 0.187 | 0.4498 | 3.714 ± 0.573 | 2.614 ± 0.278 | 0.0890 |
| mPMtha_R | 3.634 ± 0.416 | 3.442 ± 0.266 | 0.7028 | 3.320 ± 0.405 | 2.997 ± 0.402 | 0.5926 |
| Stha_L | 2.785 ± 0.300 | 3.077 ± 0.207 | 0.4372 | 3.820 ± 0.885 | 3.197 ± 0.375 | 0.5094 |
| Stha_R | 3.888 ± 0.500 | 3.130 ± 0.307 | 0.2101 | 4.441 ± 0.825 | 2.895 ± 0.232 | 0.0687 |
| * SEM, standard error mean. L, left hemispheric node; R, right hemispheric node. | | | | | | |

| **Table s19. Nodal clustering coefficient in patients with IDH mutation, intact chromosome 1p/19q, and different history of preoperative epilepsy** | | | | | | |
| --- | --- | --- | --- | --- | --- | --- |
| **Node** | Left hemispheric glioma  (mean ± SEM) | | *p* value | Right hemispheric glioma  (mean ± SEM) | | *p* value |
|  | Epilepsy | Non-epilepsy |  | Epilepsy | Non-epilepsy |  |
| A6m_L | 0.026 ± 0.003 | 0.023 ± 0.002 | 0.4714 | 0.026 ± 0.003 | 0.030 ± 0.004 | 0.5738 |
| A6m_R | 0.025 ± 0.003 | 0.028 ± 0.003 | 0.5670 | 0.023 ± 0.003 | 0.034 ± 0.008 | 0.2884 |
| A4hf_L | 0.025 ± 0.003 | 0.026 ± 0.002 | 0.7301 | 0.024 ± 0.003 | 0.029 ± 0.005 | 0.4325 |
| A4hf_R | 0.029 ± 0.003 | 0.03 ± 0.004 | 0.8575 | 0.027 ± 0.004 | 0.030 ± 0.004 | 0.5897 |
| A6cdl_L | 0.026 ± 0.003 | 0.026 ± 0.002 | 0.8657 | 0.032 ± 0.003 | 0.025 ± 0.003 | 0.0755 |
| A6cdl_R | 0.025 ± 0.003 | 0.025 ± 0.002 | 0.9517 | 0.028 ± 0.005 | 0.024 ± 0.003 | 0.4425 |
| A4ul_L | 0.027 ± 0.003 | 0.026 ± 0.002 | 0.7729 | 0.023 ± 0.003 | 0.028 ± 0.004 | 0.2799 |
| A4ul_R | 0.024 ± 0.003 | 0.025 ± 0.003 | 0.7989 | 0.021 ± 0.003 | 0.025 ± 0.003 | 0.3758 |
| A4t_L | 0.024 ± 0.003 | 0.025 ± 0.003 | 0.6902 | 0.026 ± 0.004 | 0.029 ± 0.004 | 0.6914 |
| A4t_R | 0.022 ± 0.002 | 0.026 ± 0.003 | 0.3344 | 0.029 ± 0.004 | 0.025 ± 0.003 | 0.5391 |
| A4tl_L | 0.022 ± 0.002 | 0.028 ± 0.003 | 0.1409 | 0.023 ± 0.003 | 0.030 ± 0.004 | 0.2172 |
| A4tl_R | 0.025 ± 0.002 | 0.027 ± 0.002 | 0.4994 | 0.026 ± 0.003 | 0.024 ± 0.002 | 0.6234 |
| A6cvl_L | 0.024 ± 0.002 | 0.026 ± 0.003 | 0.5087 | 0.022 ± 0.002 | 0.030 ± 0.005 | 0.2153 |
| A6cvl_R | 0.024 ± 0.003 | 0.030 ± 0.003 | 0.1926 | 0.023 ± 0.003 | 0.024 ± 0.003 | 0.7532 |
| A1/2/3ulhf_L | 0.031 ± 0.004 | 0.026 ± 0.002 | 0.2863 | 0.032 ± 0.004 | 0.028 ± 0.003 | 0.3560 |
| A1/2/3ulhf_R | 0.026 ± 0.004 | 0.027 ± 0.003 | 0.8173 | 0.028 ± 0.004 | 0.029 ± 0.004 | 0.8766 |
| A1/2/3tonIa_L | 0.027 ± 0.003 | 0.026 ± 0.002 | 0.9585 | 0.023 ± 0.003 | 0.030 ± 0.003 | 0.1122 |
| A1/2/3tonIa_R | 0.027 ± 0.003 | 0.026 ± 0.003 | 0.8488 | 0.026 ± 0.003 | 0.028 ± 0.003 | 0.6577 |
| A2_L | 0.025 ± 0.003 | 0.026 ± 0.003 | 0.7076 | 0.028 ± 0.003 | 0.024 ± 0.003 | 0.3226 |
| A2_R | 0.026 ± 0.003 | 0.028 ± 0.003 | 0.7378 | 0.027 ± 0.004 | 0.025 ± 0.004 | 0.8489 |
| A1/2/3tru_L | 0.027 ± 0.003 | 0.027 ± 0.003 | 0.8473 | 0.026 ± 0.004 | 0.030 ± 0.004 | 0.5633 |
| A1/2/3tru_R | 0.025 ± 0.003 | 0.024 ± 0.002 | 0.8138 | 0.023 ± 0.003 | 0.023 ± 0.003 | 0.9964 |
| A23d_L | 0.025 ± 0.004 | 0.031 ± 0.008 | 0.5425 | 0.024 ± 0.004 | 0.026 ± 0.003 | 0.6008 |
| A23d_R | 0.022 ± 0.002 | 0.026 ± 0.002 | 0.2935 | 0.023 ± 0.003 | 0.027 ± 0.003 | 0.3283 |
| A4ll_L | 0.022 ± 0.002 | 0.028 ± 0.004 | 0.2317 | 0.024 ± 0.004 | 0.025 ± 0.003 | 0.8782 |
| A4ll_R | 0.022 ± 0.002 | 0.025 ± 0.002 | 0.4103 | 0.022 ± 0.003 | 0.023 ± 0.004 | 0.9842 |
| A1/2/3ll_L | 0.024 ± 0.003 | 0.027 ± 0.003 | 0.5074 | 0.028 ± 0.005 | 0.023 ± 0.003 | 0.3870 |
| A1/2/3ll_R | 0.028 ± 0.003 | 0.028 ± 0.003 | 0.9623 | 0.023 ± 0.003 | 0.024 ± 0.003 | 0.7714 |
| A23v_L | 0.027 ± 0.003 | 0.026 ± 0.004 | 0.8426 | 0.022 ± 0.003 | 0.025 ± 0.003 | 0.4384 |
| A23v_R | 0.026 ± 0.004 | 0.031 ± 0.003 | 0.3342 | 0.024 ± 0.004 | 0.025 ± 0.003 | 0.9338 |
| A24cd_L | 0.025 ± 0.005 | 0.026 ± 0.002 | 0.8229 | 0.025 ± 0.003 | 0.027 ± 0.003 | 0.6588 |
| A24cd_R | 0.026 ± 0.004 | 0.027 ± 0.002 | 0.8896 | 0.024 ± 0.004 | 0.027 ± 0.004 | 0.6701 |
| A23c_L | 0.026 ± 0.002 | 0.026 ± 0.002 | 0.9891 | 0.021 ± 0.003 | 0.024 ± 0.003 | 0.5041 |
| A23c_R | 0.025 ± 0.003 | 0.027 ± 0.003 | 0.5846 | 0.023 ± 0.003 | 0.026 ± 0.002 | 0.5038 |
| A32sg_L | 0.024 ± 0.003 | 0.028 ± 0.002 | 0.2608 | 0.022 ± 0.003 | 0.027 ± 0.004 | 0.3511 |
| A32sg_R | 0.024 ± 0.002 | 0.031 ± 0.004 | 0.1594 | 0.021 ± 0.003 | 0.027 ± 0.003 | 0.1524 |
| mPMtha_L | 0.019 ± 0.002 | 0.027 ± 0.002 | 0.0122 | 0.022 ± 0.003 | 0.022 ± 0.002 | 0.9813 |
| mPMtha_R | 0.023 ± 0.002 | 0.030 ± 0.004 | 0.1069 | 0.027 ± 0.004 | 0.024 ± 0.003 | 0.6052 |
| Stha_L | 0.028 ± 0.002 | 0.024 ± 0.002 | 0.1780 | 0.030 ± 0.005 | 0.024 ± 0.003 | 0.2491 |
| Stha_R | 0.024 ± 0.002 | 0.034 ± 0.003 | 0.0280 | 0.023 ± 0.004 | 0.034 ± 0.005 | 0.0973 |
| * SEM, standard error mean. L, left hemispheric node; R, right hemispheric node. | | | | | | |

| **Table s20. Nodal efficiency in patients with IDH mutation, intact chromosome 1p/19q, and different history of preoperative epilepsy** | | | | | | |
| --- | --- | --- | --- | --- | --- | --- |
| **Node** | Left hemispheric glioma  (mean ± SEM) | | *p* value | Right hemispheric glioma  (mean ± SEM) | | *p* value |
|  | Epilepsy | Non-epilepsy |  | Epilepsy | Non-epilepsy |  |
| A6m_L | 0.249 ± 0.024 | 0.201 ± 0.009 | 0.0781 | 0.251 ± 0.039 | 0.217 ± 0.016 | 0.4071 |
| A6m_R | 0.236 ± 0.027 | 0.202 ± 0.011 | 0.2592 | 0.263 ± 0.032 | 0.218 ± 0.017 | 0.2270 |
| A4hf_L | 0.230 ± 0.015 | 0.220 ± 0.011 | 0.5852 | 0.247 ± 0.029 | 0.232 ± 0.022 | 0.6912 |
| A4hf_R | 0.240 ± 0.023 | 0.213 ± 0.013 | 0.3166 | 0.229 ± 0.029 | 0.215 ± 0.018 | 0.6844 |
| A6cdl_L | 0.212 ± 0.016 | 0.213 ± 0.010 | 0.9560 | 0.224 ± 0.025 | 0.217 ± 0.012 | 0.7929 |
| A6cdl_R | 0.231 ± 0.022 | 0.219 ± 0.013 | 0.6469 | 0.203 ± 0.009 | 0.226 ± 0.021 | 0.3753 |
| A4ul_L | 0.244 ± 0.022 | 0.202 ± 0.010 | 0.0940 | 0.236 ± 0.019 | 0.223 ± 0.018 | 0.6447 |
| A4ul_R | 0.241 ± 0.024 | 0.220 ± 0.010 | 0.4133 | 0.254 ± 0.028 | 0.236 ± 0.017 | 0.5893 |
| A4t_L | 0.224 ± 0.017 | 0.216 ± 0.015 | 0.7052 | 0.242 ± 0.027 | 0.211 ± 0.015 | 0.3274 |
| A4t_R | 0.237 ± 0.019 | 0.219 ± 0.017 | 0.4963 | 0.241 ± 0.024 | 0.218 ± 0.012 | 0.3916 |
| A4tl_L | 0.234 ± 0.026 | 0.200 ± 0.011 | 0.2410 | 0.233 ± 0.027 | 0.199 ± 0.013 | 0.2683 |
| A4tl_R | 0.243 ± 0.023 | 0.206 ± 0.012 | 0.1683 | 0.218 ± 0.018 | 0.203 ± 0.016 | 0.5692 |
| A6cvl_L | 0.237 ± 0.021 | 0.200 ± 0.010 | 0.1231 | 0.239 ± 0.024 | 0.209 ± 0.017 | 0.3345 |
| A6cvl_R | 0.229 ± 0.020 | 0.207 ± 0.015 | 0.3886 | 0.231 ± 0.021 | 0.228 ± 0.021 | 0.9365 |
| A1/2/3ulhf_L | 0.219 ± 0.014 | 0.221 ± 0.011 | 0.9323 | 0.232 ± 0.023 | 0.250 ± 0.022 | 0.6133 |
| A1/2/3ulhf_R | 0.233 ± 0.020 | 0.218 ± 0.011 | 0.5335 | 0.255 ± 0.030 | 0.246 ± 0.023 | 0.8206 |
| A1/2/3tonIa_L | 0.257 ± 0.025 | 0.216 ± 0.014 | 0.1645 | 0.262 ± 0.038 | 0.206 ± 0.016 | 0.1731 |
| A1/2/3tonIa_R | 0.231 ± 0.012 | 0.201 ± 0.010 | 0.0548 | 0.226 ± 0.029 | 0.220 ± 0.016 | 0.8579 |
| A2_L | 0.250 ± 0.028 | 0.222 ± 0.013 | 0.3736 | 0.228 ± 0.015 | 0.218 ± 0.019 | 0.7087 |
| A2_R | 0.246 ± 0.022 | 0.213 ± 0.012 | 0.2058 | 0.235 ± 0.026 | 0.224 ± 0.020 | 0.7570 |
| A1/2/3tru_L | 0.250 ± 0.028 | 0.214 ± 0.008 | 0.2210 | 0.266 ± 0.036 | 0.222 ± 0.014 | 0.2516 |
| A1/2/3tru_R | 0.247 ± 0.028 | 0.217 ± 0.017 | 0.3738 | 0.243 ± 0.028 | 0.219 ± 0.010 | 0.4168 |
| A23d_L | 0.246 ± 0.023 | 0.207 ± 0.010 | 0.1245 | 0.227 ± 0.019 | 0.226 ± 0.015 | 0.9631 |
| A23d_R | 0.252 ± 0.002 | 0.219 ± 0.013 | 0.1616 | 0.244 ± 0.028 | 0.223 ± 0.013 | 0.4890 |
| A4ll_L | 0.214 ± 0.016 | 0.208 ± 0.013 | 0.7744 | 0.239 ± 0.026 | 0.205 ± 0.013 | 0.2408 |
| A4ll_R | 0.224 ± 0.028 | 0.215 ± 0.013 | 0.7677 | 0.235 ± 0.036 | 0.210 ± 0.012 | 0.5061 |
| A1/2/3ll_L | 0.252 ± 0.028 | 0.201 ± 0.009 | 0.0893 | 0.228 ± 0.023 | 0.215 ± 0.009 | 0.6083 |
| A1/2/3ll_R | 0.239 ± 0.026 | 0.210 ± 0.009 | 0.3149 | 0.235 ± 0.031 | 0.212 ± 0.014 | 0.4840 |
| A23v_L | 0.242 ± 0.028 | 0.220 ± 0.012 | 0.4657 | 0.256 ± 0.039 | 0.233 ± 0.017 | 0.5964 |
| A23v_R | 0.231 ± 0.025 | 0.206 ± 0.008 | 0.3555 | 0.225 ± 0.020 | 0.216 ± 0.016 | 0.7565 |
| A24cd_L | 0.243 ± 0.021 | 0.227 ± 0.013 | 0.5206 | 0.239 ± 0.028 | 0.207 ± 0.013 | 0.2899 |
| A24cd_R | 0.239 ± 0.023 | 0.234 ± 0.017 | 0.8525 | 0.236 ± 0.023 | 0.234 ± 0.021 | 0.9496 |
| A23c_L | 0.252 ± 0.028 | 0.207 ± 0.010 | 0.1519 | 0.245 ± 0.034 | 0.222 ± 0.021 | 0.5626 |
| A23c_R | 0.238 ± 0.024 | 0.204 ± 0.011 | 0.2092 | 0.263 ± 0.037 | 0.214 ± 0.017 | 0.2170 |
| A32sg_L | 0.216 ± 0.012 | 0.213 ± 0.012 | 0.9003 | 0.253 ± 0.039 | 0.218 ± 0.012 | 0.3773 |
| A32sg_R | 0.240 ± 0.025 | 0.220 ± 0.013 | 0.4948 | 0.247 ± 0.028 | 0.223 ± 0.017 | 0.4796 |
| mPMtha_L | 0.219 ± 0.023 | 0.200 ± 0.012 | 0.4620 | 0.238 ± 0.030 | 0.195 ± 0.014 | 0.1977 |
| mPMtha_R | 0.233 ± 0.022 | 0.214 ± 0.012 | 0.4595 | 0.230 ± 0.026 | 0.207 ± 0.019 | 0.4902 |
| Stha_L | 0.200 ± 0.016 | 0.204 ± 0.008 | 0.7951 | 0.247 ± 0.039 | 0.214 ± 0.016 | 0.4311 |
| Stha_R | 0.236 ± 0.024 | 0.207 ± 0.016 | 0.3251 | 0.250 ± 0.035 | 0.208 ± 0.013 | 0.2522 |
| * SEM, standard error mean. L, left hemispheric node; R, right hemispheric node. | | | | | | |

| **Table s21. Nodal local efficiency in patients with IDH mutation, intact chromosome 1p/19q, and different history of preoperative epilepsy** | | | | | | |
| --- | --- | --- | --- | --- | --- | --- |
| **Node** | Left hemispheric glioma  (mean ± SEM) | | *p* value | Right hemispheric glioma  (mean ± SEM) | | *p* value |
|  | Epilepsy | Non-epilepsy |  | Epilepsy | Non-epilepsy |  |
| A6m_L | 0.051 ± 0.006 | 0.044 ± 0.004 | 0.3628 | 0.050 ± 0.007 | 0.052 ± 0.007 | 0.8490 |
| A6m_R | 0.045 ± 0.005 | 0.048 ± 0.004 | 0.7116 | 0.042 ± 0.005 | 0.062 ± 0.014 | 0.2528 |
| A4hf_L | 0.049 ± 0.005 | 0.047 ± 0.004 | 0.7547 | 0.046 ± 0.006 | 0.053 ± 0.007 | 0.4950 |
| A4hf_R | 0.052 ± 0.004 | 0.048 ± 0.005 | 0.5565 | 0.051 ± 0.007 | 0.057 ± 0.006 | 0.5197 |
| A6cdl_L | 0.050 ± 0.006 | 0.051 ± 0.004 | 0.9102 | 0.065 ± 0.004 | 0.048 ± 0.005 | 0.0298 |
| A6cdl_R | 0.050 ± 0.005 | 0.045 ± 0.004 | 0.3993 | 0.056 ± 0.007 | 0.043 ± 0.005 | 0.1548 |
| A4ul_L | 0.050 ± 0.005 | 0.050 ± 0.004 | 0.9874 | 0.042 ± 0.007 | 0.052 ± 0.006 | 0.2673 |
| A4ul_R | 0.042 ± 0.005 | 0.045 ± 0.004 | 0.6700 | 0.038 ± 0.006 | 0.049 ± 0.006 | 0.2541 |
| A4t_L | 0.044 ± 0.005 | 0.048 ± 0.006 | 0.6494 | 0.049 ± 0.006 | 0.058 ± 0.008 | 0.4396 |
| A4t_R | 0.044 ± 0.005 | 0.047 ± 0.004 | 0.6715 | 0.056 ± 0.006 | 0.05 ± 0.005 | 0.4697 |
| A4tl_L | 0.045 ± 0.003 | 0.054 ± 0.004 | 0.1169 | 0.048 ± 0.004 | 0.052 ± 0.005 | 0.6038 |
| A4tl_R | 0.050 ± 0.004 | 0.050 ± 0.004 | 0.9740 | 0.052 ± 0.006 | 0.046 ± 0.005 | 0.5045 |
| A6cvl_L | 0.045 ± 0.005 | 0.048 ± 0.004 | 0.6709 | 0.050 ± 0.005 | 0.054 ± 0.007 | 0.7176 |
| A6cvl_R | 0.046 ± 0.005 | 0.055 ± 0.005 | 0.2016 | 0.047 ± 0.006 | 0.045 ± 0.005 | 0.8159 |
| A1/2/3ulhf_L | 0.061 ± 0.008 | 0.048 ± 0.004 | 0.1646 | 0.071 ± 0.009 | 0.052 ± 0.005 | 0.0954 |
| A1/2/3ulhf_R | 0.048 ± 0.006 | 0.053 ± 0.004 | 0.4712 | 0.054 ± 0.006 | 0.054 ± 0.007 | 0.9540 |
| A1/2/3tonIa_L | 0.048 ± 0.005 | 0.051 ± 0.004 | 0.5996 | 0.048 ± 0.006 | 0.061 ± 0.006 | 0.1499 |
| A1/2/3tonIa_R | 0.050 ± 0.005 | 0.050 ± 0.006 | 0.9074 | 0.055 ± 0.006 | 0.052 ± 0.008 | 0.7267 |
| A2_L | 0.043 ± 0.005 | 0.046 ± 0.004 | 0.6244 | 0.058 ± 0.005 | 0.044 ± 0.004 | 0.0608 |
| A2_R | 0.045 ± 0.005 | 0.053 ± 0.005 | 0.3219 | 0.050 ± 0.007 | 0.047 ± 0.006 | 0.7741 |
| A1/2/3tru_L | 0.049 ± 0.005 | 0.051 ± 0.005 | 0.8298 | 0.049 ± 0.006 | 0.053 ± 0.006 | 0.7109 |
| A1/2/3tru_R | 0.046 ± 0.005 | 0.042 ± 0.003 | 0.5028 | 0.047 ± 0.006 | 0.045 ± 0.005 | 0.7510 |
| A23d_L | 0.045 ± 0.007 | 0.047 ± 0.009 | 0.8272 | 0.043 ± 0.006 | 0.047 ± 0.005 | 0.6224 |
| A23d_R | 0.040 ± 0.004 | 0.044 ± 0.005 | 0.5662 | 0.047 ± 0.005 | 0.053 ± 0.004 | 0.3125 |
| A4ll_L | 0.042 ± 0.005 | 0.050 ± 0.006 | 0.3222 | 0.045 ± 0.007 | 0.050 ± 0.006 | 0.6195 |
| A4ll_R | 0.047 ± 0.004 | 0.043 ± 0.004 | 0.5392 | 0.045 ± 0.006 | 0.042 ± 0.006 | 0.7765 |
| A1/2/3ll_L | 0.044 ± 0.006 | 0.048 ± 0.005 | 0.6006 | 0.056 ± 0.008 | 0.043 ± 0.006 | 0.2028 |
| A1/2/3ll_R | 0.052 ± 0.005 | 0.058 ± 0.005 | 0.3932 | 0.046 ± 0.006 | 0.046 ± 0.005 | 0.9935 |
| A23v_L | 0.054 ± 0.006 | 0.046 ± 0.005 | 0.3381 | 0.045 ± 0.006 | 0.048 ± 0.004 | 0.6787 |
| A23v_R | 0.050 ± 0.005 | 0.054 ± 0.004 | 0.5178 | 0.045 ± 0.006 | 0.049 ± 0.007 | 0.7207 |
| A24cd_L | 0.047 ± 0.007 | 0.050 ± 0.003 | 0.7510 | 0.050 ± 0.004 | 0.050 ± 0.006 | 0.9550 |
| A24cd_R | 0.046 ± 0.006 | 0.046 ± 0.003 | 0.9888 | 0.050 ± 0.007 | 0.050 ± 0.007 | 0.9881 |
| A23c_L | 0.047 ± 0.004 | 0.051 ± 0.005 | 0.5065 | 0.043 ± 0.006 | 0.047 ± 0.007 | 0.6377 |
| A23c_R | 0.052 ± 0.007 | 0.051 ± 0.006 | 0.9710 | 0.046 ± 0.005 | 0.052 ± 0.004 | 0.3347 |
| A32sg_L | 0.042 ± 0.005 | 0.049 ± 0.005 | 0.3197 | 0.042 ± 0.006 | 0.048 ± 0.006 | 0.5177 |
| A32sg_R | 0.046 ± 0.005 | 0.054 ± 0.006 | 0.2999 | 0.039 ± 0.005 | 0.054 ± 0.005 | 0.0465 |
| mPMtha_L | 0.037 ± 0.004 | 0.048 ± 0.004 | 0.0357 | 0.042 ± 0.005 | 0.042 ± 0.004 | 0.9905 |
| mPMtha_R | 0.044 ± 0.004 | 0.054 ± 0.007 | 0.2873 | 0.055 ± 0.007 | 0.047 ± 0.006 | 0.3845 |
| Stha_L | 0.056 ± 0.005 | 0.048 ± 0.003 | 0.2064 | 0.055 ± 0.008 | 0.045 ± 0.006 | 0.3758 |
| Stha_R | 0.051 ± 0.007 | 0.060 ± 0.004 | 0.2516 | 0.043 ± 0.006 | 0.058 ± 0.006 | 0.0935 |
| * SEM, standard error mean. L, left hemispheric node; R, right hemispheric node. | | | | | | |

| **Table s22. Nodal vulnerability in patients with IDH mutation, intact chromosome 1p/19q, and different history of preoperative epilepsy** | | | | | | |
| --- | --- | --- | --- | --- | --- | --- |
| **Node** | Left hemispheric glioma  (mean ± SEM) | | *p* value | Right hemispheric glioma  (mean ± SEM) | | *p* value |
|  | Epilepsy | Non-epilepsy |  | Epilepsy | Non-epilepsy |  |
| A6m_L | 0.017 ± 0.005 | 0.001 ± 0.003 | 0.0166 | 0.002 ± 0.003 | 0.010 ± 0.005 | 0.2320 |
| A6m_R | 0.003 ± 0.002 | 0.002 ± 0.002 | 0.7325 | 0.024 ± 0.004 | 0.005 ± 0.008 | 0.0429 |
| A4hf_L | 0.009 ± 0.004 | 0.010 ± 0.004 | 0.9252 | 0.010 ± 0.005 | 0.013 ± 0.004 | 0.6626 |
| A4hf_R | 0.005 ± 0.002 | 0.006 ± 0.003 | 0.7889 | 0.001 ± 0.004 | 0.005 ± 0.005 | 0.4963 |
| A6cdl_L | 0.000 ± 0.002 | 0.009 ± 0.004 | 0.0585 | -0.003 ± 0.002 | 0.016 ± 0.005 | 0.0004 |
| A6cdl_R | -0.006 ± 0.004 | 0.009 ± 0.005 | 0.0283 | 0.005 ± 0.005 | 0.009 ± 0.004 | 0.5058 |
| A4ul_L | 0.011 ± 0.005 | 0.007 ± 0.003 | 0.5771 | 0.009 ± 0.004 | 0.002 ± 0.003 | 0.1954 |
| A4ul_R | 0.013 ± 0.005 | 0.005 ± 0.002 | 0.1572 | 0.013 ± 0.005 | 0.011 ± 0.004 | 0.7688 |
| A4t_L | 0.003 ± 0.003 | 0.004 ± 0.002 | 0.8587 | 0.009 ± 0.006 | 0.007 ± 0.007 | 0.8309 |
| A4t_R | 0.007 ± 0.004 | 0.011 ± 0.003 | 0.4754 | 0.006 ± 0.006 | 0.006 ± 0.003 | 0.9263 |
| A4tl_L | 0.008 ± 0.003 | 0.003 ± 0.003 | 0.2267 | 0.004 ± 0.004 | 0.002 ± 0.002 | 0.6419 |
| A4tl_R | 0.006 ± 0.003 | 0.002 ± 0.002 | 0.2472 | 0.004 ± 0.003 | 0.001 ± 0.003 | 0.3794 |
| A6cvl_L | 0.008 ± 0.003 | 0.005 ± 0.004 | 0.5549 | 0.007 ± 0.004 | 0.000 ± 0.003 | 0.1920 |
| A6cvl_R | 0.004 ± 0.003 | 0.004 ± 0.003 | 0.8987 | 0.006 ± 0.005 | 0.005 ± 0.003 | 0.7988 |
| A1/2/3ulhf_L | 0.006 ± 0.003 | 0.009 ± 0.003 | 0.5395 | 0.009 ± 0.005 | 0.018 ± 0.005 | 0.2117 |
| A1/2/3ulhf_R | 0.010 ± 0.003 | 0.009 ± 0.004 | 0.7193 | 0.018 ± 0.009 | 0.013 ± 0.005 | 0.5943 |
| A1/2/3tonIa_L | 0.013 ± 0.004 | 0.006 ± 0.003 | 0.1407 | 0.011 ± 0.004 | 0.002 ± 0.003 | 0.0907 |
| A1/2/3tonIa_R | 0.007 ± 0.003 | 0.001 ± 0.002 | 0.0803 | 0.000 ± 0.004 | 0.010 ± 0.006 | 0.2107 |
| A2_L | 0.013 ± 0.004 | 0.014 ± 0.005 | 0.8318 | 0.010 ± 0.003 | 0.005 ± 0.002 | 0.2048 |
| A2_R | 0.014 ± 0.006 | 0.011 ± 0.004 | 0.7681 | 0.007 ± 0.004 | 0.005 ± 0.003 | 0.6598 |
| A1/2/3tru_L | 0.012 ± 0.005 | 0.014 ± 0.006 | 0.7954 | 0.014 ± 0.005 | 0.006 ± 0.003 | 0.2124 |
| A1/2/3tru_R | 0.008 ± 0.005 | 0.010 ± 0.004 | 0.7865 | 0.005 ± 0.003 | 0.011 ± 0.004 | 0.3029 |
| A23d_L | 0.008 ± 0.003 | 0.003 ± 0.003 | 0.2273 | 0.004 ± 0.003 | 0.011 ± 0.006 | 0.3497 |
| A23d_R | 0.013 ± 0.004 | 0.009 ± 0.003 | 0.4819 | 0.007 ± 0.003 | 0.013 ± 0.004 | 0.3521 |
| A4ll_L | 0.002 ± 0.003 | 0.007 ± 0.003 | 0.2988 | 0.008 ± 0.004 | 0.000 ± 0.002 | 0.0665 |
| A4ll_R | -0.001 ± 0.002 | 0.004 ± 0.003 | 0.1697 | -0.003 ± 0.002 | 0.003 ± 0.003 | 0.0920 |
| A1/2/3ll_L | 0.007 ± 0.003 | 0.002 ± 0.002 | 0.1217 | 0.002 ± 0.003 | 0.013 ± 0.004 | 0.0356 |
| A1/2/3ll_R | 0.007 ± 0.003 | 0.008 ± 0.003 | 0.8577 | 0.009 ± 0.004 | 0.007 ± 0.003 | 0.7066 |
| A23v_L | 0.007 ± 0.004 | 0.010 ± 0.003 | 0.4614 | 0.007 ± 0.005 | 0.011 ± 0.004 | 0.5775 |
| A23v_R | 0.006 ± 0.004 | 0.003 ± 0.003 | 0.5168 | 0.001 ± 0.003 | 0.008 ± 0.004 | 0.1852 |
| A24cd_L | 0.007 ± 0.003 | 0.014 ± 0.004 | 0.1635 | 0.005 ± 0.004 | 0.007 ± 0.005 | 0.7273 |
| A24cd_R | 0.005 ± 0.003 | 0.018 ± 0.004 | 0.0198 | 0.007 ± 0.005 | 0.010 ± 0.003 | 0.6768 |
| A23c_L | 0.012 ± 0.004 | 0.005 ± 0.003 | 0.2111 | 0.006 ± 0.004 | 0.013 ± 0.004 | 0.2527 |
| A23c_R | 0.009 ± 0.003 | 0.009 ± 0.004 | 0.8972 | 0.013 ± 0.005 | 0.005 ± 0.004 | 0.2571 |
| A32sg_L | -0.001 ± 0.002 | 0.006 ± 0.003 | 0.0991 | 0.004 ± 0.003 | 0.010 ± 0.005 | 0.3860 |
| A32sg_R | 0.003 ± 0.002 | 0.008 ± 0.003 | 0.1614 | 0.014 ± 0.005 | 0.009 ± 0.006 | 0.5017 |
| mPMtha_L | 0.003 ± 0.005 | 0.002 ± 0.004 | 0.8920 | 0.003 ± 0.002 | -0.003 ± 0.002 | 0.0495 |
| mPMtha_R | 0.004 ± 0.003 | 0.011 ± 0.004 | 0.1345 | 0.003 ± 0.002 | 0.003 ± 0.003 | 0.8581 |
| Stha_L | -0.002 ± 0.002 | 0.006 ± 0.003 | 0.0654 | 0.003 ± 0.002 | 0.004 ± 0.003 | 0.7381 |
| Stha_R | 0.004 ± 0.003 | -0.001 ± 0.002 | 0.0979 | 0.007 ± 0.004 | 0.006 ± 0.004 | 0.8420 |
| * SEM, standard error mean. L, left hemispheric node; R, right hemispheric node. | | | | | | |

| **Table s23. Nodal betweenness in patients with IDH mutation co-deletion chromosome 1p/19q, and different history of preoperative epilepsy** | | | | | | |
| --- | --- | --- | --- | --- | --- | --- |
| **Node** | Left hemispheric glioma  (mean ± SEM) | | *p* value | Right hemispheric glioma  (mean ± SEM) | | *p* value |
|  | Epilepsy | Non-epilepsy |  | Epilepsy | Non-epilepsy |  |
| A6m_L | 14.821 ± 3.477 | 19.162 ± 3.771 | 0.4316 | 11.629 ± 3.260 | 13.662 ± 3.593 | 0.6989 |
| A6m_R | 12.372 ± 2.542 | 11.910 ± 1.865 | 0.8850 | 14.745 ± 3.296 | 19.747 ± 2.894 | 0.2847 |
| A4hf_L | 17.918 ± 3.561 | 22.035 ± 4.033 | 0.4792 | 19.885 ± 3.637 | 24.882 ± 4.737 | 0.4503 |
| A4hf_R | 17.723 ± 2.810 | 23.394 ± 3.517 | 0.2531 | 16.353 ± 3.265 | 15.179 ± 4.775 | 0.8554 |
| A6cdl_L | 13.900 ± 2.933 | 24.002 ± 5.086 | 0.1348 | 14.038 ± 3.021 | 17.813 ± 3.849 | 0.4851 |
| A6cdl_R | 11.215 ± 2.984 | 21.492 ± 4.712 | 0.1070 | 10.549 ± 4.036 | 19.280 ± 5.178 | 0.2338 |
| A4ul_L | 17.723 ± 5.261 | 23.933 ± 5.585 | 0.4505 | 20.528 ± 6.256 | 26.797 ± 6.647 | 0.5255 |
| A4ul_R | 17.195 ± 2.969 | 18.742 ± 5.688 | 0.8326 | 13.038 ± 4.382 | 23.250 ± 5.727 | 0.2067 |
| A4t_L | 25.859 ± 6.303 | 19.902 ± 6.336 | 0.5306 | 28.479 ± 6.411 | 21.852 ± 4.871 | 0.4295 |
| A4t_R | 19.818 ± 5.143 | 12.908 ± 2.037 | 0.1920 | 35.972 ± 7.333 | 19.192 ± 4.067 | 0.0548 |
| A4tl_L | 12.951 ± 2.981 | 14.654 ± 3.708 | 0.7430 | 21.049 ± 4.459 | 21.951 ± 5.357 | 0.9057 |
| A4tl_R | 20.749 ± 5.087 | 23.787 ± 4.277 | 0.6587 | 24.535 ± 6.631 | 19.967 ± 4.372 | 0.5735 |
| A6cvl_L | 15.505 ± 3.836 | 17.542 ± 3.225 | 0.6944 | 10.769 ± 2.022 | 11.448 ± 1.987 | 0.8226 |
| A6cvl_R | 24.741 ± 4.837 | 17.306 ± 2.974 | 0.1917 | 19.367 ± 3.102 | 13.766 ± 2.847 | 0.2166 |
| A1/2/3ulhf_L | 29.295 ± 6.370 | 18.658 ± 3.519 | 0.1412 | 30.962 ± 8.469 | 26.497 ± 3.835 | 0.6258 |
| A1/2/3ulhf_R | 25.549 ± 4.390 | 25.113 ± 4.401 | 0.9473 | 36.727 ± 9.117 | 12.327 ± 4.043 | 0.0190 |
| A1/2/3tonIa_L | 18.546 ± 3.911 | 13.479 ± 2.950 | 0.3131 | 15.101 ± 5.397 | 21.445 ± 7.101 | 0.5208 |
| A1/2/3tonIa_R | 17.972 ± 4.880 | 17.738 ± 3.297 | 0.9685 | 11.958 ± 3.350 | 26.203 ± 7.232 | 0.1292 |
| A2_L | 18.746 ± 4.488 | 26.131 ± 4.991 | 0.3098 | 16.322 ± 5.000 | 23.253 ± 4.757 | 0.3494 |
| A2_R | 25.031 ± 4.591 | 25.183 ± 4.454 | 0.9820 | 23.941 ± 5.351 | 15.071 ± 3.752 | 0.1939 |
| A1/2/3tru_L | 24.382 ± 4.674 | 20.835 ± 4.133 | 0.5857 | 37.136 ± 7.928 | 22.802 ± 4.595 | 0.1290 |
| A1/2/3tru_R | 18.664 ± 4.402 | 22.075 ± 3.446 | 0.5519 | 19.458 ± 8.407 | 16.343 ± 2.965 | 0.7179 |
| A23d_L | 25.241 ± 4.897 | 26.477 ± 4.066 | 0.8507 | 24.126 ± 6.063 | 18.555 ± 3.405 | 0.4269 |
| A23d_R | 21.038 ± 4.416 | 27.488 ± 4.095 | 0.3106 | 17.346 ± 5.311 | 28.170 ± 4.689 | 0.1561 |
| A4ll_L | 10.223 ± 2.356 | 9.602 ± 2.750 | 0.8740 | 14.255 ± 3.139 | 7.091 ± 2.709 | 0.1099 |
| A4ll_R | 11.985 ± 3.637 | 12.254 ± 2.378 | 0.9505 | 18.455 ± 4.940 | 9.536 ± 3.429 | 0.1567 |
| A1/2/3ll_L | 13.872 ± 2.707 | 16.531 ± 2.827 | 0.5251 | 17.017 ± 4.170 | 17.368 ± 4.494 | 0.9577 |
| A1/2/3ll_R | 27.069 ± 6.249 | 26.094 ± 4.135 | 0.8964 | 24.014 ± 4.362 | 33.066 ± 7.646 | 0.3679 |
| A23v_L | 26.408 ± 4.609 | 34.131 ± 5.405 | 0.3188 | 9.045 ± 2.647 | 22.352 ± 3.855 | 0.0166 |
| A23v_R | 22.290 ± 3.911 | 15.204 ± 3.398 | 0.1935 | 18.311 ± 5.450 | 23.088 ± 4.894 | 0.5385 |
| A24cd_L | 26.769 ± 5.691 | 21.981 ± 4.422 | 0.5169 | 26.192 ± 5.180 | 34.231 ± 7.089 | 0.4117 |
| A24cd_R | 25.285 ± 4.467 | 23.096 ± 4.300 | 0.7378 | 20.699 ± 4.418 | 29.505 ± 6.474 | 0.3187 |
| A23c_L | 17.236 ± 2.984 | 19.660 ± 3.832 | 0.6491 | 15.287 ± 3.346 | 14.217 ± 3.613 | 0.8405 |
| A23c_R | 20.828 ± 3.782 | 20.362 ± 3.952 | 0.9362 | 28.115 ± 7.275 | 15.931 ± 3.512 | 0.1362 |
| A32sg_L | 24.541 ± 3.597 | 19.175 ± 4.119 | 0.3661 | 20.112 ± 5.862 | 18.552 ± 4.613 | 0.8406 |
| A32sg_R | 25.531 ± 3.522 | 21.896 ± 2.775 | 0.4307 | 21.126 ± 5.647 | 18.008 ± 3.602 | 0.6471 |
| mPMtha_L | 16.467 ± 4.131 | 10.829 ± 2.907 | 0.2725 | 6.021 ± 1.592 | 13.302 ± 2.079 | 0.0178 |
| mPMtha_R | 27.595 ± 9.907 | 15.873 ± 4.785 | 0.2728 | 18.871 ± 3.586 | 15.379 ± 3.963 | 0.5475 |
| Stha_L | 8.485 ± 2.723 | 23.681 ± 3.722 | 0.0050 | 11.290 ± 4.391 | 10.981 ± 2.998 | 0.9546 |
| Stha_R | 20.795 ± 3.823 | 11.144 ± 3.361 | 0.0752 | 11.804 ± 2.637 | 24.621 ± 4.357 | 0.0337 |
| * SEM, standard error mean. L, left hemispheric node; R, right hemispheric node. | | | | | | |

| **Table s24. Nodal degree centrality in patients with IDH mutation, co-deletion chromosome 1p/19q, and different history of preoperative epilepsy** | | | | | | |
| --- | --- | --- | --- | --- | --- | --- |
| **Node** | Left hemispheric glioma  (mean ± SEM) | | *p* value | Right hemispheric glioma  (mean ± SEM) | | *p* value |
|  | Epilepsy | Non-epilepsy |  | Epilepsy | Non-epilepsy |  |
| A6m_L | 3.232 ± 0.189 | 3.037 ± 0.245 | 0.5644 | 5.139 ± 1.166 | 3.432 ± 0.711 | 0.2235 |
| A6m_R | 3.222 ± 0.309 | 2.868 ± 0.114 | 0.2574 | 5.269 ± 1.329 | 3.705 ± 0.548 | 0.2710 |
| A4hf_L | 3.822 ± 0.319 | 3.377 ± 0.207 | 0.2452 | 5.919 ± 1.431 | 3.847 ± 0.602 | 0.1805 |
| A4hf_R | 3.630 ± 0.472 | 3.187 ± 0.193 | 0.3618 | 4.829 ± 1.189 | 3.458 ± 0.631 | 0.3119 |
| A6cdl_L | 3.247 ± 0.319 | 3.000 ± 0.242 | 0.5454 | 3.678 ± 0.627 | 3.534 ± 0.707 | 0.8881 |
| A6cdl_R | 3.060 ± 0.375 | 3.241 ± 0.261 | 0.6945 | 3.866 ± 0.634 | 3.768 ± 0.603 | 0.9157 |
| A4ul_L | 2.951 ± 0.353 | 3.130 ± 0.215 | 0.6611 | 4.200 ± 1.096 | 3.226 ± 0.304 | 0.3739 |
| A4ul_R | 3.272 ± 0.317 | 3.135 ± 0.208 | 0.7184 | 3.784 ± 0.823 | 3.415 ± 0.274 | 0.6594 |
| A4t_L | 3.512 ± 0.428 | 3.013 ± 0.242 | 0.3036 | 4.697 ± 0.568 | 3.956 ± 0.588 | 0.4024 |
| A4t_R | 3.720 ± 0.636 | 2.839 ± 0.142 | 0.1463 | 6.475 ± 1.599 | 3.791 ± 0.583 | 0.1131 |
| A4tl_L | 3.310 ± 0.309 | 2.898 ± 0.223 | 0.2888 | 5.536 ± 0.990 | 3.823 ± 0.537 | 0.1369 |
| A4tl_R | 3.442 ± 0.288 | 3.333 ± 0.206 | 0.7603 | 6.162 ± 1.423 | 3.837 ± 0.706 | 0.1487 |
| A6cvl_L | 3.008 ± 0.265 | 2.781 ± 0.163 | 0.4623 | 5.006 ± 1.045 | 3.058 ± 0.429 | 0.0871 |
| A6cvl_R | 3.919 ± 0.540 | 3.038 ± 0.195 | 0.1084 | 4.805 ± 0.951 | 3.226 ± 0.422 | 0.1312 |
| A1/2/3ulhf_L | 3.900 ± 0.248 | 3.070 ± 0.191 | 0.0132 | 5.240 ± 1.046 | 3.862 ± 0.254 | 0.1863 |
| A1/2/3ulhf_R | 3.924 ± 0.279 | 3.340 ± 0.188 | 0.0901 | 5.581 ± 1.245 | 3.419 ± 0.615 | 0.1255 |
| A1/2/3tonIa_L | 3.479 ± 0.455 | 2.892 ± 0.193 | 0.2164 | 4.241 ± 1.088 | 3.603 ± 0.576 | 0.6039 |
| A1/2/3tonIa_R | 3.453 ± 0.391 | 2.946 ± 0.165 | 0.2144 | 4.095 ± 0.635 | 3.557 ± 0.451 | 0.5024 |
| A2_L | 3.625 ± 0.391 | 3.406 ± 0.213 | 0.6147 | 3.684 ± 0.674 | 3.391 ± 0.277 | 0.6806 |
| A2_R | 3.616 ± 0.366 | 3.537 ± 0.209 | 0.8491 | 5.177 ± 1.314 | 3.333 ± 0.621 | 0.2061 |
| A1/2/3tru_L | 3.411 ± 0.284 | 3.028 ± 0.231 | 0.3124 | 6.037 ± 1.105 | 3.873 ± 0.565 | 0.0889 |
| A1/2/3tru_R | 3.741 ± 0.445 | 3.265 ± 0.155 | 0.2839 | 4.074 ± 0.793 | 3.289 ± 0.487 | 0.4073 |
| A23d_L | 3.844 ± 0.325 | 3.154 ± 0.234 | 0.0947 | 4.392 ± 0.524 | 3.423 ± 0.487 | 0.2093 |
| A23d_R | 3.455 ± 0.319 | 3.388 ± 0.227 | 0.8647 | 4.754 ± 1.081 | 3.871 ± 0.527 | 0.4602 |
| A4ll_L | 3.233 ± 0.430 | 2.732 ± 0.219 | 0.2867 | 3.998 ± 0.816 | 2.871 ± 0.672 | 0.3125 |
| A4ll_R | 3.453 ± 0.495 | 2.829 ± 0.160 | 0.2026 | 4.916 ± 0.830 | 2.631 ± 0.213 | 0.0094 |
| A1/2/3ll_L | 3.347 ± 0.238 | 3.041 ± 0.157 | 0.2863 | 5.524 ± 1.385 | 2.518 ± 0.219 | 0.0307 |
| A1/2/3ll_R | 3.616 ± 0.214 | 3.421 ± 0.165 | 0.4811 | 5.926 ± 1.537 | 3.316 ± 0.348 | 0.0908 |
| A23v_L | 3.903 ± 0.472 | 3.544 ± 0.237 | 0.4830 | 4.864 ± 1.289 | 3.555 ± 0.475 | 0.3305 |
| A23v_R | 3.648 ± 0.432 | 2.720 ± 0.214 | 0.0527 | 3.989 ± 0.730 | 3.178 ± 0.305 | 0.2996 |
| A24cd_L | 3.717 ± 0.356 | 3.161 ± 0.204 | 0.1734 | 4.370 ± 0.668 | 4.266 ± 0.631 | 0.9153 |
| A24cd_R | 4.305 ± 0.559 | 3.275 ± 0.218 | 0.0761 | 4.384 ± 0.574 | 4.050 ± 0.716 | 0.7400 |
| A23c_L | 3.289 ± 0.181 | 3.108 ± 0.206 | 0.5400 | 3.800 ± 0.734 | 3.223 ± 0.475 | 0.5177 |
| A23c_R | 3.837 ± 0.364 | 2.937 ± 0.193 | 0.0302 | 5.471 ± 1.115 | 3.488 ± 0.665 | 0.1390 |
| A32sg_L | 3.972 ± 0.507 | 2.990 ± 0.185 | 0.0598 | 4.029 ± 1.029 | 3.153 ± 0.180 | 0.3778 |
| A32sg_R | 3.714 ± 0.384 | 3.265 ± 0.132 | 0.2418 | 4.993 ± 0.786 | 2.891 ± 0.285 | 0.0151 |
| mPMtha_L | 3.075 ± 0.316 | 2.654 ± 0.192 | 0.2545 | 3.743 ± 0.785 | 3.581 ± 0.610 | 0.8752 |
| mPMtha_R | 4.100 ± 0.705 | 2.839 ± 0.181 | 0.0664 | 4.421 ± 0.625 | 3.521 ± 0.687 | 0.3741 |
| Stha_L | 2.909 ± 0.362 | 3.417 ± 0.207 | 0.2188 | 4.536 ± 1.288 | 2.957 ± 0.389 | 0.2283 |
| Stha_R | 4.118 ± 0.466 | 2.600 ± 0.173 | 0.0025 | 3.269 ± 0.343 | 3.161 ± 0.398 | 0.8507 |
| * SEM, standard error mean. L, left hemispheric node; R, right hemispheric node. | | | | | | |

| **Table s25. Nodal clustering coefficient in patients with IDH mutation, co-deletion chromosome 1p/19q, and different history of preoperative epilepsy** | | | | | | |
| --- | --- | --- | --- | --- | --- | --- |
| **Node** | Left hemispheric glioma  (mean ± SEM) | | *p* value | Right hemispheric glioma  (mean ± SEM) | | *p* value |
|  | Epilepsy | Non-epilepsy |  | Epilepsy | Non-epilepsy |  |
| A6m_L | 0.021 ± 0.003 | 0.028 ± 0.003 | 0.1006 | 0.021 ± 0.005 | 0.026 ± 0.004 | 0.4208 |
| A6m_R | 0.028 ± 0.004 | 0.029 ± 0.002 | 0.9192 | 0.028 ± 0.008 | 0.025 ± 0.003 | 0.7947 |
| A4hf_L | 0.024 ± 0.003 | 0.027 ± 0.003 | 0.4791 | 0.019 ± 0.004 | 0.027 ± 0.004 | 0.2195 |
| A4hf_R | 0.025 ± 0.003 | 0.025 ± 0.002 | 0.9238 | 0.018 ± 0.005 | 0.029 ± 0.005 | 0.1400 |
| A6cdl_L | 0.025 ± 0.003 | 0.031 ± 0.004 | 0.2843 | 0.019 ± 0.004 | 0.029 ± 0.004 | 0.0890 |
| A6cdl_R | 0.026 ± 0.004 | 0.028 ± 0.003 | 0.7034 | 0.020 ± 0.004 | 0.031 ± 0.004 | 0.0681 |
| A4ul_L | 0.023 ± 0.002 | 0.025 ± 0.003 | 0.6650 | 0.021 ± 0.004 | 0.025 ± 0.003 | 0.4894 |
| A4ul_R | 0.027 ± 0.004 | 0.032 ± 0.003 | 0.3688 | 0.018 ± 0.003 | 0.027 ± 0.004 | 0.1496 |
| A4t_L | 0.028 ± 0.004 | 0.027 ± 0.003 | 0.9037 | 0.015 ± 0.003 | 0.025 ± 0.004 | 0.0619 |
| A4t_R | 0.030 ± 0.006 | 0.024 ± 0.002 | 0.3124 | 0.018 ± 0.004 | 0.025 ± 0.003 | 0.2073 |
| A4tl_L | 0.024 ± 0.003 | 0.030 ± 0.003 | 0.2170 | 0.018 ± 0.004 | 0.024 ± 0.003 | 0.2170 |
| A4tl_R | 0.029 ± 0.004 | 0.026 ± 0.002 | 0.6060 | 0.018 ± 0.004 | 0.024 ± 0.003 | 0.2226 |
| A6cvl_L | 0.028 ± 0.004 | 0.022 ± 0.002 | 0.1711 | 0.018 ± 0.004 | 0.042 ± 0.009 | 0.0554 |
| A6cvl_R | 0.027 ± 0.004 | 0.029 ± 0.003 | 0.7263 | 0.018 ± 0.004 | 0.028 ± 0.004 | 0.1043 |
| A1/2/3ulhf_L | 0.024 ± 0.003 | 0.028 ± 0.003 | 0.3570 | 0.015 ± 0.003 | 0.025 ± 0.003 | 0.0247 |
| A1/2/3ulhf_R | 0.027 ± 0.004 | 0.027 ± 0.002 | 0.9856 | 0.015 ± 0.004 | 0.026 ± 0.003 | 0.0155 |
| A1/2/3tonIa_L | 0.027 ± 0.004 | 0.024 ± 0.003 | 0.5037 | 0.023 ± 0.006 | 0.029 ± 0.005 | 0.5044 |
| A1/2/3tonIa_R | 0.025 ± 0.003 | 0.026 ± 0.003 | 0.7168 | 0.020 ± 0.005 | 0.026 ± 0.003 | 0.3315 |
| A2_L | 0.027 ± 0.003 | 0.025 ± 0.002 | 0.6580 | 0.018 ± 0.004 | 0.029 ± 0.004 | 0.0490 |
| A2_R | 0.025 ± 0.003 | 0.027 ± 0.002 | 0.7147 | 0.019 ± 0.003 | 0.025 ± 0.004 | 0.2594 |
| A1/2/3tru_L | 0.026 ± 0.004 | 0.026 ± 0.003 | 0.9682 | 0.018 ± 0.005 | 0.027 ± 0.005 | 0.2205 |
| A1/2/3tru_R | 0.028 ± 0.003 | 0.031 ± 0.004 | 0.6411 | 0.023 ± 0.006 | 0.029 ± 0.004 | 0.4210 |
| A23d_L | 0.022 ± 0.003 | 0.029 ± 0.005 | 0.2978 | 0.013 ± 0.002 | 0.027 ± 0.003 | 0.0083 |
| A23d_R | 0.025 ± 0.003 | 0.026 ± 0.004 | 0.8078 | 0.019 ± 0.003 | 0.025 ± 0.003 | 0.2933 |
| A4ll_L | 0.020 ± 0.003 | 0.028 ± 0.003 | 0.0846 | 0.019 ± 0.004 | 0.032 ± 0.004 | 0.0332 |
| A4ll_R | 0.024 ± 0.003 | 0.027 ± 0.003 | 0.4768 | 0.016 ± 0.003 | 0.030 ± 0.004 | 0.0198 |
| A1/2/3ll_L | 0.025 ± 0.003 | 0.028 ± 0.003 | 0.4092 | 0.017 ± 0.004 | 0.026 ± 0.004 | 0.1171 |
| A1/2/3ll_R | 0.027 ± 0.003 | 0.029 ± 0.002 | 0.4279 | 0.024 ± 0.005 | 0.028 ± 0.004 | 0.5528 |
| A23v_L | 0.024 ± 0.003 | 0.023 ± 0.003 | 0.7749 | 0.021 ± 0.004 | 0.026 ± 0.005 | 0.4420 |
| A23v_R | 0.024 ± 0.002 | 0.028 ± 0.003 | 0.3458 | 0.016 ± 0.003 | 0.023 ± 0.003 | 0.1464 |
| A24cd_L | 0.023 ± 0.003 | 0.031 ± 0.003 | 0.0851 | 0.019 ± 0.003 | 0.025 ± 0.002 | 0.1361 |
| A24cd_R | 0.026 ± 0.003 | 0.032 ± 0.003 | 0.2035 | 0.019 ± 0.003 | 0.029 ± 0.005 | 0.1177 |
| A23c_L | 0.029 ± 0.004 | 0.030 ± 0.003 | 0.7100 | 0.017 ± 0.003 | 0.027 ± 0.003 | 0.0531 |
| A23c_R | 0.023 ± 0.002 | 0.032 ± 0.004 | 0.0781 | 0.018 ± 0.005 | 0.031 ± 0.006 | 0.1403 |
| A32sg_L | 0.023 ± 0.003 | 0.027 ± 0.002 | 0.2807 | 0.016 ± 0.004 | 0.024 ± 0.003 | 0.1226 |
| A32sg_R | 0.024 ± 0.003 | 0.026 ± 0.002 | 0.4894 | 0.018 ± 0.003 | 0.027 ± 0.005 | 0.1630 |
| mPMtha_L | 0.021 ± 0.003 | 0.025 ± 0.003 | 0.3000 | 0.020 ± 0.004 | 0.017 ± 0.002 | 0.5627 |
| mPMtha_R | 0.027 ± 0.005 | 0.028 ± 0.002 | 0.7521 | 0.018 ± 0.004 | 0.028 ± 0.004 | 0.0932 |
| Stha_L | 0.021 ± 0.003 | 0.031 ± 0.003 | 0.0432 | 0.014 ± 0.002 | 0.031 ± 0.004 | 0.0020 |
| Stha_R | 0.016 ± 0.003 | 0.025 ± 0.002 | 0.0161 | 0.019 ± 0.005 | 0.028 ± 0.003 | 0.1812 |
| * SEM, standard error mean. L, left hemispheric node; R, right hemispheric node. | | | | | | |

| **Table s26. Nodal efficiency in patients with IDH mutation, co-deletion chromosome 1p/19q, and different history of preoperative epilepsy** | | | | | | |
| --- | --- | --- | --- | --- | --- | --- |
| **Node** | Left hemispheric glioma  (mean ± SEM) | | *p* value | Right hemispheric glioma  (mean ± SEM) | | *p* value |
|  | Epilepsy | Non-epilepsy |  | Epilepsy | Non-epilepsy |  |
| A6m_L | 0.222 ± 0.012 | 0.203 ± 0.008 | 0.1761 | 0.307 ± 0.052 | 0.216 ± 0.025 | 0.1216 |
| A6m_R | 0.220 ± 0.017 | 0.197 ± 0.005 | 0.1570 | 0.298 ± 0.053 | 0.225 ± 0.021 | 0.1941 |
| A4hf_L | 0.244 ± 0.018 | 0.213 ± 0.007 | 0.1076 | 0.327 ± 0.056 | 0.236 ± 0.021 | 0.1246 |
| A4hf_R | 0.237 ± 0.020 | 0.213 ± 0.008 | 0.2331 | 0.301 ± 0.054 | 0.221 ± 0.023 | 0.1754 |
| A6cdl_L | 0.224 ± 0.016 | 0.204 ± 0.008 | 0.2538 | 0.240 ± 0.028 | 0.221 ± 0.025 | 0.6241 |
| A6cdl_R | 0.217 ± 0.019 | 0.204 ± 0.009 | 0.5194 | 0.273 ± 0.035 | 0.223 ± 0.023 | 0.2447 |
| A4ul_L | 0.236 ± 0.016 | 0.198 ± 0.007 | 0.0318 | 0.272 ± 0.046 | 0.202 ± 0.017 | 0.1423 |
| A4ul_R | 0.211 ± 0.014 | 0.207 ± 0.007 | 0.7756 | 0.283 ± 0.036 | 0.194 ± 0.009 | 0.0186 |
| A4t_L | 0.235 ± 0.019 | 0.197 ± 0.007 | 0.0521 | 0.288 ± 0.033 | 0.236 ± 0.022 | 0.2037 |
| A4t_R | 0.237 ± 0.026 | 0.196 ± 0.006 | 0.1041 | 0.341 ± 0.062 | 0.227 ± 0.021 | 0.0842 |
| A4tl_L | 0.221 ± 0.017 | 0.197 ± 0.008 | 0.1944 | 0.324 ± 0.050 | 0.229 ± 0.020 | 0.0823 |
| A4tl_R | 0.234 ± 0.015 | 0.211 ± 0.008 | 0.1636 | 0.339 ± 0.058 | 0.230 ± 0.025 | 0.0872 |
| A6cvl_L | 0.215 ± 0.013 | 0.195 ± 0.006 | 0.1462 | 0.304 ± 0.050 | 0.205 ± 0.017 | 0.0633 |
| A6cvl_R | 0.244 ± 0.023 | 0.203 ± 0.007 | 0.0797 | 0.296 ± 0.046 | 0.210 ± 0.018 | 0.0805 |
| A1/2/3ulhf_L | 0.243 ± 0.015 | 0.208 ± 0.007 | 0.0334 | 0.304 ± 0.042 | 0.232 ± 0.012 | 0.0980 |
| A1/2/3ulhf_R | 0.241 ± 0.014 | 0.213 ± 0.007 | 0.0727 | 0.316 ± 0.047 | 0.215 ± 0.023 | 0.0594 |
| A1/2/3tonIa_L | 0.237 ± 0.023 | 0.198 ± 0.007 | 0.0798 | 0.272 ± 0.048 | 0.220 ± 0.021 | 0.3177 |
| A1/2/3tonIa_R | 0.232 ± 0.019 | 0.203 ± 0.005 | 0.1207 | 0.273 ± 0.037 | 0.221 ± 0.018 | 0.2091 |
| A2_L | 0.237 ± 0.019 | 0.212 ± 0.008 | 0.1992 | 0.259 ± 0.035 | 0.214 ± 0.013 | 0.2174 |
| A2_R | 0.236 ± 0.017 | 0.215 ± 0.008 | 0.2333 | 0.297 ± 0.049 | 0.215 ± 0.023 | 0.1333 |
| A1/2/3tru_L | 0.230 ± 0.011 | 0.203 ± 0.009 | 0.0898 | 0.334 ± 0.051 | 0.229 ± 0.021 | 0.0603 |
| A1/2/3tru_R | 0.239 ± 0.022 | 0.208 ± 0.006 | 0.1537 | 0.271 ± 0.036 | 0.214 ± 0.020 | 0.1776 |
| A23d_L | 0.243 ± 0.015 | 0.210 ± 0.009 | 0.0627 | 0.282 ± 0.029 | 0.218 ± 0.019 | 0.0784 |
| A23d_R | 0.233 ± 0.018 | 0.216 ± 0.008 | 0.3786 | 0.303 ± 0.050 | 0.239 ± 0.019 | 0.2297 |
| A4ll_L | 0.221 ± 0.020 | 0.192 ± 0.008 | 0.1587 | 0.275 ± 0.039 | 0.199 ± 0.025 | 0.1166 |
| A4ll_R | 0.231 ± 0.025 | 0.198 ± 0.006 | 0.1747 | 0.307 ± 0.043 | 0.196 ± 0.011 | 0.0148 |
| A1/2/3ll_L | 0.226 ± 0.013 | 0.203 ± 0.006 | 0.0923 | 0.324 ± 0.061 | 0.185 ± 0.008 | 0.0221 |
| A1/2/3ll_R | 0.234 ± 0.012 | 0.214 ± 0.006 | 0.1280 | 0.331 ± 0.060 | 0.200 ± 0.016 | 0.0364 |
| A23v_L | 0.246 ± 0.023 | 0.219 ± 0.008 | 0.2344 | 0.293 ± 0.060 | 0.230 ± 0.019 | 0.3013 |
| A23v_R | 0.232 ± 0.017 | 0.199 ± 0.008 | 0.0780 | 0.271 ± 0.035 | 0.198 ± 0.015 | 0.0637 |
| A24cd_L | 0.239 ± 0.018 | 0.207 ± 0.008 | 0.0875 | 0.266 ± 0.026 | 0.242 ± 0.024 | 0.5307 |
| A24cd_R | 0.252 ± 0.024 | 0.211 ± 0.008 | 0.0920 | 0.290 ± 0.033 | 0.235 ± 0.026 | 0.2139 |
| A23c_L | 0.226 ± 0.013 | 0.203 ± 0.008 | 0.1472 | 0.252 ± 0.040 | 0.214 ± 0.019 | 0.3843 |
| A23c_R | 0.242 ± 0.018 | 0.201 ± 0.008 | 0.0348 | 0.313 ± 0.051 | 0.217 ± 0.023 | 0.0887 |
| A32sg_L | 0.249 ± 0.020 | 0.204 ± 0.007 | 0.0354 | 0.258 ± 0.047 | 0.215 ± 0.010 | 0.3477 |
| A32sg_R | 0.244 ± 0.018 | 0.211 ± 0.004 | 0.0637 | 0.302 ± 0.041 | 0.194 ± 0.016 | 0.0179 |
| mPMtha_L | 0.221 ± 0.017 | 0.187 ± 0.007 | 0.0679 | 0.257 ± 0.041 | 0.220 ± 0.024 | 0.4424 |
| mPMtha_R | 0.246 ± 0.027 | 0.195 ± 0.007 | 0.0544 | 0.285 ± 0.034 | 0.212 ± 0.025 | 0.1116 |
| Stha_L | 0.213 ± 0.019 | 0.209 ± 0.007 | 0.8291 | 0.310 ± 0.047 | 0.202 ± 0.018 | 0.0362 |
| Stha_R | 0.255 ± 0.023 | 0.186 ± 0.007 | 0.0041 | 0.239 ± 0.017 | 0.208 ± 0.017 | 0.2304 |
| * SEM, standard error mean. L, left hemispheric node; R, right hemispheric node. | | | | | | |

| **Table s27. Nodal local efficiency in patients with IDH mutation, co-deletion chromosome 1p/19q, and different history of preoperative epilepsy** | | | | | | |
| --- | --- | --- | --- | --- | --- | --- |
| **Node** | Left hemispheric glioma  (mean ± SEM) | | *p* value | Right hemispheric glioma  (mean ± SEM) | | *p* value |
|  | Epilepsy | Non-epilepsy |  | Epilepsy | Non-epilepsy |  |
| A6m_L | 0.041 ± 0.005 | 0.047 ± 0.004 | 0.4218 | 0.039 ± 0.008 | 0.052 ± 0.007 | 0.2830 |
| A6m_R | 0.054 ± 0.007 | 0.054 ± 0.004 | 0.9794 | 0.050 ± 0.011 | 0.043 ± 0.005 | 0.5949 |
| A4hf_L | 0.046 ± 0.005 | 0.050 ± 0.005 | 0.5400 | 0.035 ± 0.006 | 0.044 ± 0.006 | 0.3104 |
| A4hf_R | 0.050 ± 0.007 | 0.045 ± 0.004 | 0.5915 | 0.035 ± 0.008 | 0.048 ± 0.007 | 0.2398 |
| A6cdl_L | 0.046 ± 0.004 | 0.050 ± 0.005 | 0.5848 | 0.038 ± 0.006 | 0.049 ± 0.005 | 0.1830 |
| A6cdl_R | 0.049 ± 0.007 | 0.050 ± 0.006 | 0.9895 | 0.037 ± 0.006 | 0.059 ± 0.007 | 0.0393 |
| A4ul_L | 0.044 ± 0.003 | 0.045 ± 0.005 | 0.8809 | 0.045 ± 0.009 | 0.047 ± 0.007 | 0.8228 |
| A4ul_R | 0.052 ± 0.008 | 0.057 ± 0.005 | 0.5660 | 0.041 ± 0.008 | 0.048 ± 0.008 | 0.5278 |
| A4t_L | 0.051 ± 0.006 | 0.046 ± 0.004 | 0.5010 | 0.035 ± 0.007 | 0.046 ± 0.007 | 0.2928 |
| A4t_R | 0.053 ± 0.008 | 0.047 ± 0.004 | 0.4704 | 0.035 ± 0.008 | 0.046 ± 0.006 | 0.3168 |
| A4tl_L | 0.048 ± 0.006 | 0.057 ± 0.006 | 0.3074 | 0.033 ± 0.006 | 0.045 ± 0.006 | 0.1787 |
| A4tl_R | 0.050 ± 0.005 | 0.049 ± 0.004 | 0.8198 | 0.035 ± 0.008 | 0.041 ± 0.005 | 0.5149 |
| A6cvl_L | 0.054 ± 0.006 | 0.042 ± 0.003 | 0.0796 | 0.036 ± 0.008 | 0.067 ± 0.010 | 0.0431 |
| A6cvl_R | 0.050 ± 0.007 | 0.055 ± 0.006 | 0.5762 | 0.036 ± 0.008 | 0.050 ± 0.008 | 0.2366 |
| A1/2/3ulhf_L | 0.047 ± 0.005 | 0.049 ± 0.004 | 0.8334 | 0.027 ± 0.005 | 0.045 ± 0.004 | 0.0228 |
| A1/2/3ulhf_R | 0.051 ± 0.007 | 0.050 ± 0.004 | 0.8815 | 0.027 ± 0.005 | 0.052 ± 0.006 | 0.0074 |
| A1/2/3tonIa_L | 0.052 ± 0.005 | 0.046 ± 0.005 | 0.4590 | 0.043 ± 0.010 | 0.052 ± 0.008 | 0.5084 |
| A1/2/3tonIa_R | 0.046 ± 0.006 | 0.045 ± 0.005 | 0.9151 | 0.038 ± 0.007 | 0.045 ± 0.006 | 0.4476 |
| A2_L | 0.049 ± 0.005 | 0.045 ± 0.004 | 0.5889 | 0.034 ± 0.009 | 0.052 ± 0.006 | 0.1033 |
| A2_R | 0.047 ± 0.005 | 0.052 ± 0.006 | 0.5075 | 0.045 ± 0.007 | 0.045 ± 0.006 | 0.9811 |
| A1/2/3tru_L | 0.050 ± 0.007 | 0.046 ± 0.007 | 0.6807 | 0.034 ± 0.008 | 0.050 ± 0.007 | 0.1609 |
| A1/2/3tru_R | 0.053 ± 0.005 | 0.057 ± 0.006 | 0.6284 | 0.049 ± 0.010 | 0.053 ± 0.006 | 0.7331 |
| A23d_L | 0.042 ± 0.006 | 0.050 ± 0.006 | 0.3547 | 0.030 ± 0.005 | 0.047 ± 0.006 | 0.0579 |
| A23d_R | 0.047 ± 0.005 | 0.046 ± 0.006 | 0.8974 | 0.044 ± 0.008 | 0.038 ± 0.004 | 0.4960 |
| A4ll_L | 0.039 ± 0.006 | 0.053 ± 0.007 | 0.1486 | 0.036 ± 0.008 | 0.061 ± 0.007 | 0.0366 |
| A4ll_R | 0.044 ± 0.006 | 0.052 ± 0.006 | 0.3535 | 0.032 ± 0.007 | 0.051 ± 0.006 | 0.0672 |
| A1/2/3ll_L | 0.051 ± 0.006 | 0.054 ± 0.006 | 0.7319 | 0.036 ± 0.009 | 0.050 ± 0.006 | 0.2141 |
| A1/2/3ll_R | 0.057 ± 0.006 | 0.053 ± 0.003 | 0.5486 | 0.049 ± 0.009 | 0.049 ± 0.006 | 0.9806 |
| A23v_L | 0.043 ± 0.006 | 0.041 ± 0.004 | 0.7504 | 0.044 ± 0.009 | 0.043 ± 0.006 | 0.9766 |
| A23v_R | 0.046 ± 0.004 | 0.052 ± 0.006 | 0.4673 | 0.031 ± 0.006 | 0.039 ± 0.005 | 0.3121 |
| A24cd_L | 0.051 ± 0.006 | 0.054 ± 0.006 | 0.7262 | 0.041 ± 0.006 | 0.050 ± 0.007 | 0.3674 |
| A24cd_R | 0.047 ± 0.005 | 0.056 ± 0.006 | 0.2859 | 0.040 ± 0.006 | 0.050 ± 0.008 | 0.4325 |
| A23c_L | 0.052 ± 0.006 | 0.058 ± 0.006 | 0.5419 | 0.036 ± 0.007 | 0.049 ± 0.007 | 0.2338 |
| A23c_R | 0.047 ± 0.004 | 0.059 ± 0.006 | 0.1310 | 0.037 ± 0.008 | 0.051 ± 0.008 | 0.2560 |
| A32sg_L | 0.043 ± 0.004 | 0.047 ± 0.004 | 0.5434 | 0.028 ± 0.006 | 0.042 ± 0.006 | 0.1360 |
| A32sg_R | 0.044 ± 0.007 | 0.049 ± 0.003 | 0.5111 | 0.036 ± 0.008 | 0.047 ± 0.006 | 0.3036 |
| mPMtha_L | 0.039 ± 0.005 | 0.046 ± 0.005 | 0.3618 | 0.040 ± 0.009 | 0.030 ± 0.004 | 0.3079 |
| mPMtha_R | 0.045 ± 0.006 | 0.055 ± 0.005 | 0.2135 | 0.033 ± 0.006 | 0.052 ± 0.006 | 0.0393 |
| Stha_L | 0.047 ± 0.006 | 0.037 ± 0.004 | 0.1684 | 0.038 ± 0.007 | 0.051 ± 0.007 | 0.2164 |
| Stha_R | 0.040 ± 0.006 | 0.055 ± 0.005 | 0.0845 | 0.032 ± 0.005 | 0.054 ± 0.006 | 0.0177 |
| * SEM, standard error mean. L, left hemispheric node; R, right hemispheric node. | | | | | | |

| **Table s28. Nodal vulnerability in patients with IDH mutation, co-deletion chromosome 1p/19q, and different history of preoperative epilepsy** | | | | | | |
| --- | --- | --- | --- | --- | --- | --- |
| **Node** | Left hemispheric glioma  (mean ± SEM) | | *p* value | Right hemispheric glioma  (mean ± SEM) | | *p* value |
|  | Epilepsy | Non-epilepsy |  | Epilepsy | Non-epilepsy |  |
| A6m_L | 0.006 ± 0.003 | 0.009 ± 0.003 | 0.4963 | 0.007 ± 0.003 | 0.005 ± 0.005 | 0.7488 |
| A6m_R | -0.002 ± 0.002 | 0.000 ± 0.002 | 0.4199 | 0.003 ± 0.005 | 0.006 ± 0.002 | 0.4892 |
| A4hf_L | 0.006 ± 0.003 | 0.011 ± 0.004 | 0.4293 | 0.009 ± 0.003 | 0.009 ± 0.003 | 0.9972 |
| A4hf_R | 0.008 ± 0.003 | 0.014 ± 0.003 | 0.1565 | 0.002 ± 0.003 | 0.007 ± 0.005 | 0.4220 |
| A6cdl_L | 0.001 ± 0.002 | 0.007 ± 0.003 | 0.1393 | 0.002 ± 0.003 | 0.004 ± 0.003 | 0.5086 |
| A6cdl_R | 0.001 ± 0.002 | 0.007 ± 0.003 | 0.1771 | 0.002 ± 0.003 | 0.008 ± 0.005 | 0.3948 |
| A4ul_L | -0.006 ± 0.004 | 0.015 ± 0.007 | 0.0227 | 0.010 ± 0.007 | 0.013 ± 0.005 | 0.8036 |
| A4ul_R | 0.004 ± 0.003 | 0.007 ± 0.004 | 0.6263 | -0.001 ± 0.003 | 0.010 ± 0.004 | 0.0372 |
| A4t_L | 0.009 ± 0.004 | 0.007 ± 0.005 | 0.8330 | 0.009 ± 0.004 | 0.010 ± 0.004 | 0.7832 |
| A4t_R | 0.005 ± 0.004 | 0.000 ± 0.002 | 0.2523 | 0.013 ± 0.005 | 0.009 ± 0.004 | 0.5049 |
| A4tl_L | 0.000 ± 0.002 | 0.003 ± 0.002 | 0.3934 | 0.009 ± 0.002 | 0.007 ± 0.004 | 0.7101 |
| A4tl_R | 0.010 ± 0.005 | 0.008 ± 0.003 | 0.7542 | 0.008 ± 0.003 | 0.008 ± 0.004 | 0.9893 |
| A6cvl_L | 0.002 ± 0.003 | 0.003 ± 0.003 | 0.8255 | 0.003 ± 0.003 | 0.002 ± 0.004 | 0.7911 |
| A6cvl_R | 0.008 ± 0.004 | 0.005 ± 0.003 | 0.5342 | 0.001 ± 0.002 | 0.005 ± 0.003 | 0.3719 |
| A1/2/3ulhf_L | 0.015 ± 0.005 | 0.007 ± 0.003 | 0.1381 | 0.022 ± 0.007 | 0.013 ± 0.003 | 0.2043 |
| A1/2/3ulhf_R | 0.009 ± 0.004 | 0.009 ± 0.004 | 0.9276 | 0.022 ± 0.006 | 0.001 ± 0.003 | 0.0033 |
| A1/2/3tonIa_L | 0.010 ± 0.004 | 0.003 ± 0.003 | 0.2242 | -0.001 ± 0.004 | 0.013 ± 0.009 | 0.2252 |
| A1/2/3tonIa_R | 0.005 ± 0.004 | 0.006 ± 0.003 | 0.8742 | 0.001 ± 0.002 | 0.010 ± 0.006 | 0.1764 |
| A2_L | 0.008 ± 0.005 | 0.011 ± 0.003 | 0.5939 | 0.001 ± 0.004 | 0.017 ± 0.005 | 0.0454 |
| A2_R | 0.010 ± 0.003 | 0.009 ± 0.003 | 0.9443 | 0.004 ± 0.003 | 0.004 ± 0.002 | 0.8926 |
| A1/2/3tru_L | 0.012 ± 0.004 | 0.008 ± 0.004 | 0.5464 | 0.02 ± 0.006 | 0.006 ± 0.003 | 0.0324 |
| A1/2/3tru_R | 0.007 ± 0.003 | 0.010 ± 0.003 | 0.4975 | 0.004 ± 0.007 | 0.006 ± 0.003 | 0.8239 |
| A23d_L | 0.012 ± 0.007 | 0.010 ± 0.004 | 0.7529 | 0.004 ± 0.003 | 0.005 ± 0.004 | 0.8030 |
| A23d_R | 0.003 ± 0.003 | 0.008 ± 0.003 | 0.2358 | 0.001 ± 0.002 | 0.015 ± 0.004 | 0.0072 |
| A4ll_L | -0.001 ± 0.002 | 0.001 ± 0.003 | 0.5745 | 0.004 ± 0.004 | -0.004 ± 0.001 | 0.0710 |
| A4ll_R | 0.001 ± 0.002 | 0.002 ± 0.002 | 0.8556 | 0.010 ± 0.004 | -0.002 ± 0.002 | 0.0167 |
| A1/2/3ll_L | 0.001 ± 0.002 | 0.005 ± 0.002 | 0.2274 | 0.005 ± 0.003 | 0.006 ± 0.004 | 0.8978 |
| A1/2/3ll_R | 0.010 ± 0.005 | 0.008 ± 0.003 | 0.7433 | 0.013 ± 0.003 | 0.018 ± 0.007 | 0.6065 |
| A23v_L | 0.009 ± 0.003 | 0.015 ± 0.004 | 0.2616 | 0.000 ± 0.003 | 0.008 ± 0.003 | 0.0775 |
| A23v_R | 0.011 ± 0.005 | 0.002 ± 0.003 | 0.1282 | 0.006 ± 0.006 | 0.005 ± 0.003 | 0.8388 |
| A24cd_L | 0.010 ± 0.004 | 0.010 ± 0.005 | 0.9721 | 0.01 ± 0.004 | 0.010 ± 0.003 | 0.9316 |
| A24cd_R | 0.008 ± 0.003 | 0.007 ± 0.003 | 0.8115 | 0.011 ± 0.005 | 0.014 ± 0.006 | 0.7723 |
| A23c_L | 0.004 ± 0.003 | 0.007 ± 0.004 | 0.6113 | 0.002 ± 0.003 | 0.002 ± 0.002 | 0.9561 |
| A23c_R | 0.008 ± 0.003 | 0.007 ± 0.004 | 0.8860 | 0.013 ± 0.006 | 0.008 ± 0.005 | 0.4908 |
| A32sg_L | 0.009 ± 0.003 | 0.007 ± 0.004 | 0.6795 | 0.007 ± 0.006 | 0.005 ± 0.003 | 0.7468 |
| A32sg_R | 0.008 ± 0.002 | 0.012 ± 0.003 | 0.3227 | 0.011 ± 0.004 | 0.005 ± 0.003 | 0.2709 |
| mPMtha_L | 0.005 ± 0.004 | 0.001 ± 0.003 | 0.3826 | -0.004 ± 0.002 | 0.006 ± 0.003 | 0.0117 |
| mPMtha_R | 0.014 ± 0.006 | 0.005 ± 0.005 | 0.2534 | 0.006 ± 0.003 | 0.004 ± 0.004 | 0.7331 |
| Stha_L | -0.002 ± 0.002 | 0.009 ± 0.003 | 0.0057 | 0.002 ± 0.002 | -0.006 ± 0.002 | 0.0246 |
| Stha_R | 0.010 ± 0.003 | -0.002 ± 0.003 | 0.0053 | 0.003 ± 0.005 | 0.001 ± 0.002 | 0.6912 |
| * SEM, standard error mean. L, left hemispheric node; R, right hemispheric node. | | | | | | |

| **Table s29.Factors for preoperative GRE onset through binary logistic regression analysis with all patients** | | | | | | |
| --- | --- | --- | --- | --- | --- | --- |
| **Demographic and clinical characteristics** | **Univariate analysis** | | | **Multivariate analysis** | | |
|  | *p* value | Odd ratio | 95% CI | *p* value | Odd ratio | 95% CI |
| **Age** | 0.046 | 0.968 | 0.938 to 0.999 | - | - | - |
| **Sex (reference: female)** | 0.268 | 1.444 | 0.754 to 2.766 | - | - | - |
| **Tumor volume** | 0.320 | 0.992 | 0.976 to 1.008 | - | - | - |
| **Histopathological grade (reference: not grade 2)** | 0.007 | 2.544 | 1.238 to 5.041 | - | - | - |
| **IDH status (reference: IDH wildtype)** | < 0.001 | 7.960 | 2.633 to 23.799 | < 0.001 | 7.960 | 2.633 to 23.799 |
| **Chromosome 1p/19q status (reference: intact)** | 0.428 | 1.302 | 0.678 to 2.500 | - | - | - |
| * Multivariate analysis was: forward, condition. | | | | | | |

# Reference

**1.** Calhoun VD, Wager TD, Krishnan A, et al. The impact of T1 versus EPI spatial normalization templates for fMRI data analyses. *Hum Brain Mapp.* 2017; 38(11):5331-5342.

**2.** Nakajima R, Kinoshita M, Yahata T, Nakada M. Recovery time from supplementary motor area syndrome: relationship to postoperative day 7 paralysis and damage of the cingulum. *J Neurosurg.* 2019:1-10.
